# RelB/ILF2–GM-CSF loop sustains tumor–MDSC communication in prostate cancer immunosuppression

**DOI:** 10.1038/s41419-026-09098-x

**Published:** 2026-07-11

**Authors:** Fan Xu, Min Li, Yuan Zhang, Zhi Xu, Qiming Yin, Sijia Zhang, Fei Wu, Yuyan Ma, Zhangjun Jia, Xiumei Wang, Jiachen Dong, Ziyu Zhou, Jian Jian Li, Xiao Li, Yanyan Zhang

**Affiliations:** 1 Jiangsu Key Laboratory of Innovative Cancer Diagnosis & Therapeutics, Jiangsu Cancer Hospital, Jiangsu Institute of Cancer Research, The Affiliated Cancer Hospital of Nanjing Medical University, Nanjing, China; 2 Cancer Center, Zhongshan Hospital, Fudan University, Shanghai, China; 3 Phase I Clinical Trials Unit, Nanjing Drum Tower Hospital, Affiliated Hospital of Medical School, Nanjing University, Nanjing, China; 4 Nanjing Medical University, Nanjing, China; 5 Jiangsu Cancer Hospital, Jiangsu Institute of Cancer Research, The Affiliated Cancer Hospital of Nanjing Medical University, Nanjing, China; 6 Department of Oncology, Nanjing First Hospital, Nanjing Medical University, Nanjing, China; 7 Department of Radiation Oncology, University of California Davis Medical Center, Sacramento, CA USA; 8 Department of Urologic Surgery, Jiangsu Cancer Hospital, Jiangsu Institute of Cancer Research, The Affiliated Cancer Hospital of Nanjing Medical University, Nanjing, China; 9 Department of Scientific Research, Jiangsu Cancer Hospital, Jiangsu Institute of Cancer Research, The Affiliated Cancer Hospital of Nanjing Medical University, Nanjing, China

**Keywords:** Cancer microenvironment, Prostate cancer, Oncogenesis

## Abstract

Prostate cancer (PCa) exhibits an immune “cold” tumor microenvironment (TME) with significant enrichment of immunosuppressive myeloid-derived suppressor cells (MDSCs), which contribute to tumor progression and poor responses to immune checkpoint blockade (ICB). Although extensively studied, the precise mechanism underlying the tumor-intrinsic signals that educate and sustain the expansion and immunosuppressive function of MDSCs remains unelucidated. Here, we identify a previously unrecognized function of RelB by which nuclear interaction with ILF2 restricts ubiquitin-mediated degradation and sustains nuclear stability of RelB, thereby enhancing RelB-mediated *CSF2* transactivation and enhanced GM-CSF (granulocyte-macrophage colony-stimulating factor) production and secretion. The secreted GM-CSF, in turn, promotes RelB nuclear accumulation, forming a feed-forward loop for sustaining GM-CSF generation. In MDSCs, GM-CSF activates STAT3-mediated MDSC expansion and immunosuppression. Disruption of RelB/ILF2 complex attenuates GM-CSF-driven MDSC expansion, restores CD8⁺ T cell-mediated antitumor activity, and inhibits tumor growth. In addition, a RelB-targeting peptide SN52 sensitizes PCa tumors to PD-1 blockade therapy. Collectively, our findings identify the RelB/ILF2–GM-CSF loop as a central regulator of MDSC-mediated immunosuppression, which may be targeted to improve PCa response to ICB.

**Figure:**
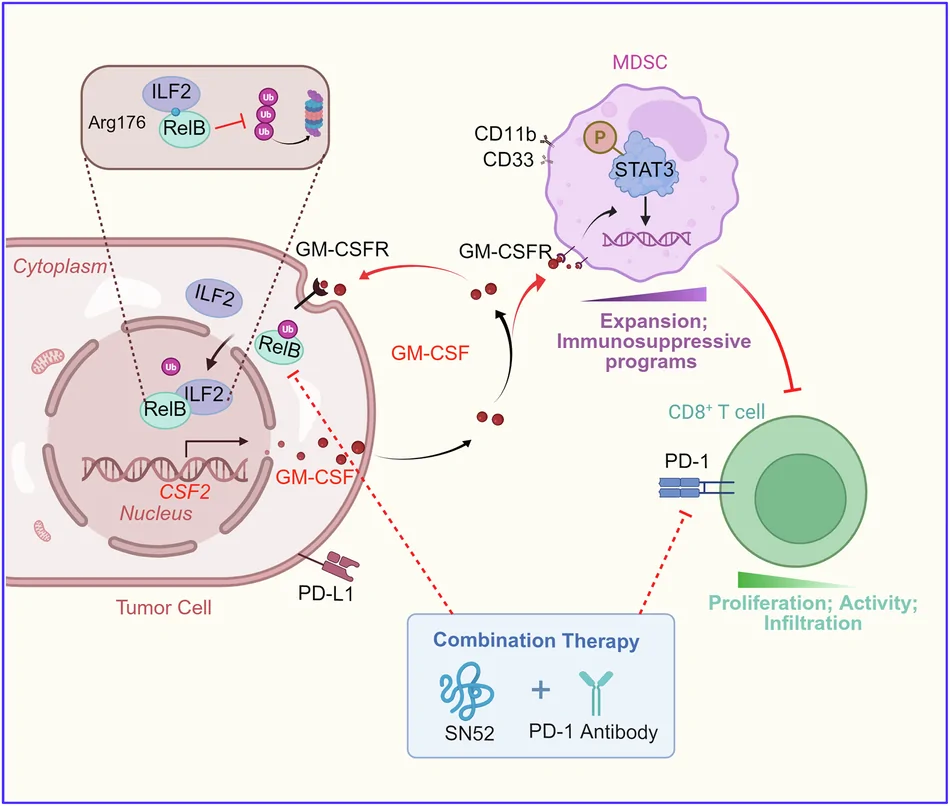

## Introduction

Prostate cancer (PCa) remains the most common malignancy among men worldwide, with a steadily rising annual incidence [1–4]. Despite standard treatment with androgen deprivation therapy (ADT) with or without radiotherapy, many patients ultimately progress to the stage of castration-resistant prostate cancer (CRPC), leading to the lethal condition of metastasis [5, 6]. Targeting CRPC, including blocking androgen synthesis by the steroid sulfatase pathway [7], still fails to achieve durable clinical benefit in many cases. In addition, immune checkpoint blockade (ICB) targeting CTLA-4 or PD-1/PD-L1 axis, despite its remarkable efficacy in various other solid tumors [8], has shown limited therapeutic responses in PCa [9, 10]. Currently, no significant improvement is observed in the clinical trials with PD-1 inhibitors, including nivolumab and pembrolizumab [11, 12]. Additionally, both a phase II trial of ipilimumab (anti-CTLA-4) combined with radiotherapy and a phase III trial in post-chemotherapy patients showed no therapeutic improvement over placebo [13], further evidencing the immunosuppressive [12, 14] and immune feature [15, 16] of PCa.

It has been well-investigated that myeloid-derived suppressor cells (MDSCs), a heterogeneous population of immature myeloid cells classified into polymorphonuclear MDSCs (PMN-MDSCs) and monocytic MDSCs (M-MDSCs), play an essential role in governing the immunosuppressive tumor microenvironment (TME) [17]. MDSCs have also been shown to contribute to ICB resistance by inhibiting T cell function [18, 19]. Clinical data show that high MDSC levels correlate inversely with ICB efficacy [17–20], including PCa, especially in patients resistant to GVAX/ipilimumab treatment [20]. Tumor-derived factors, including GM-CSF, G-CSF, and VEGF, are implicated in MDSC expansion, thereby reducing cytotoxic T lymphocyte (CTL)-mediated immune surveillance [21]. IL-8, induced by androgen deprivation, promotes myeloid infiltration and a tumor-aggressive phenotype [22]. Furthermore, MDSC-secreted IL-23 is shown to promote the aggressive phenotype of CRPC [23]. Together, these findings suggest a complex interplay between tumor and MDSC, whereas the critical upstream molecules that initiate and sustain the communication that maintains the immune “cold” state in PCa remain unelucidated. NF-κB signaling complex, encompassing both canonical (RelA/p50) and non-canonical (RelB/p52) pathways, is identified to play a key transcriptional regulator of inflammation, immune evasion, and therapy resistance [24]. NF-κB is also involved in MDSC expansion in multiple tumor models, including Ras-driven cancers [25] and ovarian cancer [26], and NF-κB inhibition shows promise in enhancing ICB efficacy in advanced PCa [9]. Radiation-induced MDSC expansion has recently been attributed to NF-κB-mediated induction of YTHDF, an m^6^A reader protein that regulates the translation efficiency and stability of m^6^A-modified RNAs [27].

NF-κB subunits have been indicated to display distinct biological functions depending on their dimerization partners [28], in which RelB functions as a critical regulator of immune response [29, 30] and also as a potential biomarker for immunotherapy across diverse human cancers [31]. RelB promotes the aggressive phenotype in PCa and contributes to both tumor radioresistance [32] and immunosuppression [33]. Herein, we elucidate a unique RelB-guided tumor–MDSC communication that sustains the immunosuppressive TME in PCa via RelB/ILF2–GM-CSF loop, which is shown to suppress PCa response to ICB.

## Results

### MDSCs are enriched in the PCa microenvironment

**Fig. 1:**
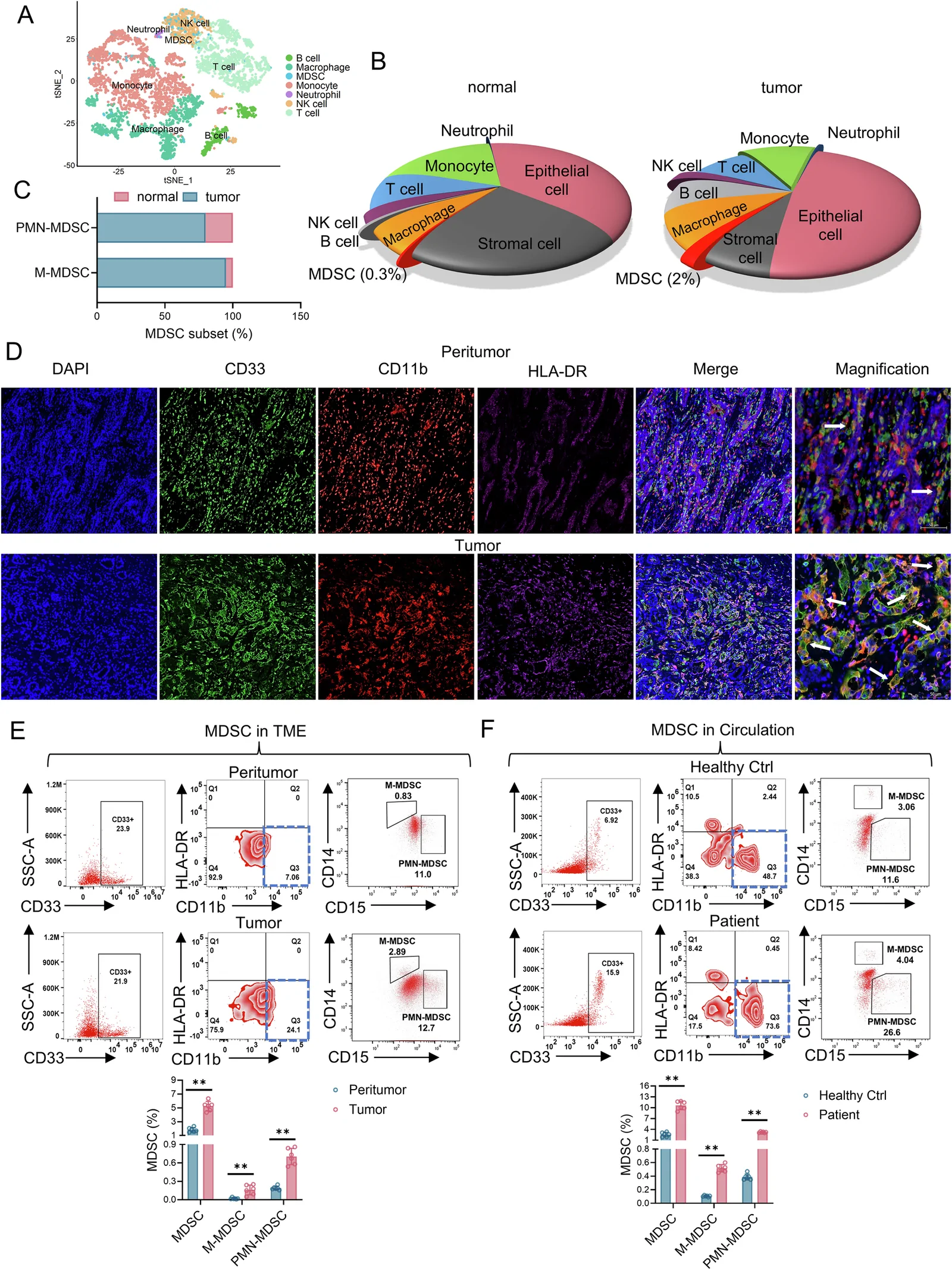
MDSCs are enhanced in both TME and circulation of prostate cancer patients. **A** Identification of immune cell subtypes based on scRNA-seq analysis. **B** Comparative pie distribution of MDSCs in normal (left) and PCa tissues (right). **C** Relative proportions of PMN-MDSC and M-MDSC in normal and tumor tissues. **D** Representative images showing MDSC infiltration in peritumor and tumor tissues from PCa patients. **E** Flow cytometry (upper panels) and quantitation (lower panels) of total MDSC, M-MDSC, and PMN-MDSC in peritumor and tumor tissue (*n* = 6). **F**. Flow cytometry (upper panels) and quantitation (lower panels) of total MDSC, M-MDSC, and PMN-MDSC in the systemic circulation of healthy individuals (*n* = 6) and patients with diagnosed PCa (*n* = 6). Data are shown as mean ± s.d., *P < 0.05, **P < 0.01; Student’s *t*-test (**E**, **F**).

From the single-cell RNA sequencing (scRNA-seq) data (GSE141445), we conducted an integrative analysis to characterize the landscape of normal and cancer tissues. The cells were meticulously stratified into nine distinct clusters (Fig. S1A), categorized into myeloid, lymphoid, epithelial, and stromal cell subsets, as illustrated in Fig. S1B. The immune cell populations were further dissected into seven individual clusters, wherein the presence of MDSCs was notably discerned (Fig. 1A). These results revalidate the significant increase in the proportion of MDSCs within PCa tissues (2%) in comparison to the non-cancerous counterparts in normal prostate tissues (0.3%), accompanied by increased epithelial and decreased stromal cells (Fig. 1B). Consistently, both PMN-MDSC and M-MDSC subsets were significantly enriched in tumor tissues (Fig. 1C). To validate these findings in clinical specimens, multiplex immunohistochemistry (mIHC) was performed on paired tumor and peritumor prostate tissues, which confirmed a marked increase of CD33^+^CD11b^+^HLA-DR^low/−^ MDSCs in tumors, indicating robust infiltration within the PCa TME (Fig. 1D). To quantitatively substantiate these findings, flow cytometry analysis of paired tumor and adjacent prostate tissues revealed an approximately threefold increase in MDSCs within tumors relative to peritumor tissues (Figs. 1E and S1C), with both M-MDSC and PMN-MDSC subsets consistently enriched. Furthermore, in line with prior reports [34], peripheral MDSCs were increased by approximately fourfold in PCa patients compared with age-matched healthy controls (Figs. 1F and S1D), accompanied by a parallel expansion of both M-MDSC and PMN-MDSC subsets. Based on these observations together with recently reported tumor cell–MDSC communication [35], we generated a multifaceted web of cellular interactions with MDSCs (Fig. S2A, B) in which the signaling network underlying tumor–MDSC communication is suggested to play a role in the immunosuppressive TME of PCa.

### RelB contributes to MDSC-related immunosuppression

**Fig. 2:**
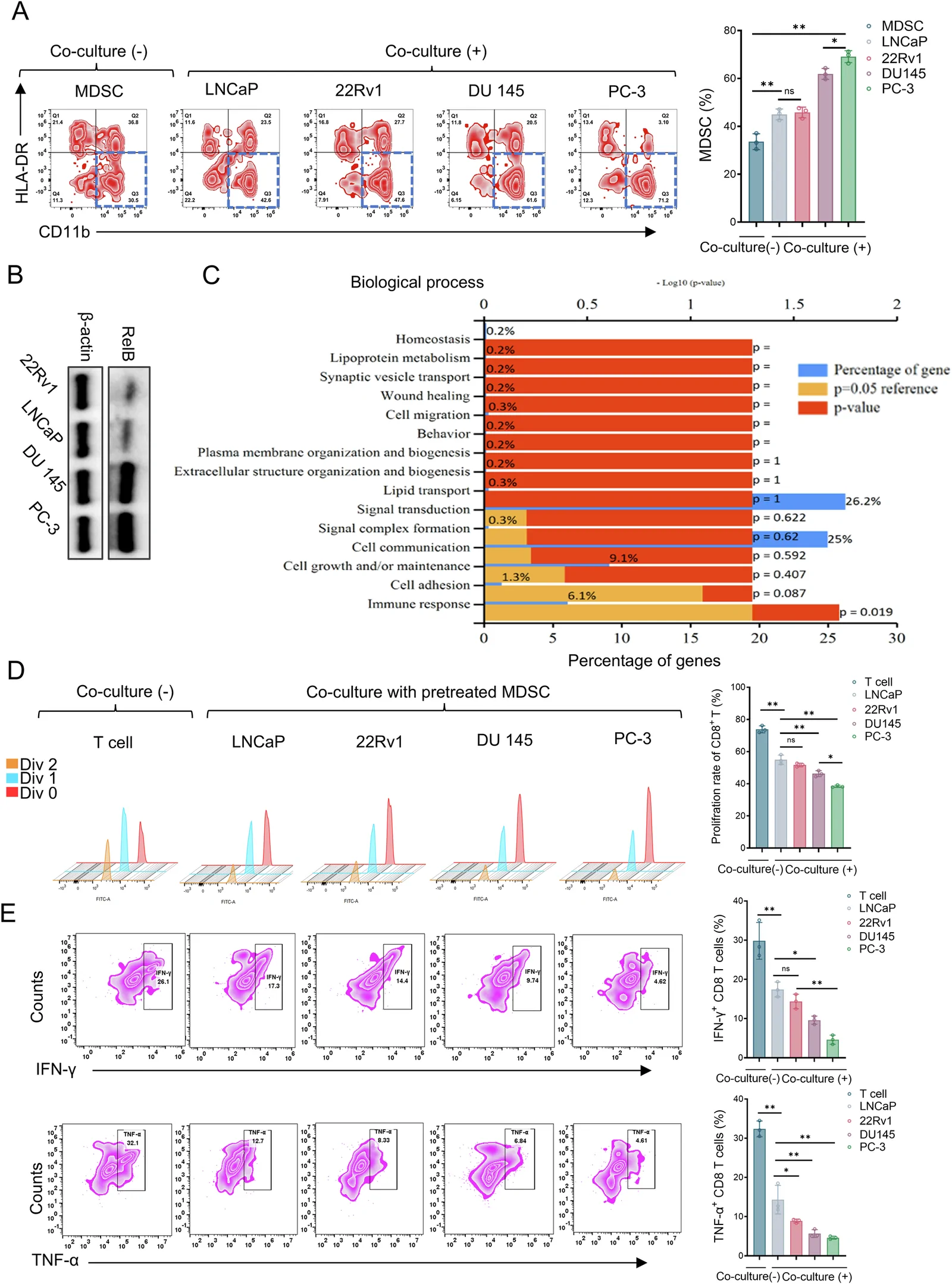
RelB signaling in PCa cells is involved in MDSC expansion. **A** Flow cytometry (left) and quantitation (right, *n* = 3) of MDSCs derived from CD33^+^ immune cells co-cultured with various PCa cells (LNCaP, 22Rv1, DU 145, PC-3). **B** Elevated RelB level in AR-negative DU 145 and PC-3 cells. **C** RNA-seq results referring to the crucial role of RelB in various biological processes by Gene Ontology (GO) analysis. **D** Flow cytometry (left) and quantitation (right, *n* = 3) of CD8^+^ T cell proliferation by CFSE analysis treated with MDSCs preconditioned by various PCa cells. **E** Flow cytometry (left) and quantitation (right, *n* = 3) of IFN-γ and TNF-α in CD8^+^ T cells treated with MDSCs preconditioned by various PCa cells. Data are shown as the mean ± s.d., *P < 0.05, **P < 0.01; one-way ANOVA (**A**, **D**, **E**).

To examine the effect of PCa cells on MDSC proliferation and immunosuppressive function, CD33^+^ immune cells isolated from peripheral blood mononuclear cells (PBMCs) were co-cultured with PCa cells differing in androgen receptor (AR) expression [36]. AR-negative PCa cells (PC-3 and DU145) led to a markedly greater expansion of MDSC compared to AR-positive cells (LNCaP and 22Rv1), although a moderate increase was also observed with the latter setting (Fig. 2A). RelB was remarkably enhanced in PC-3 and DU145 cells compared to LNCaP and 22Rv1 cells (Fig. 2B). RNA-seq-based Gene Ontology (GO) analysis in PC-3 cells with or without RelB knockdown identified that RelB was more closely involved in immune response processes than its relations to other biological pathways (Fig. 2C). Among the factors involved in immune regulation, RelB exhibited a predominant involvement in cytokine signaling within the immune system (Fig. S3A) with an affirmed role in this context (Fig. S3B). Interestingly, MDSCs exposed to the RelB-high cancer cells showed enhanced suppression on CD4⁺ and CD8⁺ T cells within the CD3⁺ T cell population compared to those pretreated with RelB-low cells (Fig. S4A). Importantly, the suppression of CD8^+^ T cell proliferation was more pronounced than that of CD4^+^ T cells (Figs. 2D and S4B, C). In addition, MDSCs preconditioned by RelB-high PC-3 cells exerted the strongest suppressive effects on T cell effector function, resulting in significantly reduced IFN-γ and TNF-α production, particularly in CD8⁺ T cells (Figs. 2E and S4D). These results indicate that RelB plays a role in enhancing PCa immunosuppressive TME by MDSC expansion and T cell impairment.

### RelB upregulates GM-CSF for increasing MDSC expansion

**Fig. 3:**
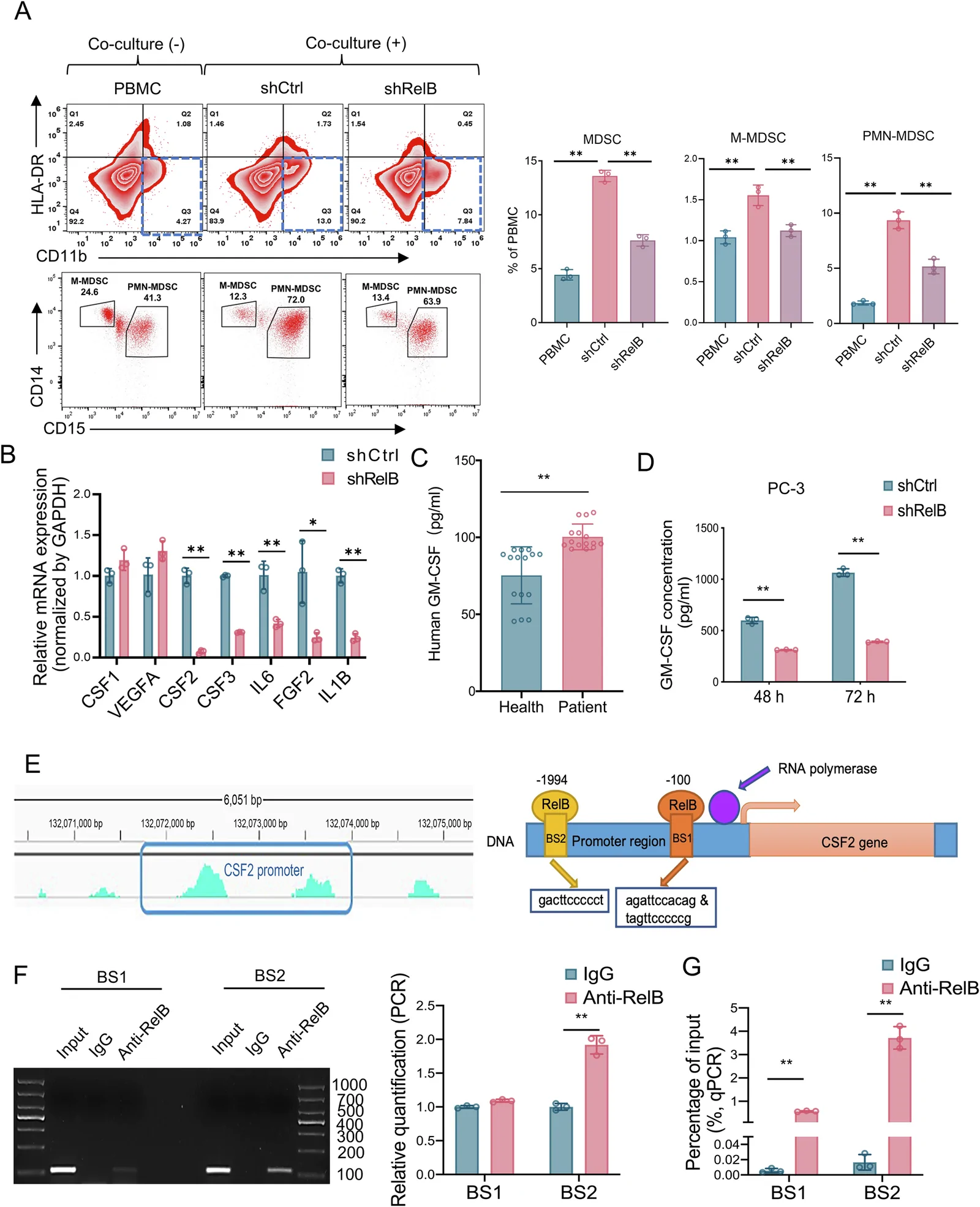
RelB-enhanced MDSCs via GM-CSF. **A** Flow cytometry (left) and quantitation (right, *n* = 3) of total MDSC, M-MDSC, and PMN-MDSC after co-culture of human PBMCs with PC-3 cells (wild-type or RelB knockdown) for three days. **B** CSF2 is among the RelB-regulated cytokines associated with MDSC expansion quantified by RT-qPCR (*n* = 3). **C** GM-CSF level measured in the circulation of PCa patients and healthy individuals (*n* = 15). **D** GM-CSF secretion in PC-3 cells under RelB inhibition for 48 and 72 h (*n* = 3). **E** Predicted RelB motif and the sequences of two binding sites (BS1 and BS2) located in the *CSF2* promoter region by IGV visualization (left) and JASPAR prediction (right). **F** Representative images (left) and quantitation (right, *n* = 3) of DNA fragments related to the *CSF2* promoter region (BS1, BS2) normalized by BS1. **G** Quantification of DNA fragments of BS1 and BS2 by RT-qPCR after ChIP assay (*n* = 3). Data are shown as mean ± s.d., *(*P* < 0.05) and **(*P* < 0.01) represent the level of significance; one-way ANOVA (**A**); Student’s *t*-test (**B**–**D**, **F**, **G**).

We then co-cultured PBMC with PC-3 in the presence or absence of RelB. RelB depletion in PC-3 cells significantly reduced MDSC expansion at day 3 (Fig. 3A) and day 7 (Fig. S5A), indicating that the function of RelB in tumor cells is required for tumor-induced MDSC expansion. Consistently, in a murine system, co-culture of RM-1 cells with splenocytes induced robust MDSC expansion, whereas genetic ablation of RelB in RM-1 cells markedly diminished the generation of splenocyte-derived MDSCs (Fig. S5B). Further subset analysis revealed that PC-3 cells promoted the expansion of both M-MDSC and PMN-MDSC populations. RelB knockdown significantly reduced both subsets, with M-MDSCs nearly returning to baseline levels, while PMN-MDSCs remained partially elevated (Fig. 3A). Together, these findings indicate that RelB plays a dominant role in driving M-MDSC expansion and also contributes to PMN-MDSC accumulation in the PCa microenvironment. RNA sequencing (Fig. S5C) followed by reverse transcription quantitative PCR (RT-qPCR) validation (Fig. 3B) revealed that RelB knockdown induced transcriptional changes associated with MDSC expansion, among which CSF2 (GM-CSF) showed the most pronounced downregulation. In line with these findings, GM-CSF expression in PCa tissues positively correlated with RelB levels (*r* = 0.321, *P* < 0.01), whereas no significant association was observed with RelA (*r* = 0.094, *P* > 0.01, Fig. S3C). Functionally, rescue experiments demonstrated that re-expression of GM-CSF most effectively restored MDSC expansion in RelB-silenced cells compared with other candidate cytokines (Fig. S5D), indicating that GM-CSF acts as a dominant downstream effector of RelB in promoting MDSC accumulation. Of note, GM-CSF concentration in the circulation of PCa patients was substantially elevated compared to the level in control healthy individuals (Fig. 3C). This result was consistent with the findings that reduced RelB expression decreased GM-CSF secretion within a 48-h timeframe in PC-3 and RM-1 cells and an extended 72 h period in PC-3 cells after RelB inhibition (Figs. 3D and S5E). By bioinformatic predictions, we identified two potential RelB motif elements situated within the promoter region of *CSF2* (Fig. 3E). Chromatin immunoprecipitation (ChIP) assays followed by PCR (Fig. 3F) and RT-qPCR (Fig. 3G) indicate that the distant binding site 2 (BS2) exhibits more pronounced involvement in the transcriptional process compared to binding site 1 (BS1).

**Fig. 4:**
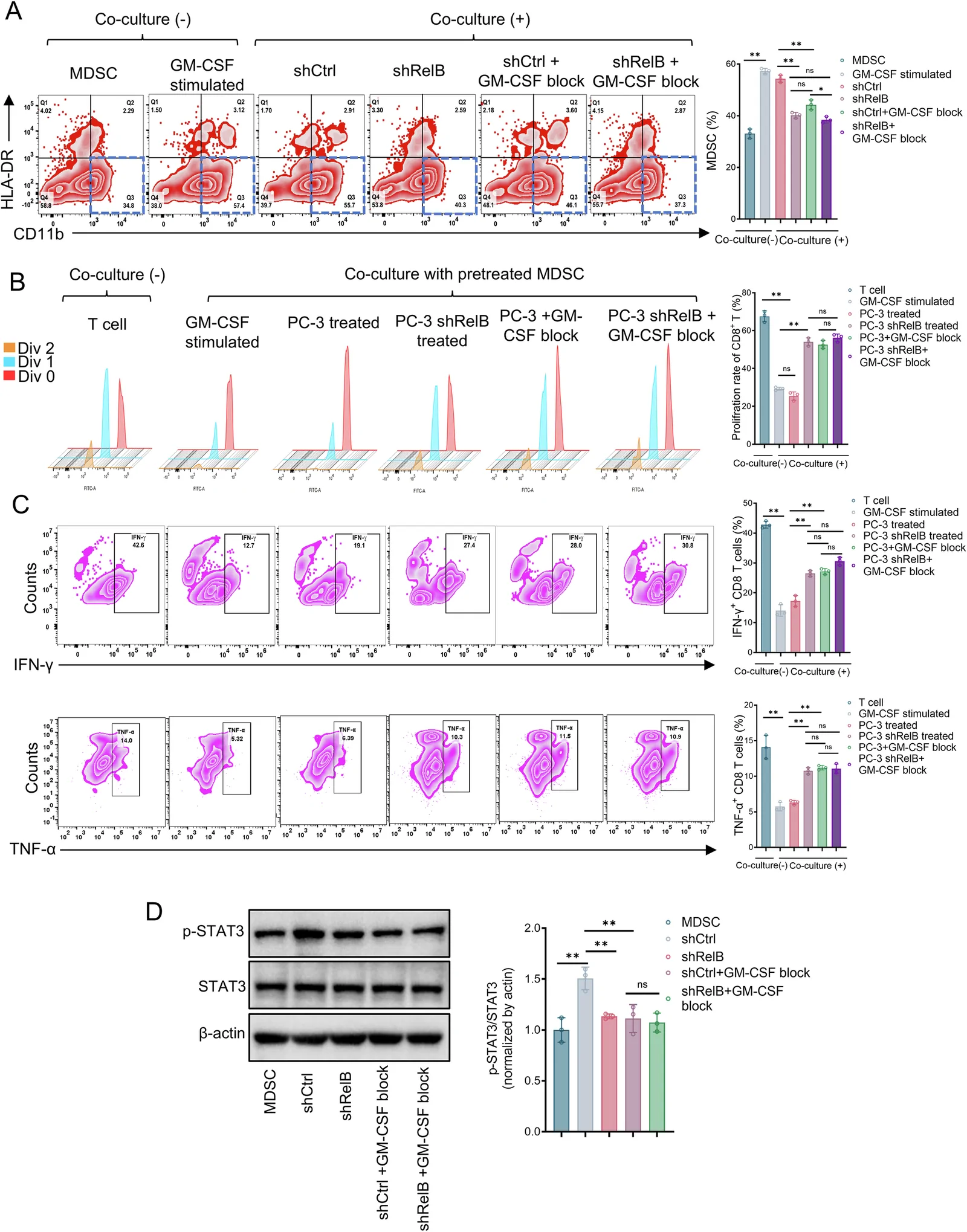
RelB–GM-CSF signaling promotes MDSC immunosuppressive function. **A** Flow cytometry (left) and quantitation (right, *n* = 3) of MDSCs treated with GM-CSF or co-cultured with PC-3 cells after indicated treatments. Representative flow cytometry (left) and quantitation (right) of CD8^+^ T cells (**B**, *n* = 3) and IFN-γ, TNF-α levels in CD8^+^ T cells (**C**, *n* = 3) co-cultured with MDSCs following co-culture with PC-3 cells with indicated treatments. **D** The expression (left) and quantification (right) of p-STAT3, STAT3 in MDSCs co-cultured with PC-3 cells after indicated treatments. Data are shown as the mean ± s.d., *P < 0.05, **P < 0.01; one-way ANOVA (**A**–**D**).

To further investigate RelB-associated MDSC expansion mediated by GM-CSF, CD33^+^ cells were isolated from PBMCs of healthy donors and co-cultured with PC-3 cells at a 10:1 ratio. Blockade of GM-CSF by neutralizing antibody in the MDSC/PC-3 co-culture system significantly inhibited MDSC expansion (Fig. 4A). In contrast, antibody-mediated GM-CSF blockade had no significant effect on MDSC expansion when MDSCs were co-cultured with PC-3 cells in which RelB was knocked down. To assess the functional consequence of these MDSCs, cells derived from the above co-culture conditions were subsequently isolated and co-cultured with T cells. MDSCs generated in the presence of RelB-competent PC-3 cells exhibited strong immunosuppressive activity, whereas GM-CSF blockade markedly attenuated this effect, as evidenced by restored CD8⁺ T cell proliferation (Fig. 4B) and increased IFN-γ and TNF-α production (Fig. 4C). Notably, GM-CSF blockade had minimal additional impact on the immunosuppressive function of MDSCs generated from RelB-knockdown PC-3 cells, further supporting that GM-CSF acts as a critical downstream mediator of RelB in this context. Mechanistically, RelB-mediated MDSC expansion via GM-CSF was associated with activation of the STAT3 signaling pathway (Fig. 4D). Together, these results indicate that RelB-driven GM-CSF production promotes the generation of functionally suppressive MDSCs, which in turn inhibit T cell responses, at least in part through STAT3 signaling.

### RelB/ILF2 heterodimer for *CSF2* transactivation

**Fig. 5:**
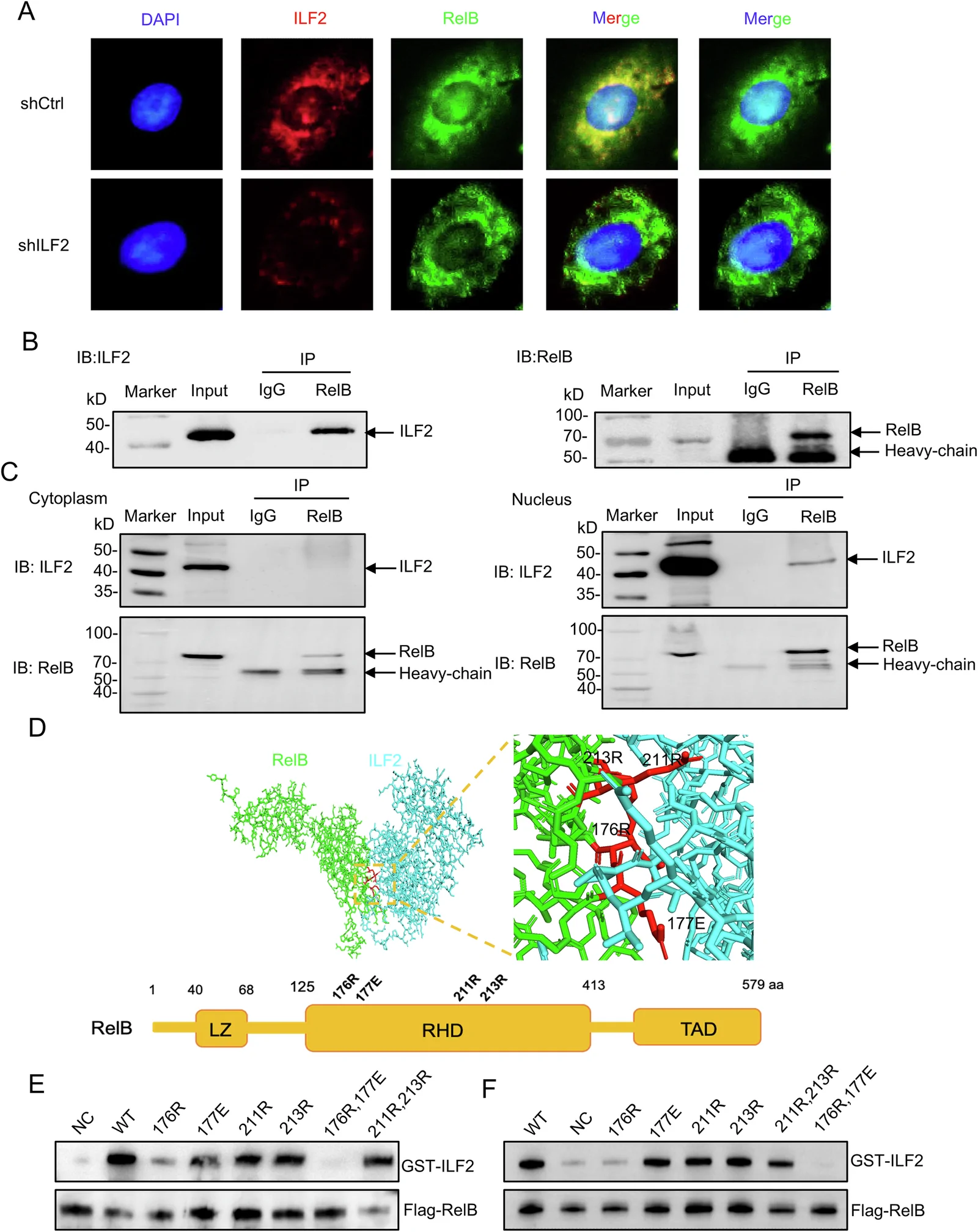
RelB/ILF2 complex regulates GM-CSF. **A** The localization of ILF2 (red) and RelB (green) in PC-3 cells by confocal microscopy. **B** The verification of the interaction between ILF2 and RelB by Co-IP assay in PC-3 cells. The protein was pulled down by RelB or IgG antibody, and the presence of RelB or ILF2 was detected by western blotting. **C** Co-IP assay was carried out in both the cytoplasm and nucleus of PCa cells, and RelB or ILF2 was detected by western blotting. **D** The three-dimensional structure of RelB (green) and ILF2 (blue) was generated using Z-DOCK and PyMOL software, and the potential binding sites (red) and corresponding mutant amino acids in the RHD of RelB were identified. **E** Exogenous GST-ILF2 and Flag-RelB (wild-type or mutant-type) plasmid were co-transfected into cells. RelB was precipitated with Flag antibody, and ILF2 was detected with GST antibody. **F** A pull-down assay was performed by incubating purified GST-ILF2 extracellularly with Flag-RelB (wild-type or mutant-type), and GST-ILF2 was detected after precipitation with Flag-RelB.

We then wondered if a specific RelB-containing complex is involved in *CSF2* transactivation. Co-immunoprecipitation (Co-IP) followed by silver staining and LC–MS/MS analysis identified 24 candidate proteins with molecular weights of 25 to 45 kD that potentially associate with RelB (Fig. S6A and Table S1). Among these candidates, ILF2 was prioritized due to its reported roles in transcriptional regulation and tumor progression, with a molecular weight of approximately 43 kD (Fig. S6B, C). Of note, ILF2 expression was significantly elevated across multiple malignancies, including PCa (Fig. S7A). Immunofluorescence revealed that both ILF2 and RelB were distributed in the cytoplasm and nucleus. Importantly, ILF2 depletion markedly reduced nuclear RelB levels without substantially affecting its cytoplasmic abundance, suggesting that ILF2 may regulate the nuclear localization or stability of RelB (Fig. 5A). To determine whether ILF2 physically interacts with RelB, Co-IP assay was performed. ILF2 was readily detected in RelB immunoprecipitates (Fig. 5B). Although the IgG heavy chain (~55 kD) was also present, it did not interfere with the identification of the target protein. Subcellular fractionation further revealed that ILF2 was detectable in RelB immunoprecipitates predominantly in the nuclear fraction, whereas little to no interaction was observed in the cytoplasmic fraction. In contrast, RelB itself was present in both compartments (Fig. 5C). Collectively, these results suggest that ILF2 interacts with RelB primarily in the nucleus and may facilitate RelB-dependent transcriptional activation of *CSF2*.

Based on these observations, we next tested potential interaction sites within the RelB/ILF2 complex. Sequence and structural analysis indicated that the Rel homology domain (RHD) of RelB, a highly conserved region within the NF-κB subunits responsible for DNA binding and protein interactions, contains several candidate residues, including Arg176, Glu177, Arg211, and Arg213, that could have an affinity to ILF2 (Fig. 5D). Flag-tagged wild-type or mutant RelB constructs, together with GST-tagged ILF2, were co-transfected into PC-3 cells (Fig. S7B). Co-IP assay revealed that mutation of Arg176 markedly impaired the interaction between RelB and ILF2, whereas mutations at other residues had less pronounced effects (Fig. 5E). Consistently, GST pull-down assays further confirmed that Arg176 is critical for the interaction between RelB and ILF2 (Figs. 5F and S7B). Together, these results identify Arg176 as a key residue mediating RelB/ILF2 complex formation.

### ILF2 stabilizes RelB to sustain a GM-CSF feed-forward loop

**Fig. 6:**
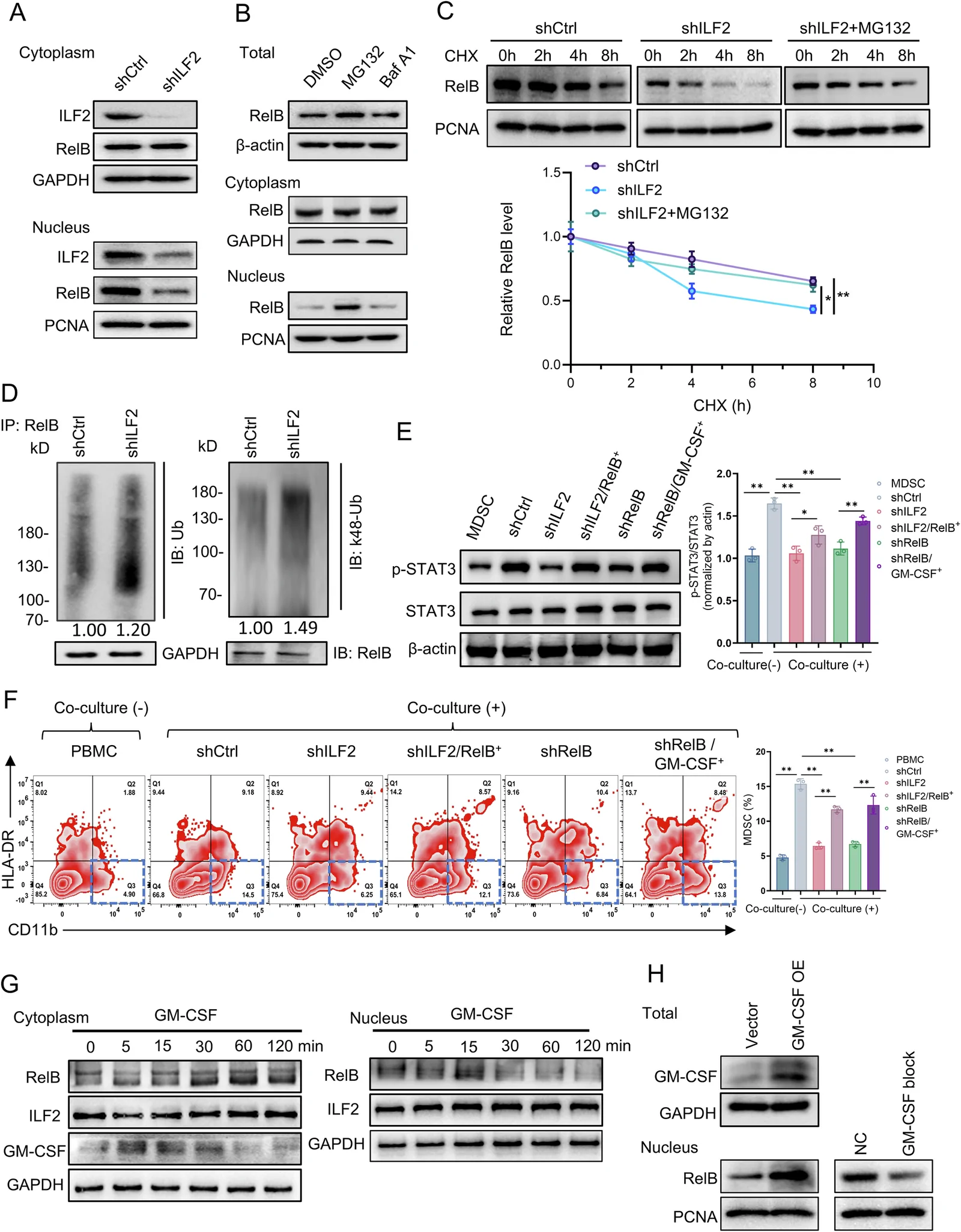
ILF2/RelB interaction and protein stability is linked to GM-CSF signaling. **A** Cytoplasmic and nuclear RelB and ILF2 in PC-3 cells with ILF2 knockdown. GAPDH and PCNA served as internal controls for the cytoplasmic and nuclear fractions, respectively. **B** RelB expression following treatment with MG132 (10 μM) or Bafilomycin A1 (Baf A1, 100 nM) for 6 h in shILF2 PC-3 cells. **C** RelB expression (upper panel) and corresponding quantification (lower panel) following cycloheximide (CHX) treatment at the indicated time points. **D** Total ubiquitination and K48-specific ubiquitination were assessed after immunoprecipitation of RelB in the presence or absence of ILF2. The immunoblot analysis of GAPDH served as an input control, while immunoblot of RelB was employed as a positive control. **E** The expression of p-STAT3 and STAT3 was detected and quantified in MDSCs co-cultured with the indicated PC-3 cells. **F** Representative image (left) and quantitation (right, *n* = 3) of MDSCs after co-culturing with indicated PC-3 cells. **G** The expression levels of RelB, ILF2, and GM-CSF were examined in both cytoplasm and nucleus of PC-3 cells over the specified time, following the induction of GM-CSF. **H** RelB expression in PC-3 cells following GM-CSF overexpression or blockade. Data are shown as the mean ± s.d., *P < 0.05, **P < 0.01; one-way ANOVA (**C**, **E**, **F**).

Analysis of TCGA data revealed a positive correlation between RelB and ILF2 expression (Fig. S8A). Moreover, both RelB and ILF2 were more highly expressed in AR-negative prostate cancer cells compared with AR-positive cells (Fig. S8B). To further define their regulatory relationship, we examined the effects of ILF2 and RelB perturbation. Silencing ILF2 in PC-3 and RM-1 cells markedly reduced RelB protein levels, whereas RelB knockdown or knockout had no detectable effect on ILF2 expression (Fig. S8C, D). Meanwhile, inhibition of ILF2 significantly reduced nuclear RelB levels, while cytoplasmic RelB remained largely unchanged (Fig. 6A), suggesting that ILF2 functions as a regulator of RelB stability.

To further determine the mechanism underlying ILF2-mediated regulation of RelB stability, we examined whether RelB degradation occurs via the proteasomal or lysosomal pathway. ILF2-depleted PC-3 cells were treated with the proteasome inhibitor MG132 or the lysosome inhibitor bafilomycin A1 (Baf A1). MG132 treatment significantly restored RelB protein levels in both whole-cell lysates and nuclear fractions, whereas Baf A1 had minimal effect (Fig. 6B), indicating that RelB degradation is primarily mediated through the ubiquitin–proteasome pathway. Consistently, cycloheximide (CHX) chase assays showed that ILF2 knockdown accelerated the degradation of RelB in the nuclear fraction. Importantly, MG132 treatment effectively blocked the rapid loss of RelB in ILF2-depleted cells (Fig. 6C), further supporting a proteasome-dependent mechanism of RelB degradation. At the molecular level, ILF2 may bind RelB at Arg176, a residue adjacent to the major ubiquitination-associated residues Lys295/296, thereby attenuating RelB ubiquitination and proteasomal degradation (Fig. 6D). Further mutational analyzes showed that wild-type and mutant RelB constructs displayed comparable expression levels under MG132 treatment (Fig. S9A). Disruption of Lys295/296 in RelB reduced ubiquitination (Fig. S9B) and enhanced protein stability (Fig. S9C), whereas mutation of Arg176 had the opposite effect; the combined mutation partially rescued this phenotype. Consistent trends were observed in nuclear RelB abundance (Fig. S9D) and GM-CSF expression (Fig. S9E), supporting a model in which ILF2 stabilizes RelB by limiting ubiquitination associated with Lys295/296.

Via protein–protein interaction network analysis, we found that ILF2 does not directly regulate GM-CSF expression, whereas RelB functions as the primary transcriptional activator of GM-CSF (Fig. S10A, B). Consistent with this, ILF2 knockdown decreased GM-CSF secretion, whereas RelB overexpression restored GM-CSF levels (Fig. S10C). In co-culture assays, ILF2 or RelB inhibition in PC-3 cells significantly impaired MDSC expansion and reduced phosphorylated STAT3 (p-STAT3) levels (Figs. 6E, F and S11A). These effects were rescued by re-expression of RelB or GM-CSF, respectively, indicating that the ILF2/RelB–GM-CSF axis contributes to STAT3-dependent MDSC expansion.

To investigate whether GM-CSF, in turn, influences RelB enhancement, PC-3 cells were treated with recombinant GM-CSF followed by cytoplasmic and nuclear fractionation. GM-CSF stimulation induced a transient increase in nuclear RelB at early time points (15 min), followed by a decline, while cytoplasmic RelB gradually accumulated over time (30–120 min). In contrast, ILF2 levels remained largely unchanged in both compartments. Cellular GM-CSF expression showed a rapid induction at 5 min and decreased markedly by 60 min (Fig. 6G). By contrast, in RelB-knockdown PC-3 cells, GM-CSF stimulation failed to alter RelB or ILF2 localization or expression, and no induction of GM-CSF was observed (Fig. S11B). In addition, GM-CSF overexpression markedly increased nuclear RelB accumulation, whereas blockade of GM-CSF signaling significantly reduced nuclear RelB levels (Fig. 6H). These findings support a positive regulatory circuit between GM-CSF and RelB in PCa cells, reinforcing a sustained signaling loop that promotes MDSC expansion.

### Formation of RelB/ILF2–GM-CSF in tumor immunosuppression

**Fig. 7:**
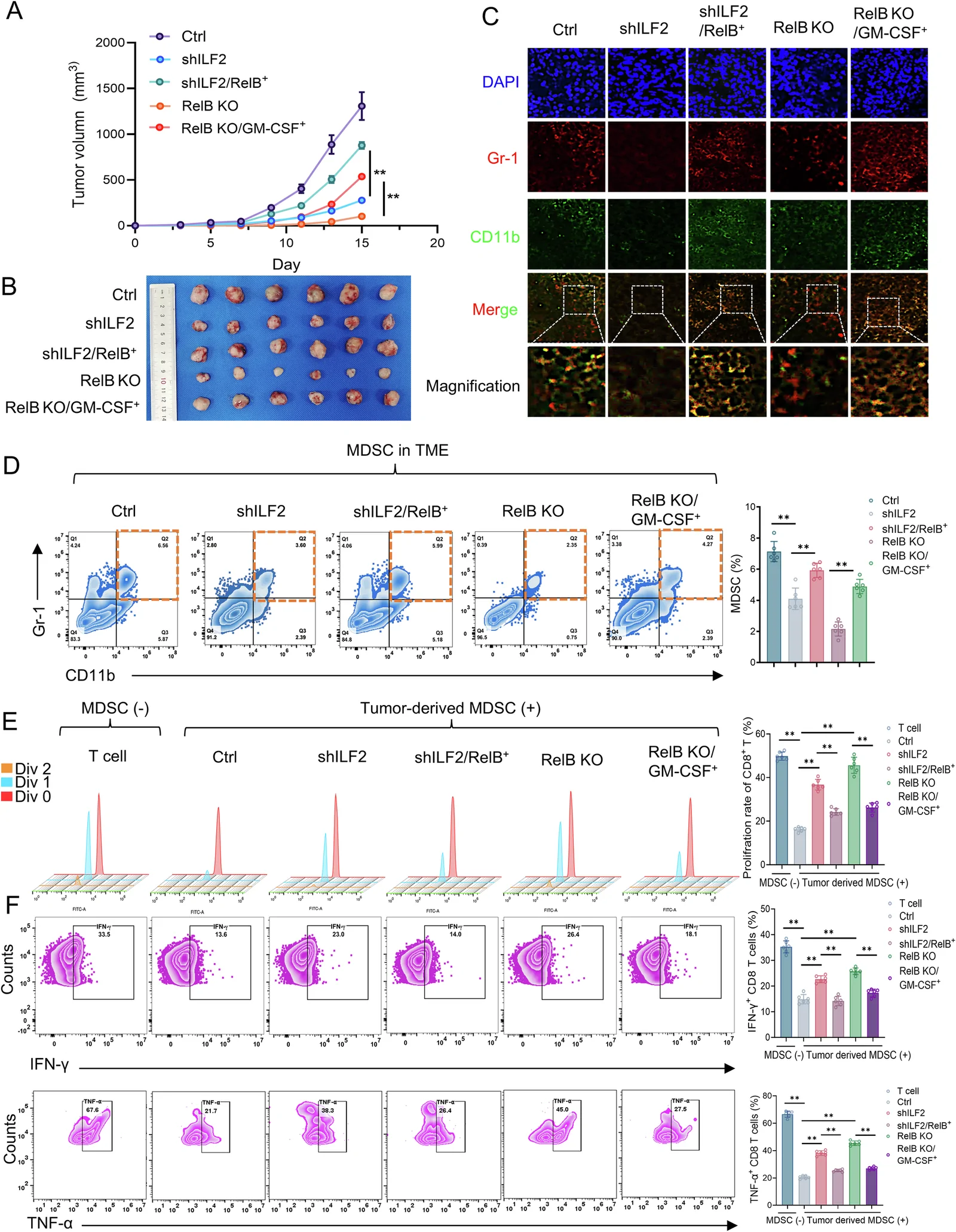
RelB/ILF2–GM-CSF loop promotes MDSC expansion in tumor-bearing mice. **A**, **B** Tumor volume in the indicated groups (*n* = 6). **C** Tumor-infiltrating MDSCs (Gr-1, red; CD11b, green) in tumors by immunofluorescence microscopy. **D** Representative image (left) and quantitation (right, *n* = 6) of tumor-infiltrating MDSCs in the manipulated tumors with different levels of ILF2, RelB, and GM-CSF expression. **E** Representative image (left) and quantitation (right, *n* = 6) of T cells proliferation co-cultured with sorted tumor-infiltrating MDSCs for three days. **F** Flow cytometry (left) and quantitation (right, *n* = 6) of IFN-γ and TNF-α positive CD8 T cells in the indicated groups. Data are shown as the mean ± s.d., *P < 0.05, **P < 0.01; one-way ANOVA (**A**, **D**–**F**).

We then tested whether RelB/ILF2 is activated in TME using the mouse tumor model (Fig. S12A). To confirm the stability of the cell models used in this experiment, we assessed ILF2 and RelB expression within tumor tissues. As shown in Fig. S12B, RelB expression was markedly reduced in tumors derived from ILF2-KD cells but was restored upon RelB reconstitution in ILF2-KD cells. In RelB-KO tumors, RelB levels remained low and were unaffected by GM-CSF overexpression. ILF2 expression decreased dramatically in tumors from both ILF2-KD cells and ILF2-KD/RelB-overexpressing cells, and was not influenced by either RelB or GM-CSF expression. In contrast, the reduced GM-CSF levels were substantially restored following RelB reconstitution in ILF2-KD cells or GM-CSF overexpression in RelB-KO cells. Tumors derived from wild-type RM-1 cells showed increased volume compared to those from ILF2-KD cells. Reconstitution of RelB in ILF2-KD cells restored tumor growth, while GM-CSF partially reversed the tumor volume reduction caused by RelB KO (Fig. 7A, B). Immunofluorescence (Fig. 7C) and flow cytometry (Fig. 7D) analyzes revealed that tumor-infiltrating MDSCs were markedly reduced in the ILF2-KD and RelB-KO groups compared with the control group. Restoring RelB or GM-CSF expression in the ILF2-KD or RelB-KO group effectively abolished the decline in MDSC levels. Consistent findings were observed in peripheral blood (Fig. S13A) and spleen (Fig. S13B). Notably, reduced MDSC abundance was accompanied by increased T cell infiltration into tumors (Fig. S14). Tumor-infiltrating MDSCs were sorted by flow cytometry and co-cultured with isolated T cells. Compared with wild-type control, MDSCs from ILF2-KD and RelB-KO tumors exhibited reduced suppression of CD8⁺ T cell proliferation and function. In contrast, MDSCs derived from RelB- or GM-CSF-overexpressing tumors markedly impaired CD8⁺ T cell activity (Fig. 7E, F). Proliferation of CD4⁺ T cells showed minimal differences across groups (Fig. S13C). However, MDSCs derived from the RelB-KO tumors partially restored CD4^+^ T cell function, whereas those from RelB KO/GM-CSF-overexpressing tumors diminished CD4⁺ T cell function (Fig. S13D).

### ICB with RelB inhibition enhances T cell function

**Fig. 8:**
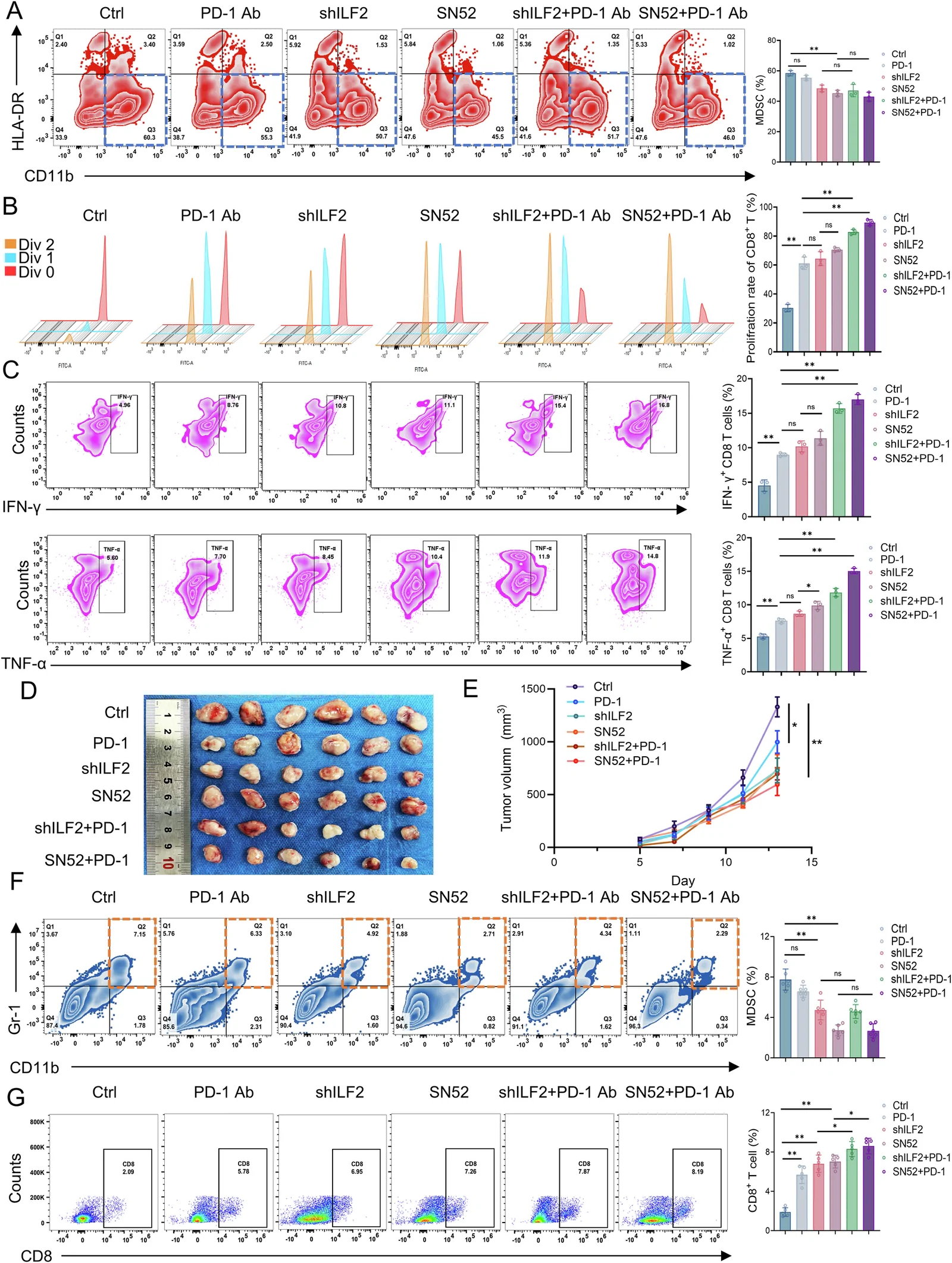
Targeting RelB reduces MDSCs in TME and enhances ICB response. **A** Flow cytometry (left) and quantitation (right, *n* = 3) of MDSC levels after co-culture of CD33^+^ cells with PCa cells (PC-3 or shILF2 PC-3) in the presence of PD-1 antibody and/or SN52. **B** Representative image (left) and quantitation (right, *n* = 3) of CD8^+^ T cell proliferation by co-culture of PCa cells with PBMCs in the presence of PD-1 antibody and/or SN52. **C** Representative image (left) and quantitation (right, *n* = 3) of IFN-γ and TNF-α positive CD8^+^ T cells in the indicated groups. **D**, **E** Representative image (left) and tumor volume (right) in the indicated groups (*n* = 6). **F** Representative image (left) and quantitation (right, *n* = 6) of tumor-infiltrating MDSCs in the specified groups. **G** Representative image (left) and quantitation (right, *n* = 6) of CD8^+^ T cell levels in the specified groups. Data are shown as the mean ± s.d., *P < 0.05, **P < 0.01; one-way ANOVA (**A**–**C**, **E**–**G**).

We then tested the effects of RelB inhibitor SN52 combined with anti-PD-1 antibody on MDSCs and T cells. CD33^+^ cells isolated from PBMCs were co-cultured with RelB-high PC-3 tumor cells. Compared to anti-PD-1 alone, SN52+anti-PD-1 significantly inhibited MDSC proliferation (Fig. 8A). The presence of SN52 or PD-1 antibody promoted CD4^+^ and CD8^+^ T cell proliferation, whereas SN52 exerted a stronger effect on CD8^+^ T cells, and SN52+anti-PD-1 further increased T cell proliferation (Figs. 8B and S15A, B). This was accompanied by a parallel restoration of T cell effector function, as indicated by elevated IFN-γ and TNF-α production, with the combination treatment exhibiting the strongest effect (Figs. 8C and S15C). In vivo tests, treatment with SN52+anti-PD-1 generated the maximal anti-tumor function compared to a single agent (Figs. S15D and 8D, E). The tumor-infiltrated MDSCs were significantly reduced, and CD8^+^ T cells were enhanced in the tumor treated by the combination treatment (Fig. 8F, G). These results suggest that blocking RelB upstream of the RelB/ILF2–GM-CSF loop is an efficient approach to reverse the immune “cold” phenotype and enhance PCa response to ICB.

## Discussion

This study uncovers a previously unrecognized RelB-centered pathway that enhances tumor–MDSC communication via heterodimerization with ILF2, thereby initiating a unique RelB/ILF2–GM-CSF loop. This loop promotes bidirectional signaling between tumor cells and MDSCs, sustaining high MDSC levels within the immunosuppressive TME of PCa. Disrupting this loop may significantly reverse immunosuppression and enhance the clinical benefit of ICB in the treatment of advanced PCa.

Chronic tumor-associated inflammation is shown to induce the activation of NF-κB signaling pathway [37] and also leads to the differentiation of myeloid cells into MDSC, linking to poor prognosis and resistance to immunotherapy [38]. We previously demonstrated that NF-κB subunit RelB is uniquely highly expressed and closely related to PCa progression and therapeutic resistance [32], as well as playing a key role in PCa immunoescape by modulating T cell activity in TME [33]. Our current findings highlight the role of tumor RelB in promoting tumor cell–MDSC communication in an in vitro cell co-culture system. These results indicate that T cell suppression by RelB is at least partially mediated by MDSCs, suggesting that targeting RelB could be a promising therapeutic strategy to overcome immune cell dysfunction in PCa.

We found that GM-CSF, a cytokine with well-documented roles in myeloid cell differentiation, plays a key role as a mediator in RelB-driven MDSC expansion. GM-CSF is a pleiotropic cytokine produced by a wide range of cell types, particularly tumor cells, under inflammatory conditions [39]. However, the impact of GM-CSF on immune response modulation depends on the context and magnitude of stimulation. Several reports have shown that cytokines originating from tumors serve as major mediators in the differentiation and induction process of MDSC [40, 41]. Specifically, irradiated tumor cells engineered to secrete GM-CSF elicit anti-tumor immunity mediated by T cells [42]. Previous studies have demonstrated that the canonical NF-κB subunit RelA mediates *CSF2* transcription [43] and that complex crosstalk exists between the canonical and non-canonical NF-κB pathways [44]. In the current study, we demonstrate that RelB independently drives *CSF2* transcription in PCa cells for promoting MDSC expansion.

Efforts to improve immunotherapy efficacy have increasingly focused on strategies to enhance immune activation [38, 45], particularly by depleting MDSCs [46]. However, the dominant MDSC subset in PCa remains debated. Hossain et al. reported that PMN-MDSCs (CD15⁺CD11b⁺CD33⁺48^low/−^) are the predominant circulating subset in both metastatic CRPC (mCRPC) and localized disease [47]. Conversely, other studies have linked elevated levels of M-MDSCs (CD14⁺CD11b⁺CD33⁺HLA-DR^low/−^) with prostate-specific antigen (PSA) levels and reduced overall survival in PCa patients [48]. In the current study, both M-MDSC and PMN-MDSC populations were elevated in peripheral blood and tumor tissues from PCa patients. RelB signaling promoted the expansion of both subsets, with a more pronounced effect on M-MDSCs, which are considered more suppressive than PMN-MDSCs on a per-cell basis [49]. These findings further support a central role for the RelB-driven signaling axis in shaping an immunosuppressive TME in PCa. The incomplete reduction of PMN-MDSCs following RelB inhibition also suggests that additional mechanisms may contribute to PMN-MDSC accumulation. Although B cell populations were also altered in the PCa microenvironment, MDSCs displayed greater relative enrichment and broader interactions with immune and stromal compartments, supporting their central role in immunosuppressive regulation. Collectively, our findings identify RelB as a critical mediator linking non-canonical NF-κB signaling to GM-CSF–driven MDSC expansion, while the potential contribution of B-cell subsets warrants further investigation.

This raised the critical question of how RelB achieves transcriptional control in the tumor context, leading us to identify ILF2 as an essential regulatory partner. RelB is inherently unstable and normally restrained by p100 or stabilized through heterodimerization with p50/p52 [37], but it can also engage alternative partners to fine-tune NF-κB target gene expression [50–53]. Our data demonstrate that ILF2, previously implicated in tumor proliferation [54] and drug resistance [55], directly binds the RelB RHD at Arg176, thereby stabilizing RelB within the nucleus and promoting *CSF2* transcription. ILF2 has been reported to stabilize other transcription factors such as CREB, suggesting a broader role in oncogenic signaling [56]. Post-translational modifications such as phosphorylation and ubiquitination are critically associated with distinct functions and pathological consequences [57–59], with polyubiquitination at Lys273/274 and Lys305/308 regulating RelB transcriptional activity in murine models [60]. Our findings identify ILF2 as a novel regulator of RelB stability that partially restrains ubiquitination associated with Lys295/296, which corresponds to Lys273/274 in murine RelB. By limiting K48-linked polyubiquitination, ILF2 attenuates proteasomal degradation of RelB, thereby sustaining its transcriptional activity and promoting MDSC expansion.

Our findings indicate that RelB functions as a central factor driving tumor–MDSC communication through a self-sustaining cytokine circuit. RelB promotes the transcription of GM-CSF, which not only recycles back to tumor cells to maintain RelB activity but also promotes MDSC expansion and functional activation via a STAT3-mediated pathway. GM-CSF has been reported to enhance other NF-κB signaling via histone acetylation and increased RelA expression [61], further amplifying inflammatory signaling within the TME. Thus, this RelB/GM-CSF-mediated reciprocal reinforcement could also be involved in such general protumor function with the unique function of sustaining a persistent tumor–MDSC crosstalk, promoting a highly immunosuppressive microenvironment in PCa. Collectively, these insights identify RelB as a pivotal regulator of cytokine-driven immune evasion and highlight its potential as a therapeutic target in PCa [62–66].

Disruption of RelB or ILF2 in PCa models resulted in a pronounced reduction in tumor growth, underscoring the translational potential of targeting this pathway. Importantly, inhibition of the RelB/ILF2–GM-CSF loop not only diminished tumor progression but also sensitized tumors to immune checkpoint therapy. Treatment with the RelB nuclear transport inhibitor SN52 or ILF2 knockdown significantly amplified anti-PD-1-boosted T cell proliferation and activation both in vitro and in vivo. Therefore, with the multiple approaches currently under investigation to target MDSCs [46], the discovery of RelB/ILF2–GM-CSF loop will present an alternative approach to block the main upstream signals causing MDSC expansion. TKI-based therapy remarkably reduces the MDSC-induced immunosuppression [67] and enhances the efficacy of PD-L1 inhibitor in patients with mCRPC [68]. Other combinatorial immunotherapeutic strategies have shown the potential to convert a “cold” tumor into a “hot” tumor [21]. However, a potential challenge remains that targeting the myeloid population alone may not be sufficient to achieve a significant anti-tumor effect. Indeed, even a phase I/II clinical trial combining a CD11b agonist with a PD-1 inhibitor achieved only modest responses, underscoring the need for alternative combination strategies [69]. Together, the therapeutic efficacy of disrupting the RelB/ILF2–GM-CSF loop, which can be achieved via RelB inhibition or blockade of related components, is to be further validated for PCa immunotherapy.

## Materials and methods

### Clinical sample

Fresh PCa tissues and adjacent tissues were collected from patients without chemoradiotherapy or endocrine therapy at The Affiliated Cancer Hospital of Nanjing Medical University (Nanjing, China). The tissues were dissected into 1–3 mm^3^ segments and treated with collagenase IV for 60 min. The resulting digest was filtered through a 70 μm cell sieve and analyzed using flow cytometry to detect the level of infiltrating MDSCs. To measure the MDSC levels in whole blood by flow cytometry, samples were collected from PCa patients and healthy individuals.

### Single-cell RNA-sequence (scRNA-seq)

The scRNA-seq data of human PCa and prostate tissues (GSE141445) were downloaded from the GEO database. For quality control, three quality measures were calculated: the percent of mitochondrial genes, the number of total genes, and housekeeping genes expressed. We removed cells that had more than 20% expression of mitochondrial genes, fewer than 200 total genes, or fewer than 56 housekeeping genes expressed. The resultant gene expression matrix was imported into the R (v3.5.1) statistical environment for further analysis. After normalization and scaling, the batch effect between patients was then removed using the mutual nearest neighbor algorithm in Seurat (v4.0.0). The batch-corrected matrix was used for further analysis and visualization.

### Identification of marker genes and cell type annotation

To identify differentially expressed marker genes for each cell type, the FindAllMarkers function in Seurat was used under default parameters. Marker genes were selected as those with adjusted *p* values less than 0.05, average logFC larger than 0.25, and percentage of cells with expression higher than 0.25. Immune cell types were annotated by the SingleR package (v1.10.0) and then checked manually. The marker genes or signature genes for cell types were as follows: epithelial cells (EPCAM, KRT8, KRT18, CDH1), endothelial cell (PECAM1, CD34, VWF, SELE), fibroblast (DCN, FBLN1), immune cells (PTPRC) including B cell (CD79A, MS4A1), MDSCs (FUT4, CD14, CD33, ITGAM, LYZ, ITGB2, IL1B, HLA-DRA), T cell (CD2, CD3E, CD3D, CD3G), natural killer cells (KLRB1, KLRC1, KLRD1, KLRF1, NCAM1), neutrophil (CXCR2, ADGRG3, KCNJ15, FCGR3B), monocyte (VCAN, FCN1, EREG, LYZ, C1QB), and macrophages (APOE, C1QA, C1QB), where endothelial cell and fibroblast were integrated into stromal here.

### Cell-cell communication analysis

To enable a comprehensive analysis of cell-cell communication molecules, cell-cell communication analysis was applied based on CellChat (v1.0.0). The ligand and receptor genes expressed by each cell were projected into a manually selected reference communication network, and the probability of communication in each pathway was inferred by gene expression. The communication probability between MDSC and other immune cells, epithelial cells, and stromal cells was statistically analyzed. Histograms were implemented with the ggplot2 package (v3.3.2) in R.

### RNA-seq analysis

RNA-seq was conducted as previously described [33]. Briefly, RNA-seq libraries were prepared using a VAHTS mRNA-seq v2 library Prep kit. Sequencing was performed on an Illumina HiSeq X Ten platform in a 150 bp paired-end mode. mRNA profiles were calculated using Cuffdiff software (v1.3.0) and presented as FPKM values. In addition, GO analysis was conducted to enrich biological processes in shRelB and shCtrl PC-3 cells.

### Bioinformatics

GEPIA (http://gepia.cancer-pku.cn/) was used to analyze ILF2 expression in multiple cancer types. TCGA (https://www.cancer.gov/about-nci/organization/ccg/research/structural-genomics/tcga) datasets were examined to assess the correlation between RelB and GM-CSF or ILF2. UCSC Genome Browser (http://genome.ucsc.edu/), ENCODE (https://www.encodeproject.org/), JASPAR database (https://jaspar.genereg.net/), and IGV software were used to predict binding sites of RelB to *CSF2* promoter region. SWISS-MODEL (https://swissmodel.expasy.org/), ZDOCK (http://zdock.umassmed.edu/) database and PyMOL 2.3.2 software (www.pymol.org) were applied to predict and visualize RelB/ILF2 binding sites in a three-dimensional structure. The protein-protein interaction network was developed by the STRING database (https://string-db.org/).

### Multiplexed immunohistochemistry (mIHC, fluorescence-based)

Multiplexed immunohistochemistry (mIHC) staining was performed on 4-μm-thick formalin-fixed, paraffin-embedded (FFPE) prostate cancer tissues and matched adjacent normal tissues. Sequential staining was conducted using a tyramide signal amplification (TSA) 7-color kit (abs50015, Absinbio, Shanghai, China) according to the manufacturer’s instructions. Briefly, tissue sections were deparaffinized, rehydrated, and subjected to antigen retrieval in citrate buffer (pH 6.0) using microwave heating. After blocking endogenous peroxidase activity, slides were incubated with primary antibodies against CD33 (STARTER, Cat#S0B2209, China), CD11b (Abcam, Cat#ab180127, UK), and HLA-DR (Absin, Cat#abs133250, China), followed by HRP-conjugated secondary antibodies. Signal detection was achieved using TSA fluorophores with sequential rounds of staining and heat-mediated stripping between each antibody cycle. MDSCs were identified as CD11b⁺CD33⁺HLA-DR^low/−^ cells. Nuclei were counterstained with DAPI. Stained slides were scanned using a Pannoramic MIDI II scanner (3DHISTECH).

### PCa cell transfection

Human PCa cells (PC-3, DU 145, LNCaP, 22Rv1) and murine PCa cells (RM-1) were purchased from American Type Culture Collection (ATCC, VA, USA) and were routinely tested for mycoplasma contamination. The cell lines were cultured in recommended RPMI 1640 media containing 10% FBS (Gibco, Cat#A3160801, MA, USA) and 1% penicillin/streptomycin (MCE, Cat#HY-K1006, NJ, USA). Short hairpin RNA (shRNA) and over-expression plasmids were stably transfected using the transfection reagent (Polypus, Cat#101000027, France). The sequences of shRNA and overexpression plasmids used for cell transfection are as follows: human-shRelB (RiboBio Co., Ltd, China), murine-RelB KO plasmid (Cas9-single guide RNA, 5′-GACGAATACATTAAGGAGAA-3′), shILF2 (5′-GCTATCTTGCTTCTGAAATAT-3′), RelB overexpression plasmid (pcDNA3.1-c3xFlag-RELB), and GM-CSF overexpression plasmid (TK-PCDH-copGFP-T2A-Puro) were purchased from Tsingke Biotechnology (China). RelB mutant plasmids (Tsingke Biotechnology) were transfected into RelB-knockdown cells using shRNAs targeting the 3′UTR region of endogenous RelB.

### Cell separation and co-culture

PBMCs were extracted from whole blood collected from healthy adult donors. Ficoll (Ficoll-Paque PLUS; GE, Cat#17-1440, Sweden) density gradient centrifugation was employed for the isolation of PBMCs from the whole blood. The obtained PBMCs were then co-cultured with PC-3 cells at a ratio of 10:1 for 3 or 7 days. For the mouse co-culture model, murine-derived spleen immune cells and syngeneic murine PCa cells (RM-1) were utilized.

### Flow cytometry

Single-cell suspensions of human samples were collected from tissues, blood, and co-cultured systems. The cells were stained with Fixable Viability Stain 510 (BD Horizon, Cat#564406, USA), anti-CD33 (BioLegend, Cat#983906, USA), anti-CD11b (BioLegend, Cat#982614,), anti-HLA-DR antibodies (BioLegend, Cat#327008), anti-CD14 (Elabscience, Cat#E-AB-F1209E, China), and anti-CD15 (Elabscience, Cat#E-AB-F1079Q) at 4 °C for 30 min. Mouse PBMCs, spleen cells, blood, and tumor tissue cells were collected and stained with anti-CD11b (BioLegend, Cat#101206) and anti-Gr-1 (BioLegend, Cat#108412) antibodies at 4 °C for 30 min. The BD Calibur Flow cytometer (BD Pharmingen, CA, USA) was used to measure the percentage of MDSCs followed by FlowJo software for analyzing the data.

### MDSC isolation and suppression

Human MDSCs were isolated from PBMCs collected from healthy donors using the EasySep™ Human CD33 Positive Selection Kit II (STEMCELL Technologies, Cat#17876, Canada), following the manufacturer’s protocol. Tumor cells were co-cultured with MDSCs in the presence or absence of a GM-CSF neutralizing antibody (0.1 μg/mL, NeutraKine, IL, USA). Human GM-CSF (50 ng/mL, PeproTech, Cat#300-03-5UG, NJ, USA) was added as a stimulator for MDSCs. The levels of MDSCs were determined and were subsequently co-cultured with CD3^+^ T cells, which were isolated using the Human CD3 T Cell Isolation Kit (BioLegend, Cat#480131), and activated with the human CD3/CD28 T cell activator (STEMCELL Technologies, Cat#10991). After 72 h, the proliferation and activity of T cells (CD3^+^, CD4^+^, and CD8^+^) (BioLegend, Cat#981008, 980812, 980902) were measured by FACS analysis for quantification of CFSE (BioLegend, Cat#423801), IFN-γ (Invitrogen, Cat#69-7319-42), and TNF-α (eBioscience, Cat#11-7349-81), respectively. To isolate MDSCs from mouse tumor tissues, CD11b⁺Gr-1⁺ MDSCs were sorted using a MoFlo Astrios EQ flow cytometer (Beckman Coulter, CA, USA). Following 72 h of stimulation with Mouse T Cell Activator Kit (STEMCELL Technologies, Cat#100-1572), CD4⁺ (BioLegend, Cat#100408) and CD8⁺ T cell (BioLegend, Cat#100734) proliferation and effector function were analyzed by flow cytometry using CFSE dilution and intracellular IFN-γ (BioLegend, Cat#505829) and TNF-α (Elabscience, Cat#AN00567M) staining. For combinational therapy evaluation, Nivolumab (anti-PD-1, Selleck, Cat#A2002) and SN52 (RelB inhibitor, Selleck, Cat#P1238) were purchased from Selleck. Isolated MDSCs were co-cultured with tumor cells to assess MDSC proliferation in the presence of PD-1 antibody (5 nM) and/or SN52 (100 µg/mL). In parallel, tumor cells were co-cultured with PBMCs to evaluate the proliferation of CD3^+^, CD4^+^, and CD8^+^ T cells under the same conditions.

### Chromatin immunoprecipitation (ChIP)

The binding of RelB to the NF-κB elements present in the promoter region of the human *CSF2* gene was determined by ChIP assay using the ChIP-IT Express kit (Active Motif, Cat#53009, CA, USA). Chromatin was pulled down from PC-3 cells by applying RelB antibody (CST, Cat#D7D7W) or IgG (CST, Cat#3900S) antibody, which were considered the experimental group and negative group, respectively. Chromatin that was not subjected to any antibody treatment was regarded as input control. The fragments pulled down were analyzed by agarose gel electrophoresis and RT-qPCR using specific primers (Table S2). The fragments pulled down were quantified by band intensity or percent input for each ChIP fraction.

### Reverse transcription quantitative PCR (RT-qPCR)

Total cellular RNA was extracted using TRIzol reagent and then reverse transcribed (PrimeScript™ RT reagent kit, Takara Inc., Japan). Quantitative PCR was conducted with LightCycle System (Roche, Switzerland) using SYBR Premix Ex Taq II kit (Takara, Cat#RR820A). Levels of target genes were normalized to GAPDH. Sequences of PCR primers for the cytokines were listed in Table S2.

### Western blotting

Cell lysis was performed using RIPA buffer supplemented with 1 mM PMSF, then quantified by a BCA assay kit (Thermo Fischer, Cat#23227). Primary antibodies used in this study were as follows: β-actin (Proteintech, Cat#20536-1-AP, China), GAPDH (Proteintech, Cat#10494-1-AP), PCNA (Proteintech, Cat#10205-2-AP), ILF2 (Santa Cruze Biotech, Cat#sc-271718), RelB (CST, Cat#D7D7W), GM-CSF (Santa Cruze Biotech, Cat#sc-398649), STAT3 (CST, Cat#9139), p-STAT3 (CST, Cat#9145). HRP-conjugated secondary antibody was obtained from Santa Cruze Biotech. The immunoblots were visualized using an enhanced chemiluminescence detection system (Bio-Rad, CA, USA).

### Co-immunoprecipitation (Co-IP)

The endogenous Co-IP experiment was carried out using a Co-IP assay kit following the manufacturer’s instructions (Proteintech, Cat#PK10007). Briefly, PCa cells were lysed in IP lysis buffer containing complete protease inhibitor cocktails (Thermo Fischer, Cat#78429). Anti-RelB antibody (CST, Cat#D7D7W) was incubated with 500 μg of total protein extracts overnight at 4 °C, while an isotype anti-IgG antibody (CST, Cat#3900S) was used as a negative control. Protein A/G beads (Biyuntian Biotechnology, Cat#P2179S, China) were added for further incubation at 4 °C for 5 h, and the precipitates were separated on an SDS-PAGE for protein detection, including RelB, ILF2, and ubiquitination. For exogenous Co-IP performance, plasmids of Flag-RelB (wild type or mutant type) and GST-ILF2 were co-transfected into PC-3 cells. RelB was pulled down using anti-Flag antibody. ILF2 was detected by an anti-GST antibody. For the nuclear and cytoplasmic Co-IP experiment, nuclear and cytoplasmic protein extracts were isolated using the Nuclear and Cytoplasmic Extraction Kit (Thermo Fisher, Cat#78835).

### LC-MS/MS

After conducting an endogenous immunoprecipitation assay, the resulting IgG and IP (anti-RelB) protein samples were analyzed using LC-MS/MS.

### Pull-down assay

Briefly, we performed an extracellular pull-down assay to examine the interaction between different types of Flag-tagged RelB proteins (wild and mutant types) and recombinant GST-ILF2 protein. Flag-tagged RelB protein was immobilized on anti-Flag magnetic beads and incubated with 5 μg of recombinant GST-ILF2 protein (Abcam, Cat#ab132371) at 4 °C for 24 h. The Flag-tagged complexes were pulled down for GST-ILF2 protein detection by western blotting analysis.

### Elisa

The secreted concentration of GM-CSF was determined by ELISA assay (Elabscience, Cat#E-EL-H0081) according to the manufacturer’s instructions.

### Immunofluorescence

PCa cells were fixed using 100% ice-cold methanol for 15 min and permeabilized with 0.1% Triton for 15 min. Then the cells were blocked with 5% BSA and incubated overnight at 4 °C with primary antibodies against ILF2 and RelB. Subsequently, cells were stained with fluorescent-conjugated anti-mouse AF647(Abcam, Cat#ab150115) and anti-rabbit AF488 (Abcam, Cat#ab150077) second antibodies. The nucleus was stained with DAPI. Fluorescence was visualized and captured using a confocal microscope (LSM800, Germany). For tissue determination, mouse xenograft tumor tissue sections were subjected to immunofluorescent staining for RelB, ILF2, and GM-CSF, as well as the tumor infiltration of MDSCs (CD11b/Gr-1) and T cells (CD4 and CD8). The nucleus was stained with DAPI (Invitrogen, Cat#D1306).

### Protein stability and degradation assays

To investigate the mechanism underlying ILF2-mediated regulation of RelB stability, PC-3 cells with stable ILF2 knockdown were treated with the proteasome inhibitor MG132 (Selleck, Cat#S2619, USA) or the lysosomal inhibitor bafilomycin A1 (Baf A1, Selleck, Cat#S1413). Cells were incubated with MG132 (10 μM) or Baf A1 (100 nM) for 6 h prior to harvest. RelB protein levels in whole-cell lysates, cytoplasmic fractions, and nuclear fractions were subsequently analyzed by western blotting.

For cycloheximide (CHX) chase assays, control and shILF2 PC-3 cells with or without MG132 treatment were incubated with CHX (MedChemExpress, Cat#HY-12320, USA) at a final concentration of 50 μg/mL to inhibit de novo protein synthesis. Cells were collected at 0, 2, 4, and 8 h after treatment, and nuclear protein extracts were prepared for western blotting. RelB protein abundance was quantified by densitometric analysis, normalized to PCNA, and expressed relative to the corresponding 0 h group.

### Ubiquitination assay

For ubiquitination assays, cells were treated with MG132 (10 μM) for 6 h prior to harvest. Cells were lysed in denaturing lysis buffer containing 2% SDS, 150 mM NaCl, and 10 mM Tris-HCl (pH 8.0) supplemented with protease inhibitor cocktail, followed by boiling for 10 min and sonication. Lysates were subsequently diluted and approximately 1000 μg of total protein was subjected to immunoprecipitation overnight at 4 °C with anti-RelB antibody (CST, Cat#D7D7W), followed by incubation with Protein A/G magnetic beads (BeyoMag, Cat#P2108, China) for 2 h at 4 °C. Beads were collected using a magnetic separator, washed three times with 1× TBS, and eluted in 1× SDS loading buffer at 95 °C for 5 min prior to immunoblotting. Ubiquitinated RelB was detected by western blotting using anti-ubiquitin (Santa Cruz Biotechnology, Cat#sc-53509) or K48-linked ubiquitin antibody (CST, Cat#4289). Immunoprecipitated RelB was analyzed in parallel to confirm immunoprecipitation efficiency. Equal input loading was confirmed by GAPDH immunoblotting. Ubiquitinated RelB signals were quantified by densitometric analysis, normalized to immunoprecipitated RelB levels.

### Mice

Male C57BL/6 mice (6–8 weeks old) were used for all in vivo experiments. Mice were allocated to tumor cell implantation or treatment groups by simple randomization before tumor implantation. Investigators were not blinded to group allocation or outcome assessment. C57BL/6 mice were randomly divided into five groups (*n* = 6) followed by subcutaneously injected with 2 × 10^6^ RM-1 control, shILF2, shILF2/RelB^+^, RelB-KO, and RelB-KO/GM-CSF^+^ cells, respectively. Tumor growth was monitored every 2 days. Mice were sacrificed 15 days post injection for sample collection. Tumor-infiltrating MDSCs (CD11b⁺ Gr-1⁺) were sorted via flow cytometry and co-cultured with T cells to validate the immunosuppressive function of MDSCs. To evaluate targeted therapy, C57BL/6 mice were randomly assigned to six groups (*n* = 6). In the PD-1 group, mice received intraperitoneal injections of PD-1 antibody (200 μg, Selleck, Cat#A2122) every 2 days for a total of four doses, starting on the day of tumor implantation. In the SN52 group, SN52 (40 μg) was administered intratumorally every 2 days for four doses. The infiltration of MDSCs and CD8⁺ T cells was analyzed by flow cytometry.

### Statistics

No formal statistical methods were used to predetermine sample size. Sample sizes were chosen based on prior experience with similar experiments and established practices in the field. No data were excluded from the analyzes unless they failed prespecified quality-control criteria. All experiments were performed with a minimum of three independent replicates. Data were presented as the mean ± standard deviation (s.d.). Data distribution and variance were assessed prior to statistical analysis to ensure that the assumptions of the applied statistical tests were reasonably satisfied. Statistical analyzes were carried out using GraphPad Prism software (GraphPad, San Diego, CA, USA). Differences between two independent groups were evaluated using a two-tailed unpaired Student’s *t*-test. Comparisons among multiple groups were performed using one-way analysis of variance (ANOVA), followed by Dunnett’s or Bonferroni’s post hoc test as appropriate. Correlations were analyzed using Pearson’s correlation coefficient. The threshold for statistical significance was set at *P* < 0.05.

## Supplementary information

Supplementary Figures and tables-R1

Original WB Data Files

## Data Availability

The public single-cell RNA-sequence data used in this study were obtained from the GEO database (GSE141445). Other supporting data are available from the corresponding author upon reasonable request.

## Code availability

No custom code was developed for this study. All bioinformatic and statistical analyses were performed using publicly available software and resources as described in the Methods.
