# Supplementary material for: RelB/ILF2–GM-CSF loop sustains tumor–MDSC communication in prostate cancer immunosuppression
Source: Cell Death Dis. 2026 Jul 11;17(1):863. doi: 10.1038/s41419-026-09098-x (PMC13639098; doi:10.1038/s41419-026-09098-x)
Supplement: Supplementary file 1 [file 41419_2026_9098_MOESM1_ESM.pdf]

Figure S1

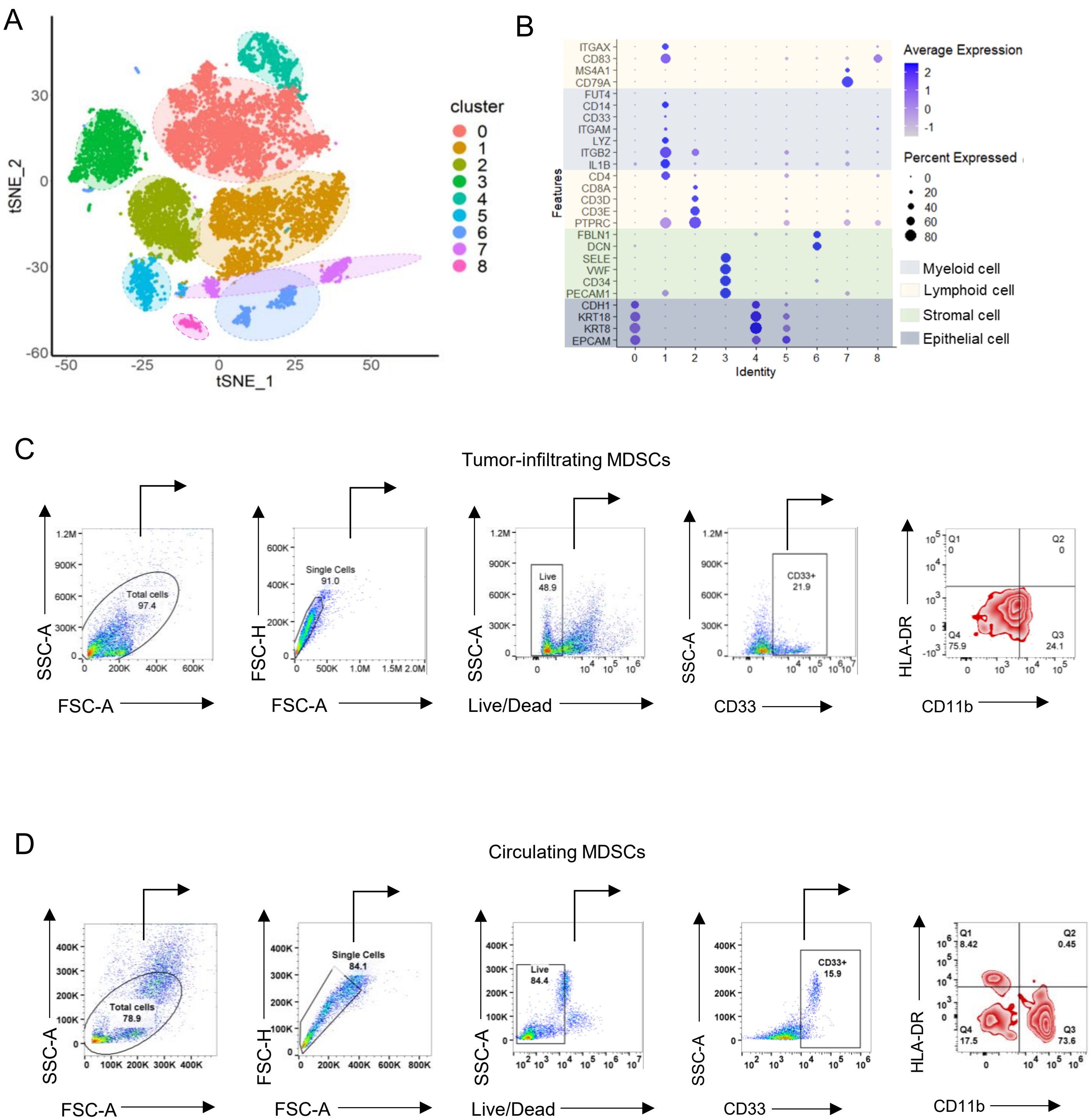

Fig. S1: MDSC identification in prostate cancer. (**A**) Nine cell type cluster distribution by Seurat in PCa and normal tissues. (**B**) Classification of cell clusters, including myeloid cells, lymphoid cells, stromal cells, and epithelial cells. (**C**) Gating strategy for tumor-infiltrating MDSCs in prostate cancer patients. (**D**) Gating strategy for peripheral blood MDSCs in prostate cancer patients.

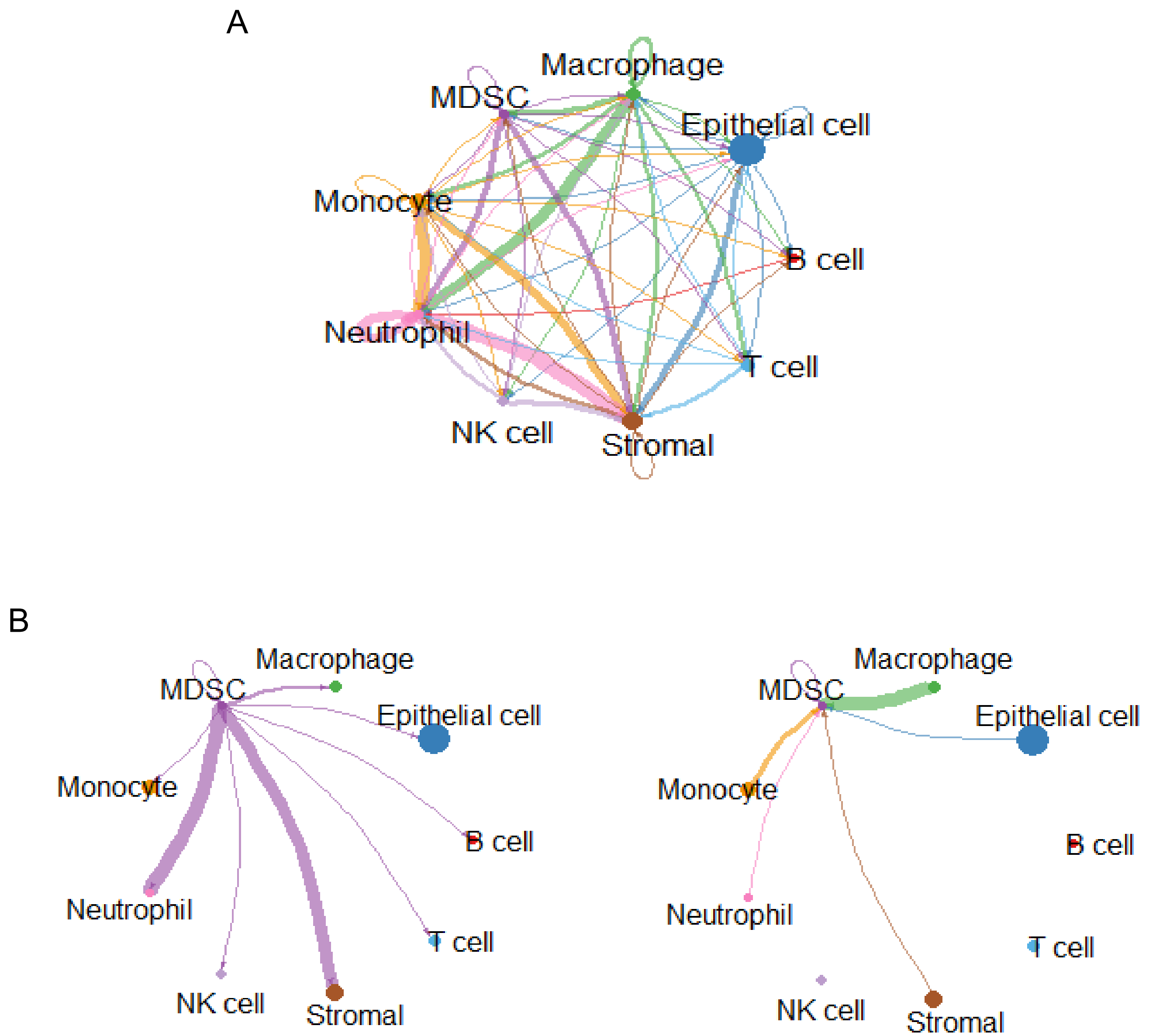

Fig. S2: Single-cell sequence reveals potential interactions of MDSCs with other cell types. (**A**) Cellular interplay among indicated cells. (**B**) The potential two-way communications between MDSCs and immune cells, stromal cells, and epithelial cells. Cell-cell interactions were inferred based on known ligand-receptor. Each node represents a cell type, with size indicating communication strength. Edges denote significant interactions, with width indicating strength and color indicating the sender cell type. The left panel shows signals sent from MDSCs, and the right panel shows signals received by MDSCs from other cells.

Figure S3

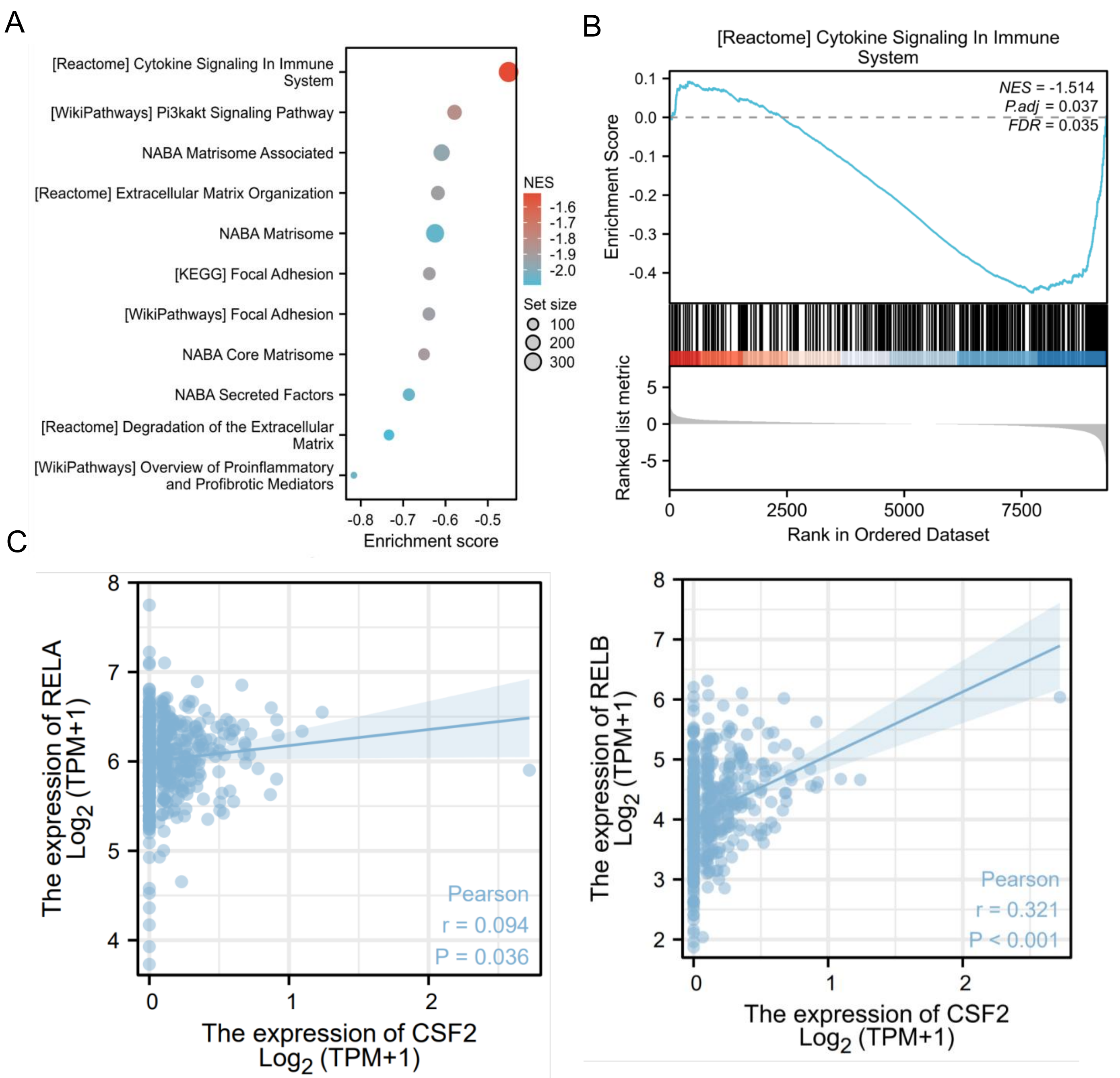

Fig. S3: RelB-mediated signaling pathway in PC-3 cells. (**A**) The signaling pathway mediated by RelB. (**B**) RelB positively regulated cytokine signaling pathways in immune system. (**C**) A linear regression analysis of correlation between RelB, RelA and GM-CSF in PCa.

Figure S4

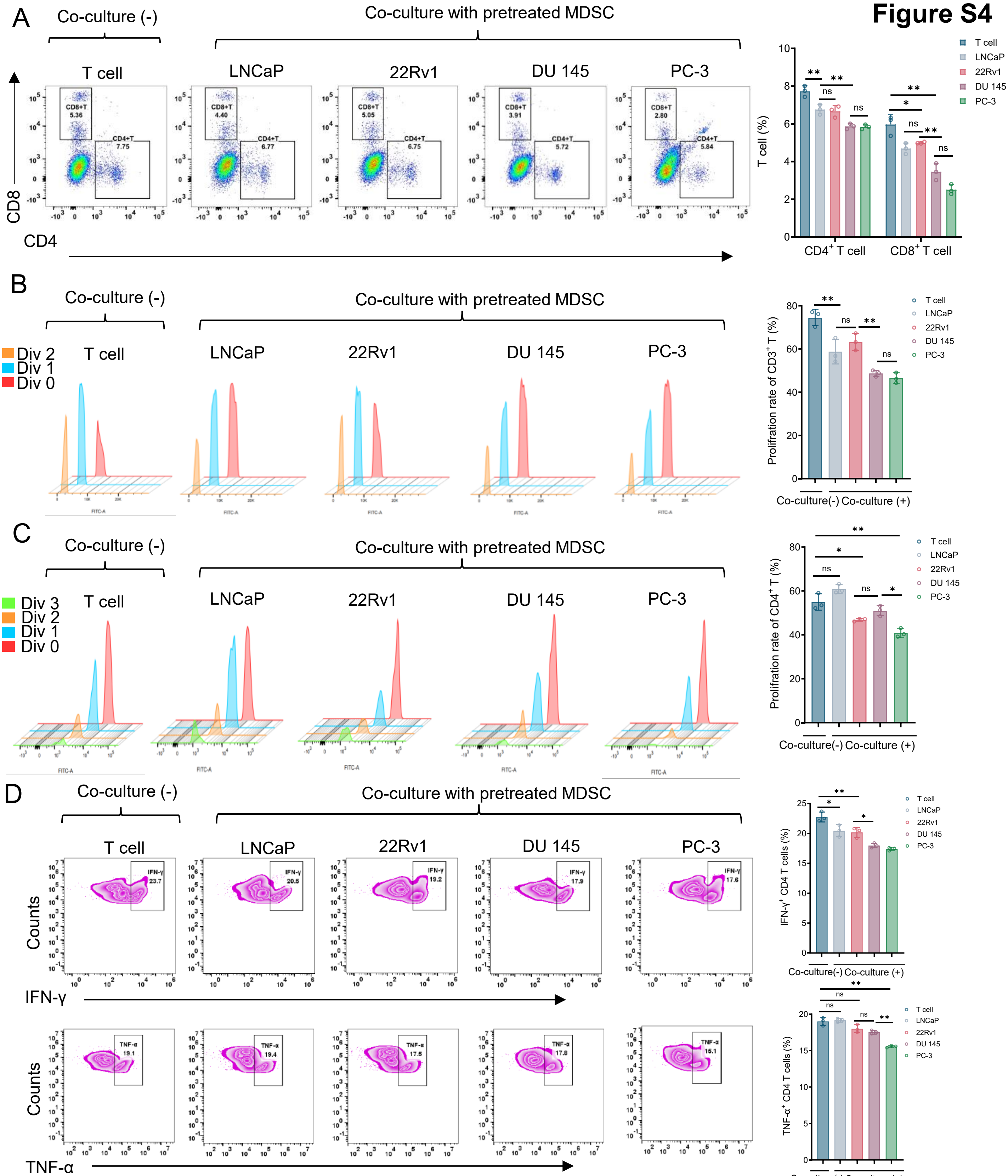

Fig. S4: MDSCs immunosuppressive function on T cell. (**A**) Representative image (left) and quantitation (right, n=3) of T cell abundance co-cultured with pretreated MDSCs. (**B**) Representative image (left) and quantitation (right, n=3) of CD3⁺ T cell. (**C**) Representative image (left) and quantitation (right, n=3) of CD4⁺ T cell. (**D**) Representative image (left) and quantitation (right, n=3) of IFN-γ⁺ and TNF-α⁺ CD4 T cells. Data are shown as the mean ± s.d., *$P < 0.05$, **$P < 0.01$; one-way ANOVA (A, B, C, D).

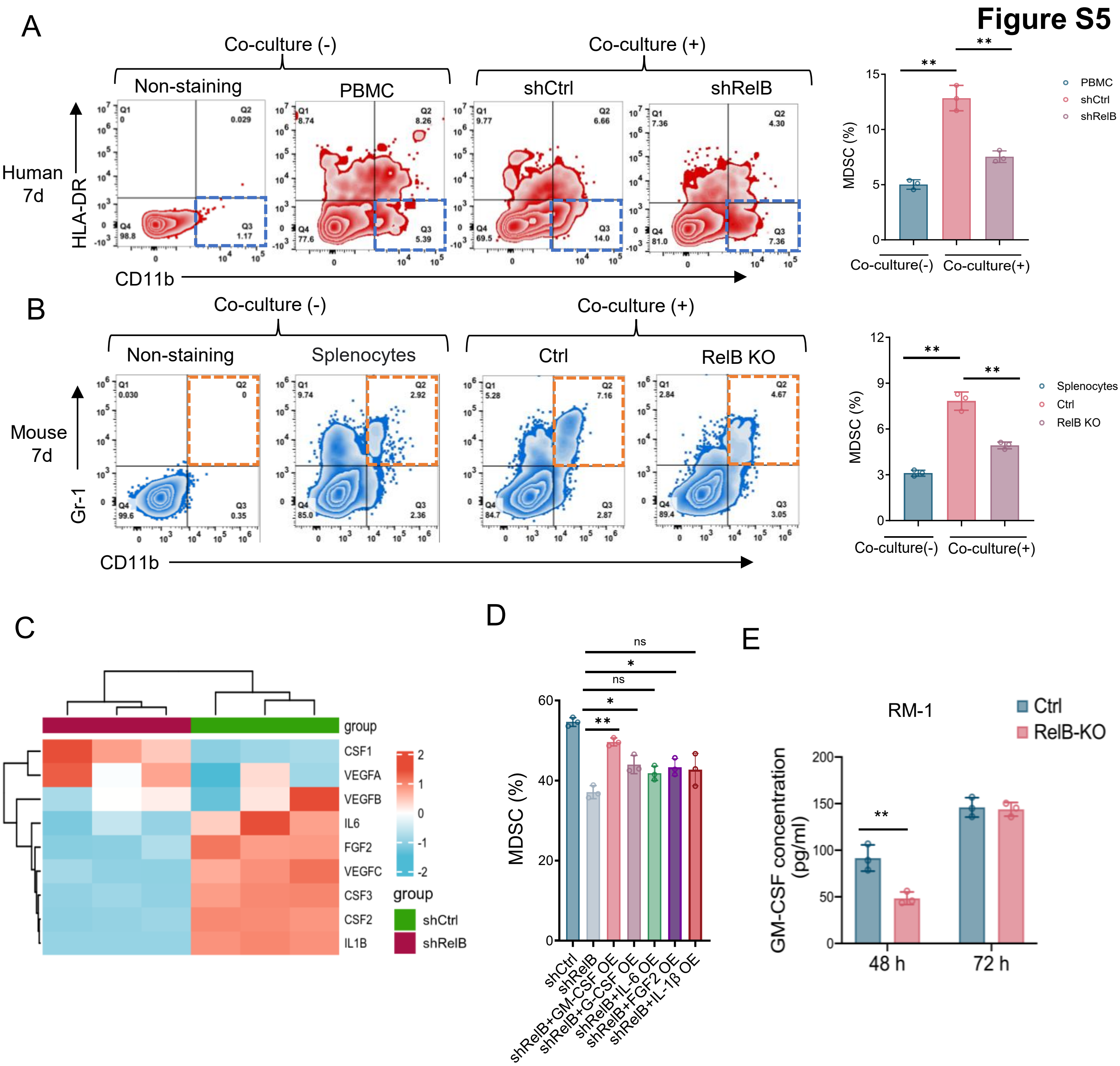

Fig. S5: MDSCs expansion regulated by RelB. (**A**) Representative image (left) and quantitation (right, n = 3) of MDSCs after co-culture of human PBMCs with PC-3 cells (wild-type or RelB knockdown) for seven days. (**B**) Representative image (left) and quantitation (right, n = 3) of MDSCs ($Gr\text{-}1^{+}CD11b^{+}$) after splenocytes and RM-1 cells (wild-type or RelB knockout) co-culture for seven days (**C**) The expression of cytokines associated with MDSC expansion measured by transcriptomes of RelB knockdown PC-3 cells. (**D**) Quantification of MDSC proportions in shCtrl, shRelB, and shRelB cells with indicated cytokine overexpression. (**E**) GM-CSF secretion in RM-1 cells under RelB inhibition for 48 and 72 h (n = 3). Data are shown as the mean ± s.d., **P* < 0.05, ***P* < 0.01; one-way ANOVA (A, B, D); Student's t-test (E).

Figure S6

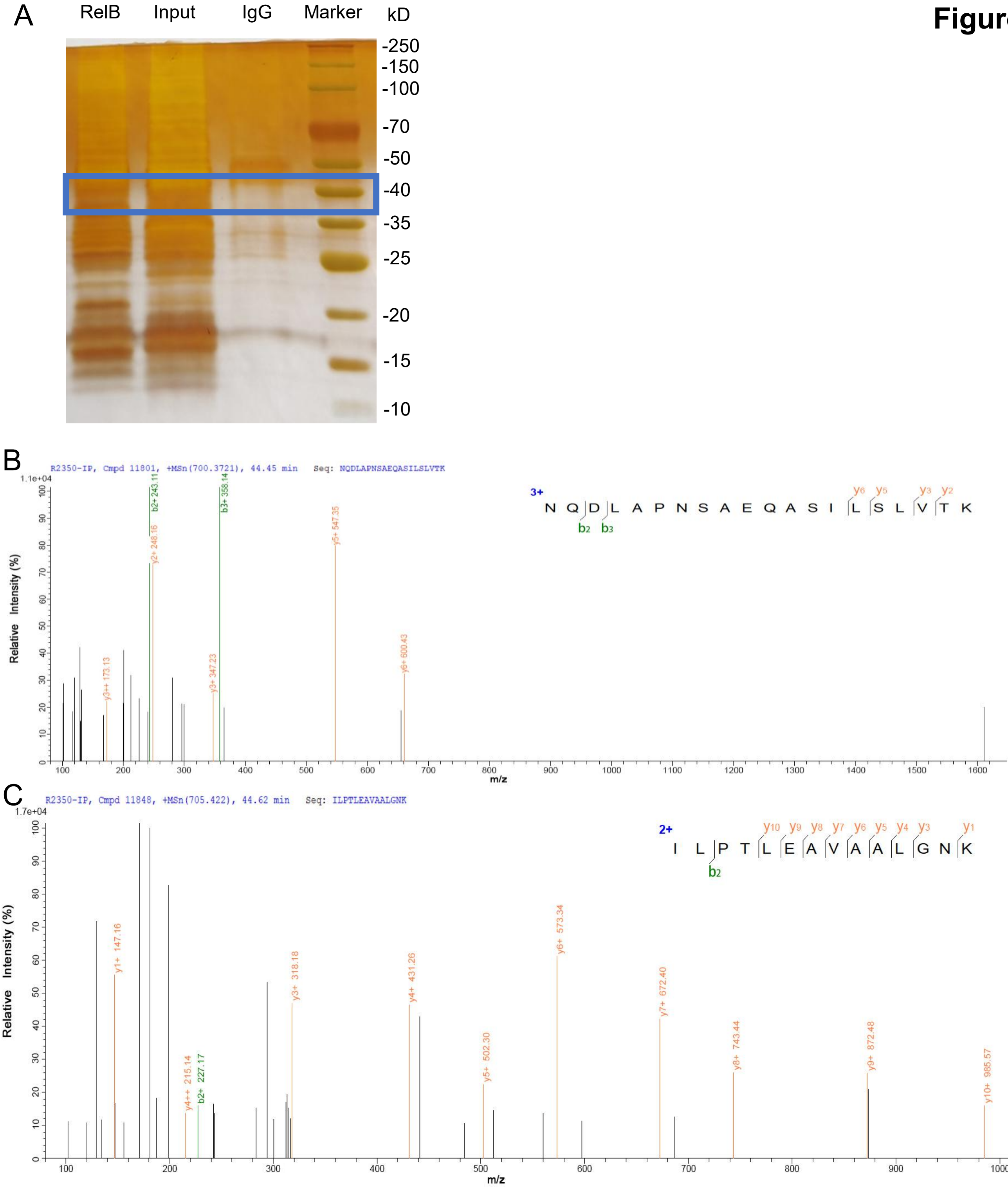

Fig. S6: Identification of interaction of ILF2 and RelB. (**A**) Silver staining of the protein complex containing RelB. (**B, C**) Representative MS/MS spectra of the tryptic peptides from ILF2: **(B)** NQDLAPNSAEQASILSLVTK and **(C)** ILPTLEAVAALGNK. The b and y ions were indicated with green and yellow colors, respectively.

Figure S7

A

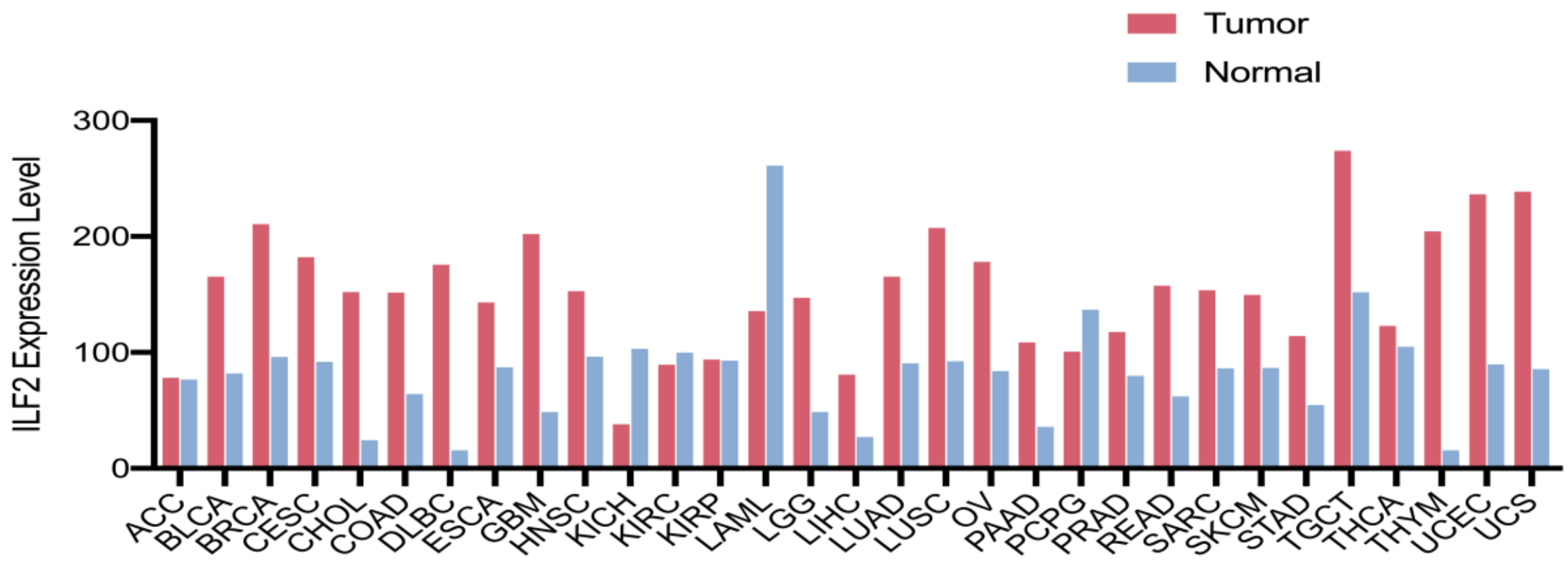

B

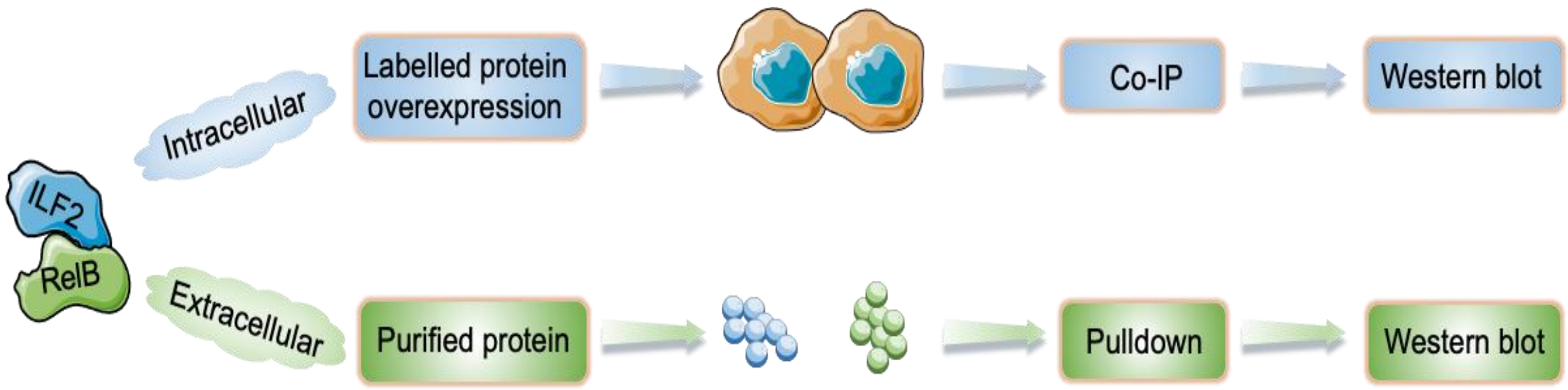

Fig. S7: Estimated interactions between ILF2 and RelB. (**A**) ILF2 expression among various cancer types analyzed by the GEPIA database. (**B**) The flow chart outlines the working procedure for testing intracellular Co-IP and extracellular Pulldown assays.

Figure S8

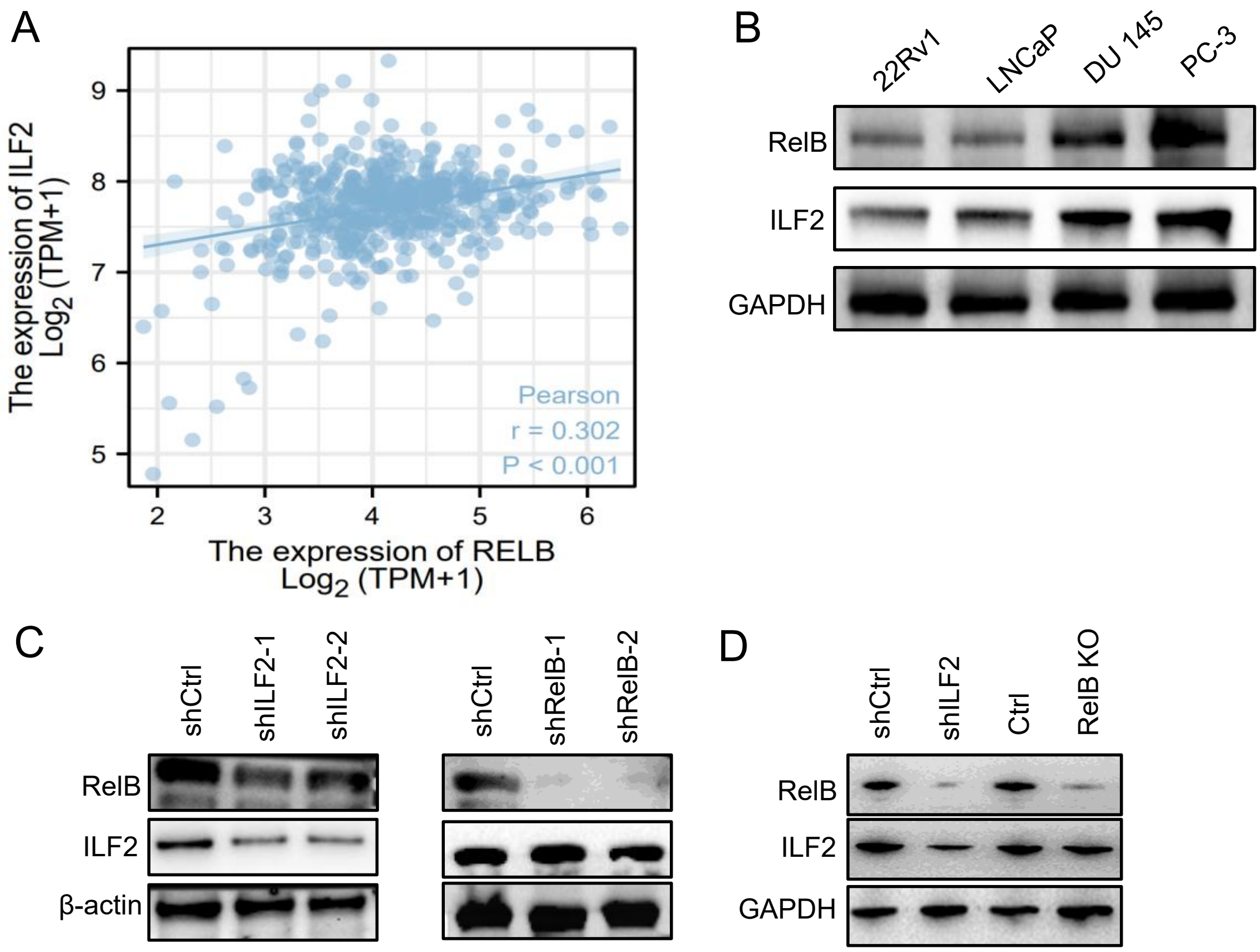

Fig. S8: ILF2 regulates RelB expression. (**A**) A linear regression analysis of RelB and ILF2 expression in PCa. (**B**) The expression of RelB and ILF2 in AR-dependent and AR-independent PCa cells. (**C, D**) ILF2 and RelB expression was manipulated and assessed both in PC-3 (**C**) and RM-1 cells (**D**).

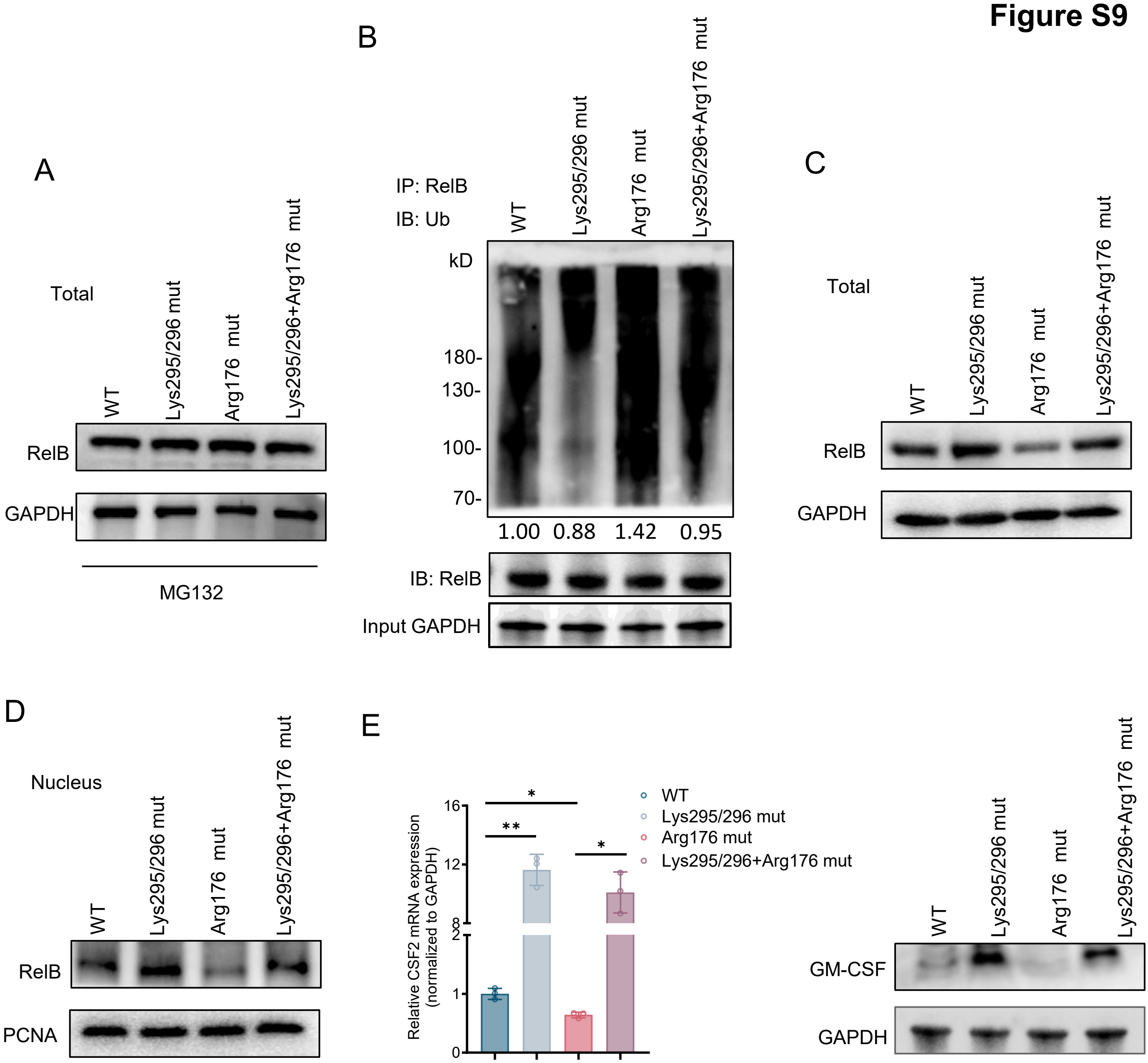

Fig. S9: Functional validation of RelB Arg176 and Lys295/296 residues. (A) Expression of RelB in PC-3 cells expressing wild-type (WT) or mutant RelB constructs (Lys295/296 mutant, Arg176 mutant, or double mutant) following depletion of endogenous RelB by 3′UTR-targeting shRNA. Cells were treated with MG132 before blotting. (**B**) Ubiquitination of RelB in PC-3 cells. Cells were treated with MG132 before IP using anti-RelB antibody, followed by immunoblotting (IB) for ubiquitin (Ub). (**C**) CHX chase assay of RelB stability at 6 h. (**D**) RelB nuclear localization in the indicated groups. (**E**) Relative GM-CSF mRNA and protein expression normalized to GAPDH. Data are shown as the mean ± s.d., **P* < 0.05, ***P* < 0.01; one-way ANOVA (E).

Figure S10

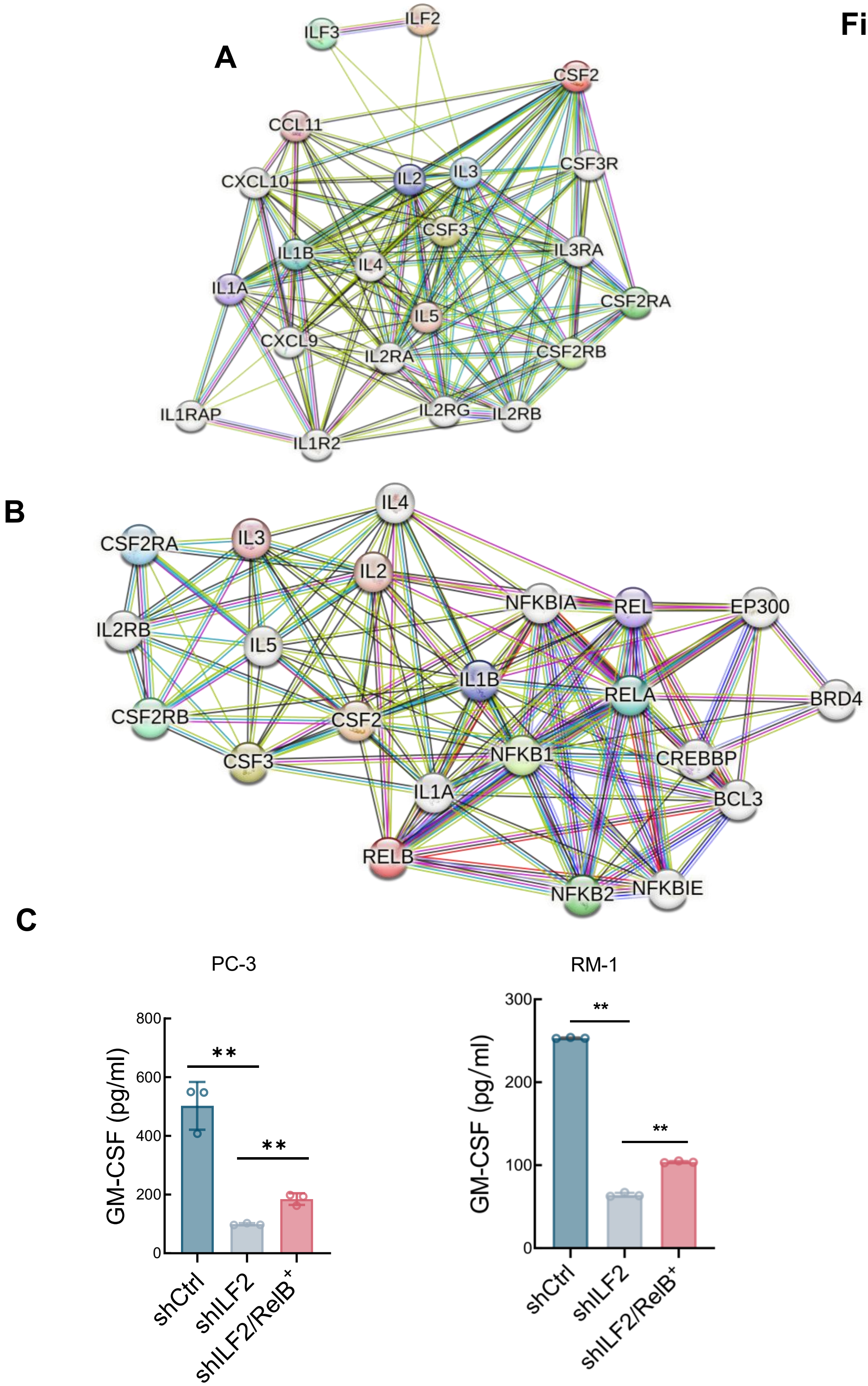

Fig. S10: The effect of RelB/ILF2 on GM-CSF-mediated MDSCs expansion. (A) Protein-Protein Interaction Network among indicated proteins. RelB appeared to modulate *CSF2* directly. (**B**) Contrarily, no mutual interaction was found between ILF2 and *CSF2.* (**C**) GM-CSF concentration in corresponding PCa cells by ELISA assay (n=3). Data are shown as the mean ± s.d., *P < 0.05, **P < 0.01; one-way ANOVA (C).

Figure S11

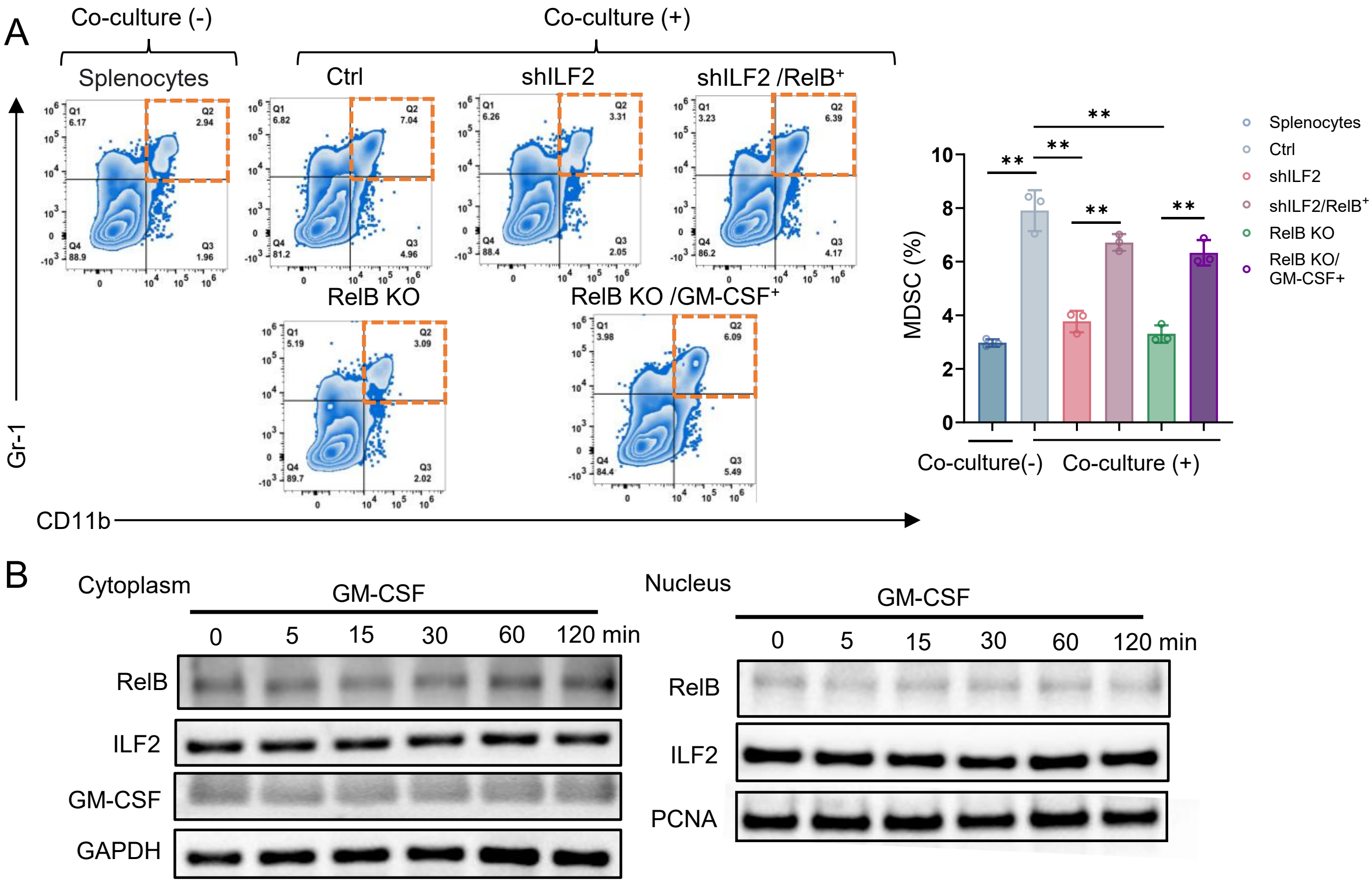

Fig. S11: The GM-CSF-RelB/ILF2 feedback loop mediated MDSCs expansion. (**A**) Representative image (left) and quantitation (right, n = 3) of MDSC level after incubation of splenic suspension and RM-1 cells as indicated. (**B**) The expression level of RelB, ILF2, and GM-CSF was assessed in the cytoplasm and nucleus of PC-3 cells with RelB knockdown (shRelB) under GM-CSF induction for the designated time intervals. Data are shown as the mean ± s.d., $^{*}P < 0.05$, $^{**}P < 0.01$; one-way ANOVA (A).

Figure S12

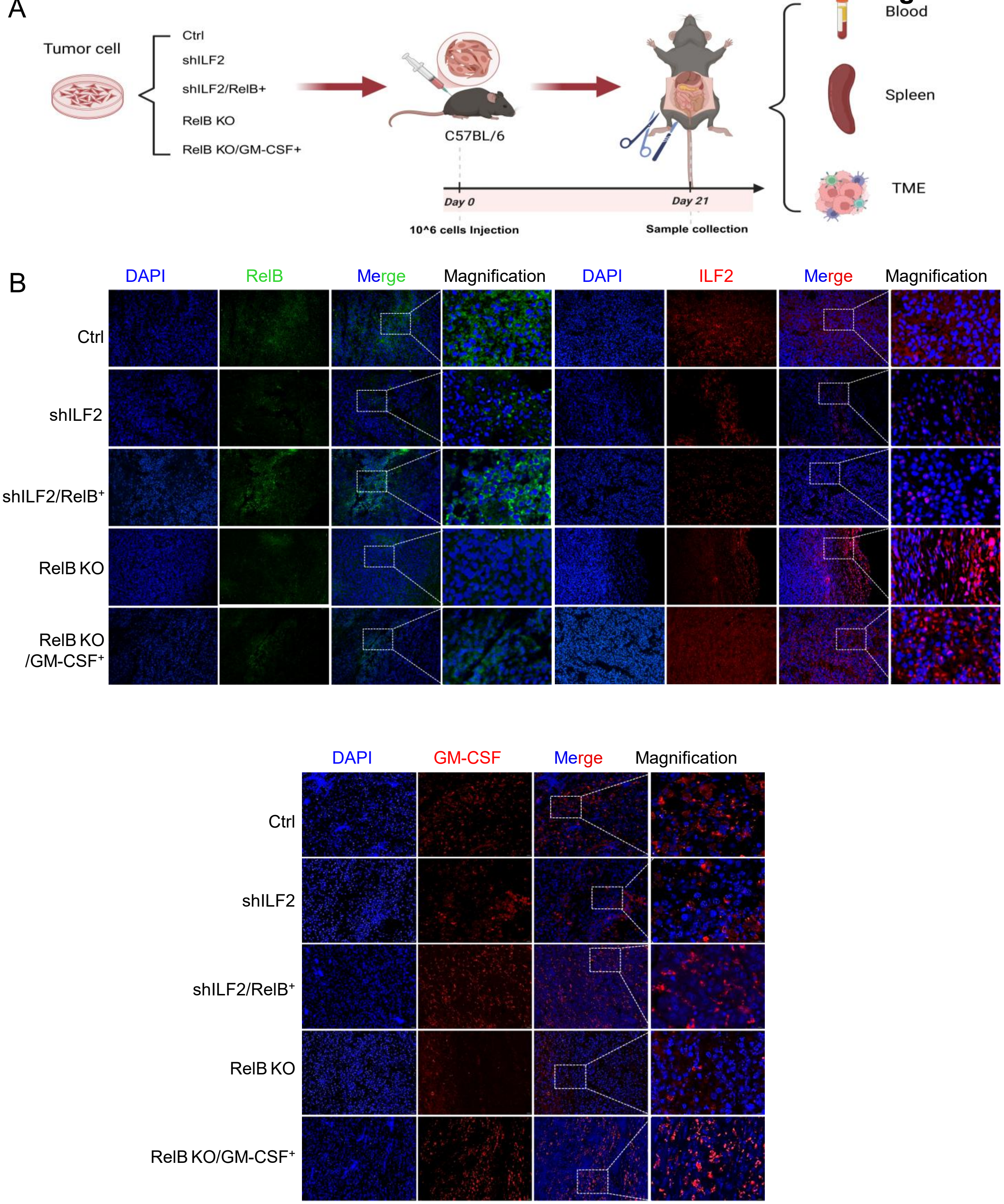

Fig. S12: Validation of protein expression in mouse tumors. . (**A**) Schematic grouping for in vivo tests. (**B**) The expression of RelB (green), ILF2 (upper panel, red), and GM-CSF (lower panel, red) was visualized by immunofluorescence in the indicated groups. The cell nucleus was stained with DAPI (blue).

Figure S13

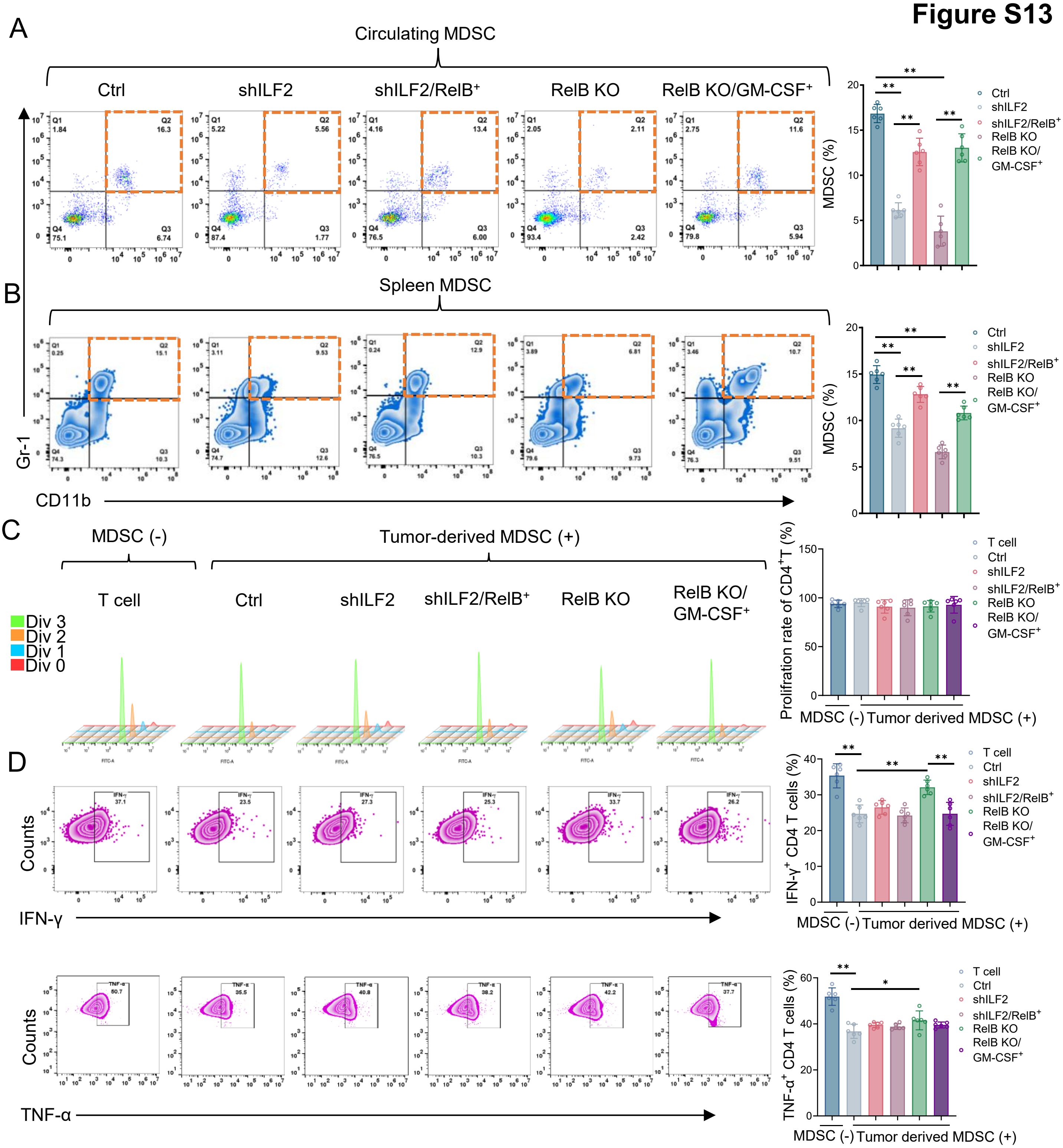

Fig. S13: ILF2/RelB mediated MDSCs expansion in mice. (**A**) Representative image (left) and quantitation (right, n=6) of MDSCs abundance in the circulating system of mice in the indicated groups. (**B**) Representative image (left) and quantitation (right, n = 6) of MDSCs level quantification in the spleen of mice in the indicated groups. (**C, D**) Representative image (left) and quantitation (right, n = 6) of CFSE-positive (C) and IFN-γ, TNF-α-positive CD4⁺ T cell (**D**) as indicated. Data are shown as the mean ± s.d., *$P < 0.05$, **$P < 0.01$; one-way ANOVA (A, B, C, D).

Figure S14

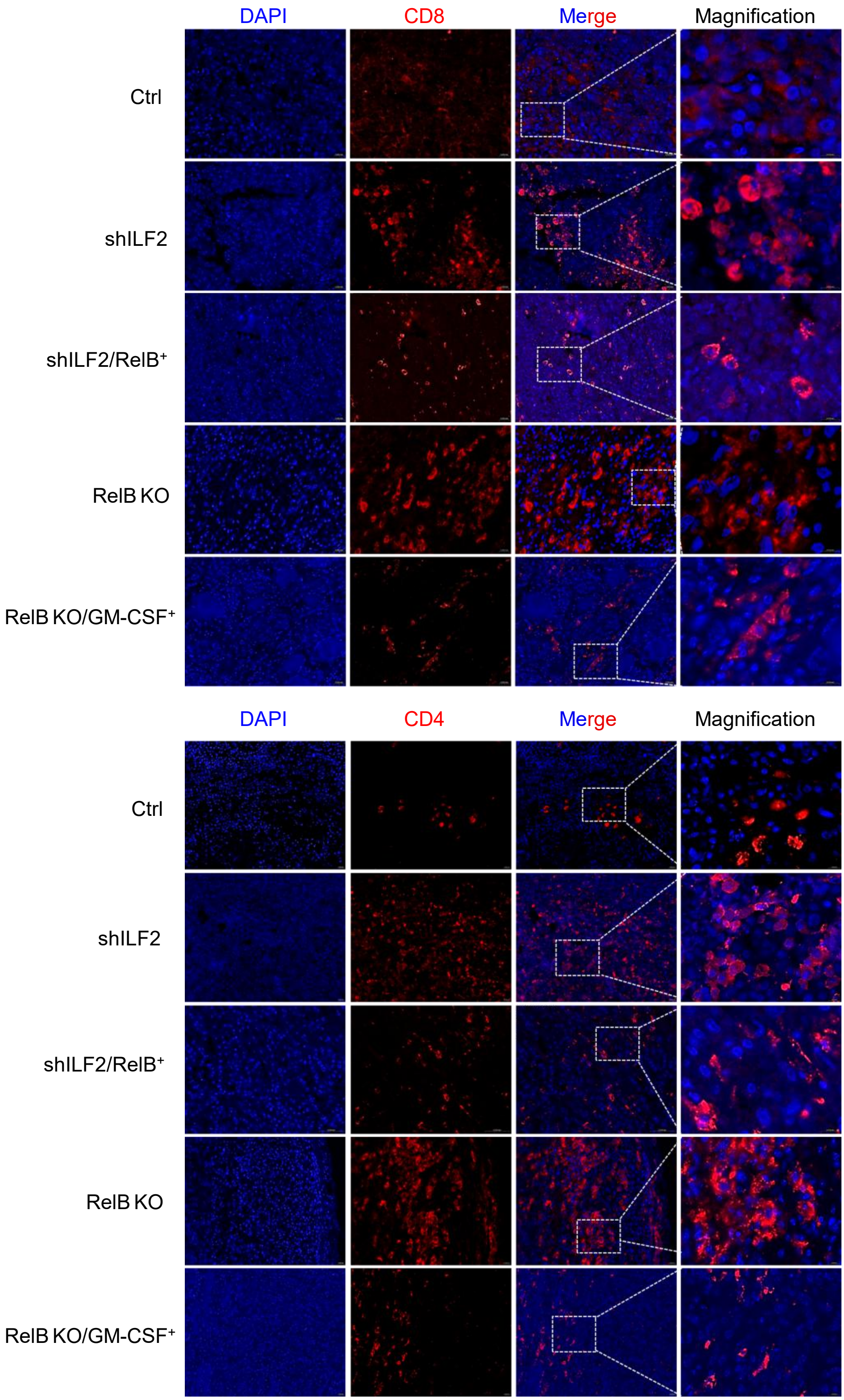

Fig. S14: T cell infiltration in mouse tumors. The infiltration of $CD8^+$ (upper panel) and $CD4^+$ T cell (lower panel) was visualized by immunofluorescence microscope in the specified groups. The cell nucleus was stained with DAPI (blue).

Figure S15

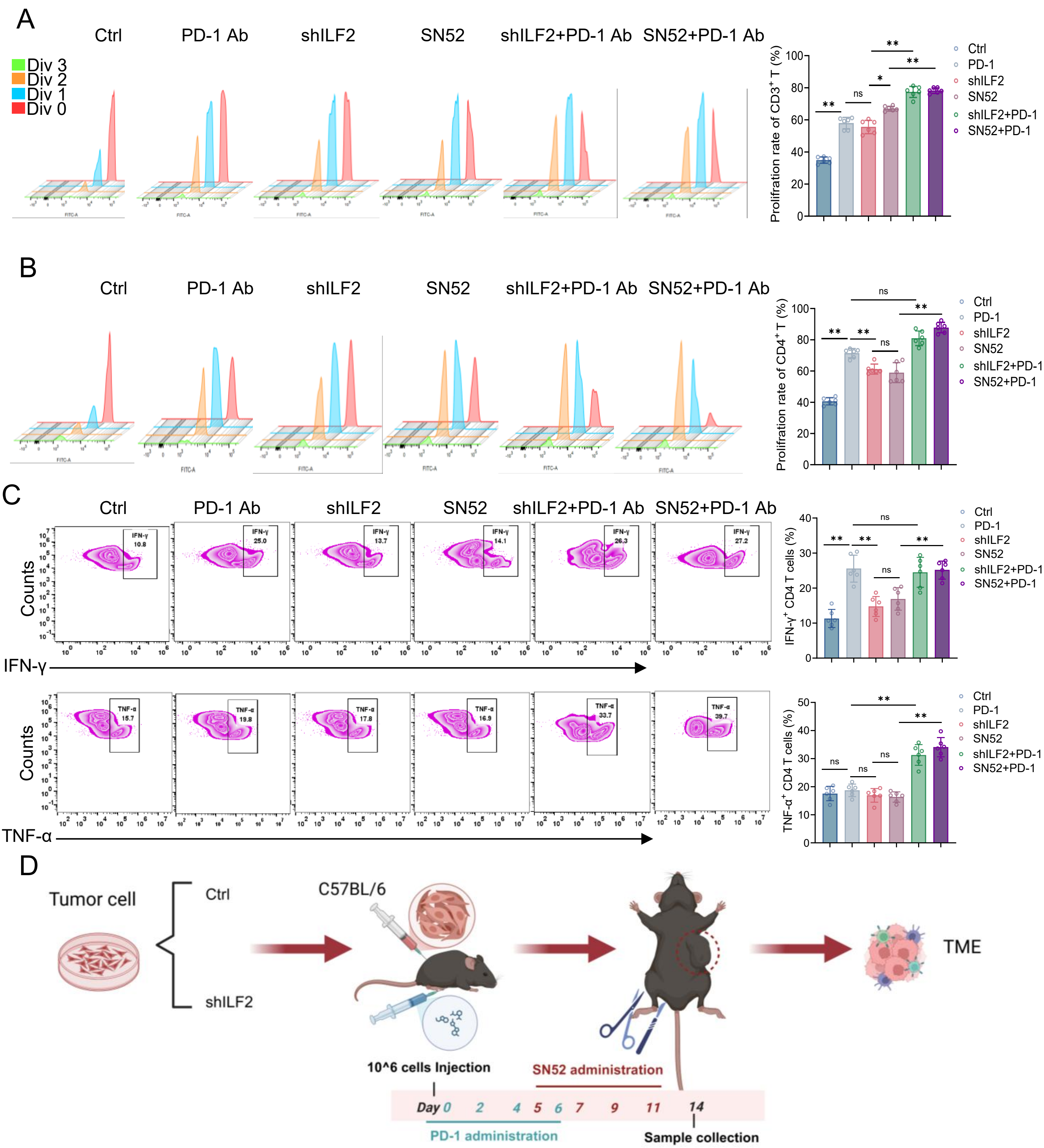

Fig. S15: Targeting RelB enhances antitumor effect of ICB. (**A, B**) Representative image (left) and quantitation (right, n = 6) of $CD3^+$ T cell (**A**) and $CD4^+$ T cell (**B**) proliferation determination by co-culture of prostate cancer cells with $CD33^+$ cells in the presence of PD-1 antibody and/or SN52. (**C**) Representative image (left) and quantitation (right, n = 6) of IFN-γ, TNF-α-positive $CD4^+$ T cell. (**D**) A schematic diagram of the drug administration and operation of the mice. Data are shown as the mean ± s.d., $*P < 0.05$, $**P < 0.01$; one-way ANOVA (A, B, C).

Table S1. **Identification of protein interacted with RelB**

Gene names	Peptides-IGG	Peptides-IP	Sequence coverage [%]	Mol. Weight (kDa)
RPS5	2	3	30.6	25.0875
RPS3	5	6	28.4	27.0945
RPL7	1	2	8.9	27.5405
RPL8	1	2	13.2	28.6555
RPS4X	2	4	16	29.3245
RPS3A	1	3	20.7	29.436
ALYREF	1	2	12.1	29.436
PRPS1	0	3	15.4	31.7775
CAPZA1	1	5	28	31.889
HNRNPC	5	6	19.7	32.335
NPM1	2	3	15.8	32.781
SLC25A5	5	6	18.5	33.227
LRRC59	1	2	7.2	34.2305
HNRNPA1	4	6	26.7	34.2305
RPLP0	1	3	24.8	35.3455
ELAVL1	2	4	18.4	36.349
GAPDH	3	5	27.7	37.3525
DKFZp686E1899	1	2	8.8	39.248
HNRNPA2B1	5	6	21.2	39.3595
PRPSAP1	0	2	7.3	39.694
H2AFY	3	5	19.1	41.478
YBX1	2	4	27.8	41.701
D10S102	1	2	9.3	42.147
ILF2	1	2	29.6	43.485

Table S2. Sequences of RT-qPCR/ChIP primers.

RT-qPCR primer		Sequence
M-CSF		Forward: 5'-AGCATCTTCTCCACTCCCTG-3'
		Reverse: 5'-GATGAGATCCTGGGAGAGCC-3'
VEGFA		Forward: 5'-GGCCAGCACATAGGAGAGAT-3'
		Reverse: 5'-ACGCTCCAGGACTTATACCG-3'
IL6		Forward: 5'-AGTCCTGATCCAGTTCCTGC-3'
		Reverse: 5'-CTACATTTGCCGAAGAGCCC-3'
FGF2		Forward: 5'-AGGAGTGTGTGCTAACCGTT-3'
		Reverse: 5'-CAGTTCGTTTCAGTGCCACA-3'
G-CSF		Forward: 5'-GGAGAAGCTGGTGAGTGAGT-3'
		Reverse: 5'-CAGCTGCAGTGTGTCCAAG-3'
IL1B		Forward: 5'-CGAATCTCCGACCACCACTA-3'
		Reverse: 5'-AGCCTCGTTATCCCATGTGT-3'
GM-CSF		Forward: 5'-GCGTCTCCTGAACCTGAGTA-3'
		Reverse: 5'-TGGGTTGCACAGGAAGTTTC-3'
GAPDH		Forward: 5'- TCTGACTTCAACAGCGACACC-3'
		Reverse: 5'- CTGTTGCTGTAGCCAAATTCGTT-3
**ChIP primer**	**Location**	**Sequence**
BS1	-100	Forward: 5'-ACCTAGGGAAAAGGCTCACC-3'
		Reverse: 5'-ATTAATGGTGACCACAAAATGC-3'
BS2	-1994	Forward: 5'-TTGGATCCCCAAGCTAGAGA-3'
		Reverse: 5'-AGGAAACAGACTGCAATGGA-3'
