# Supplementary material for: RelB/ILF2–GM-CSF loop sustains tumor–MDSC communication in prostate cancer immunosuppression
Source: Cell Death Dis. 2026 Jul 11;17(1):863. doi: 10.1038/s41419-026-09098-x (PMC13639098; doi:10.1038/s41419-026-09098-x)

Figure 2B

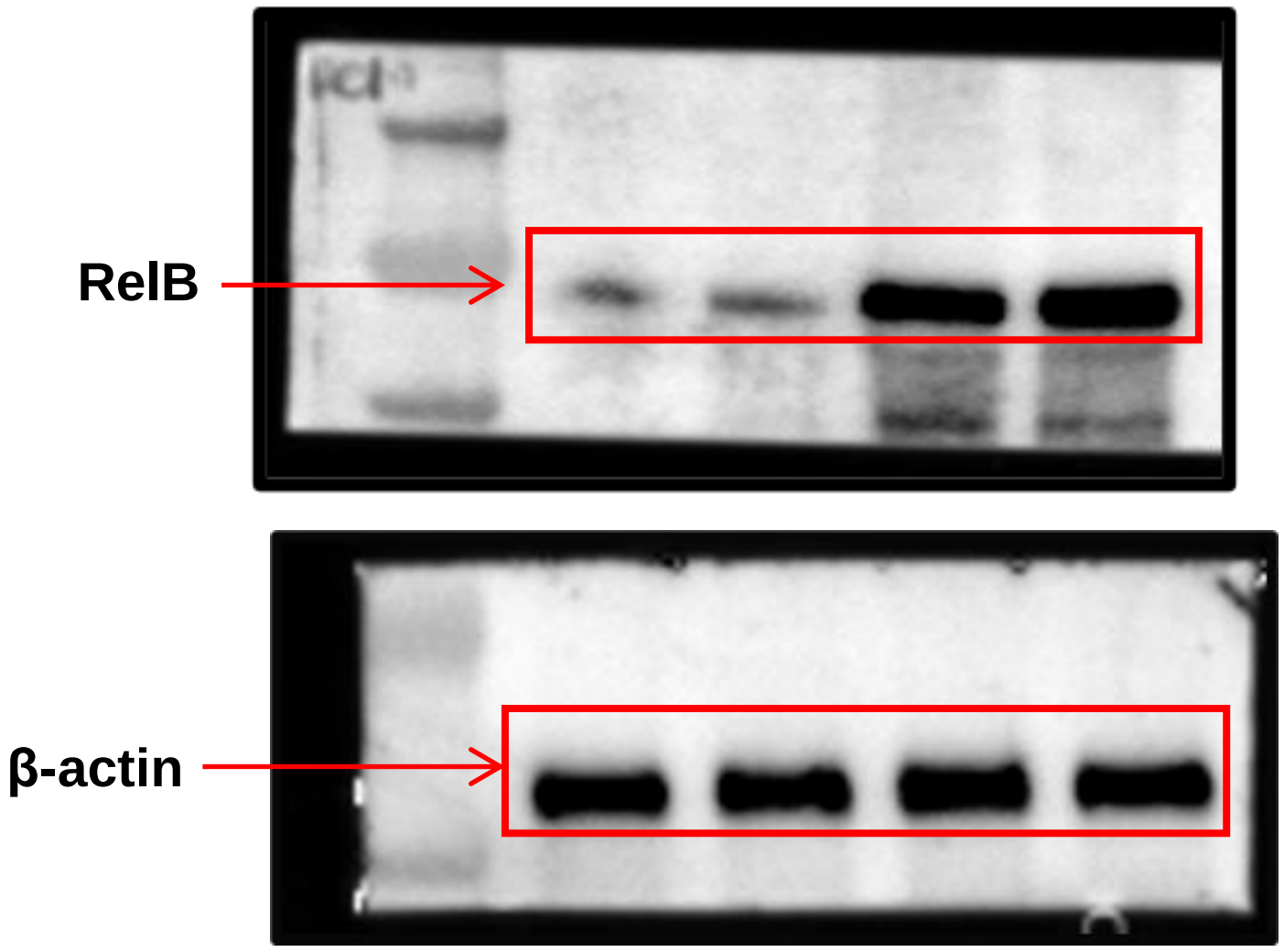

Figure 4D

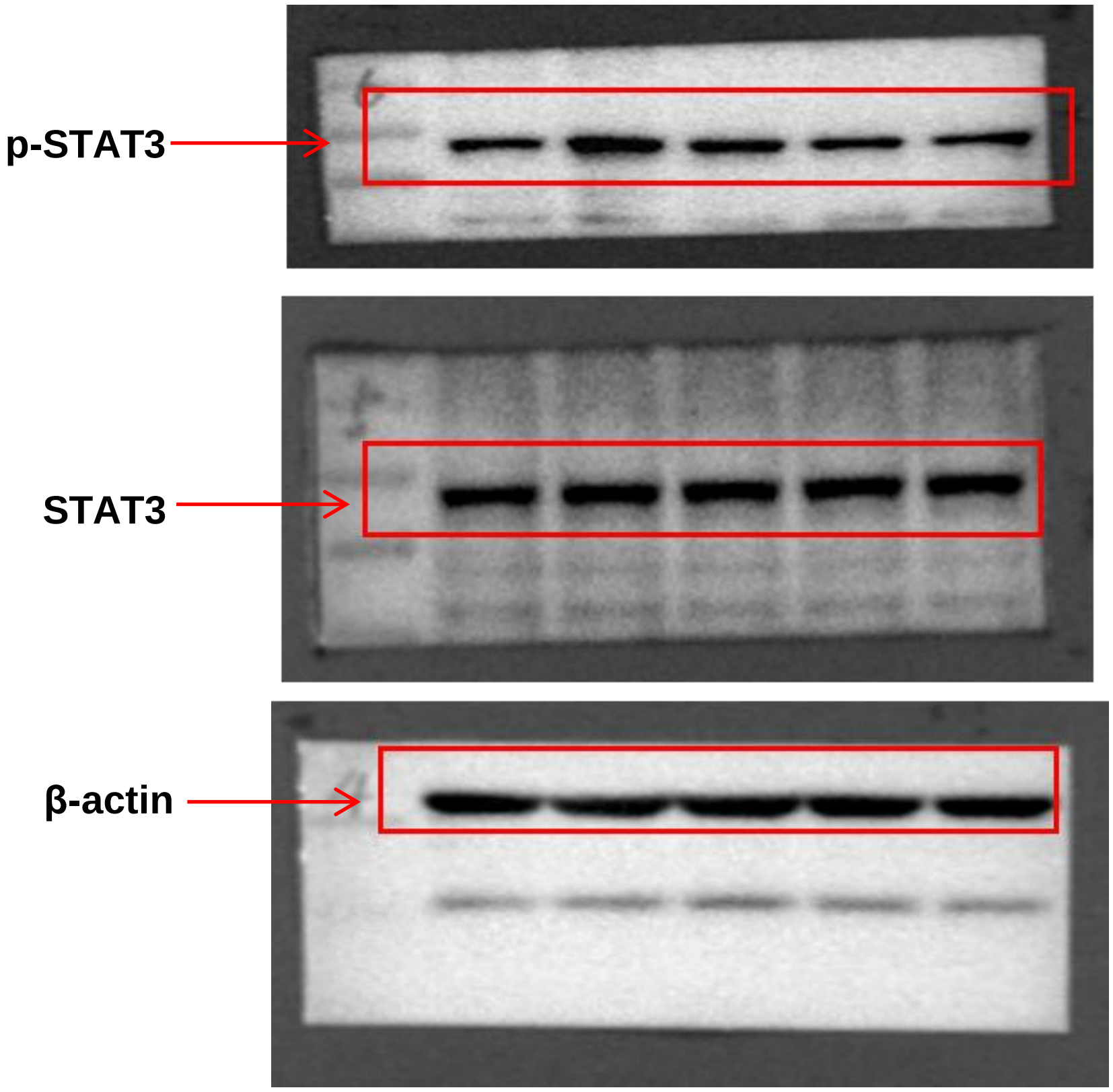

Figure 5B

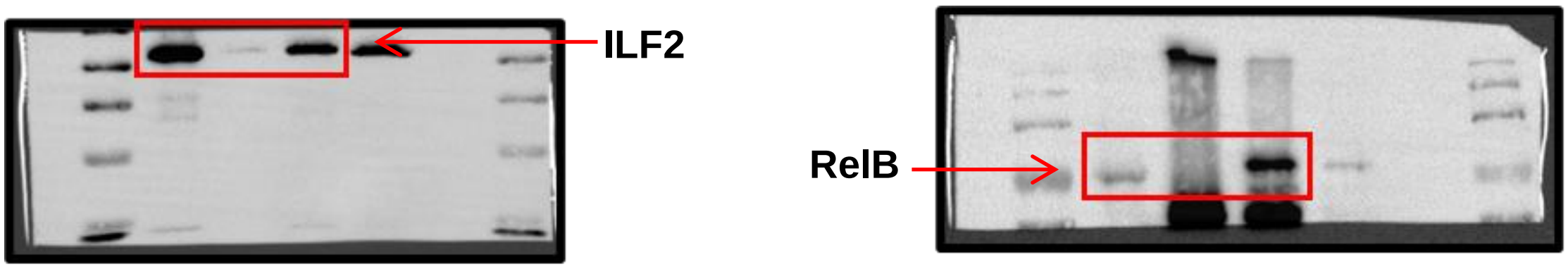

Figure 5C

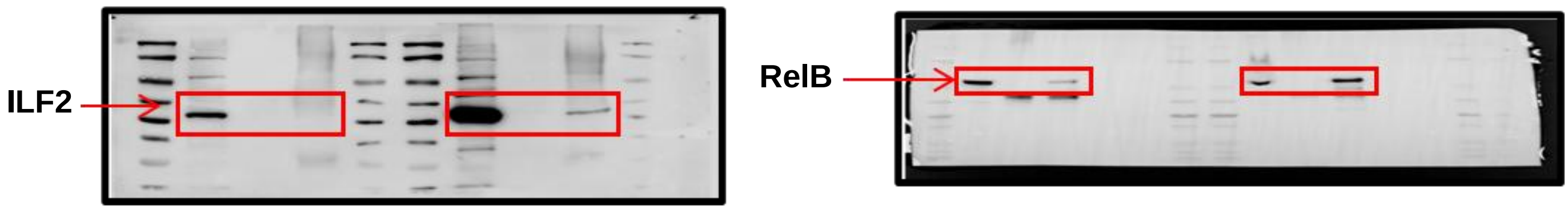

Figure 5E

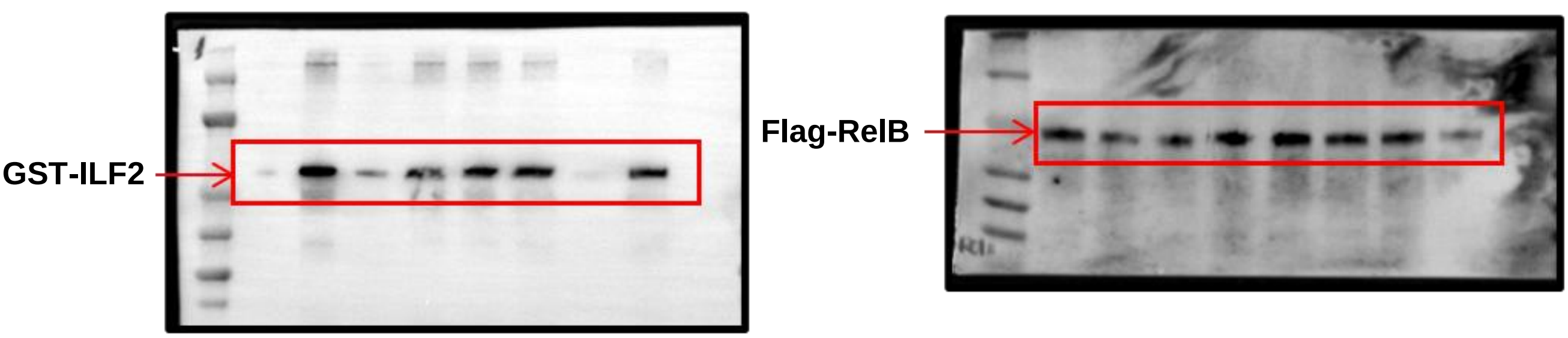

Figure 5F

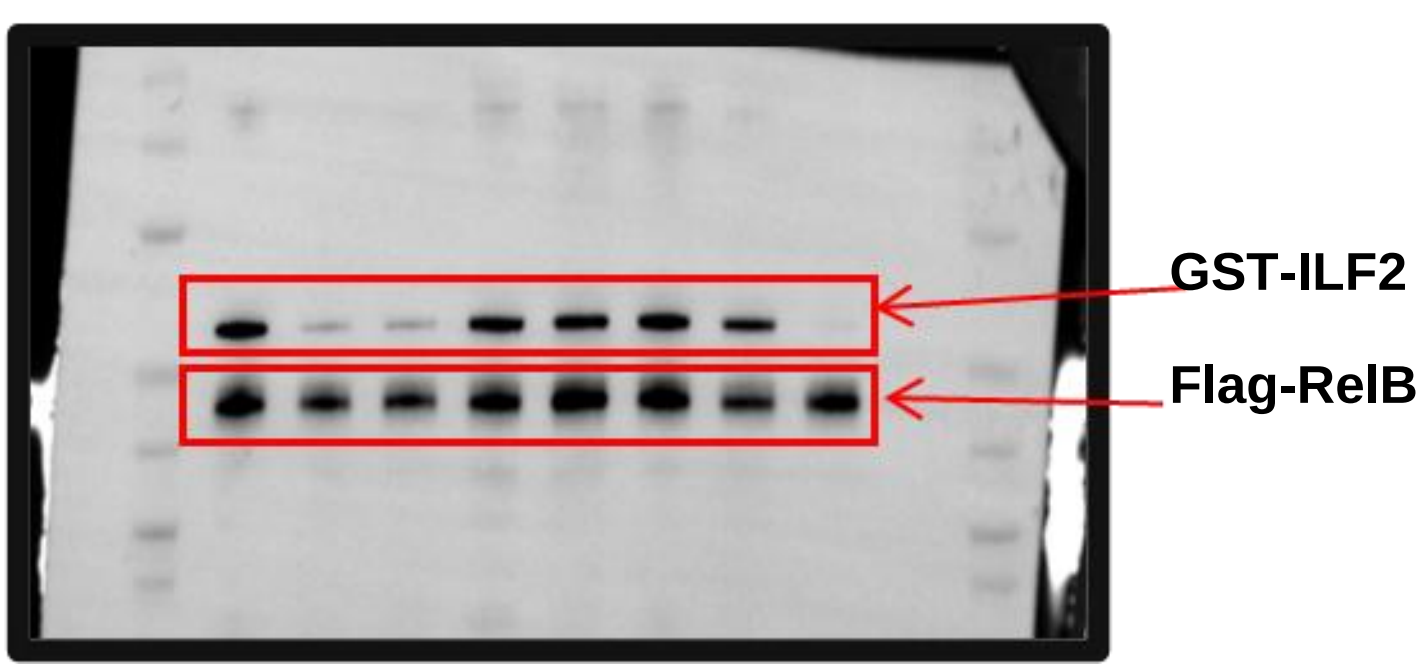

Figure 6B

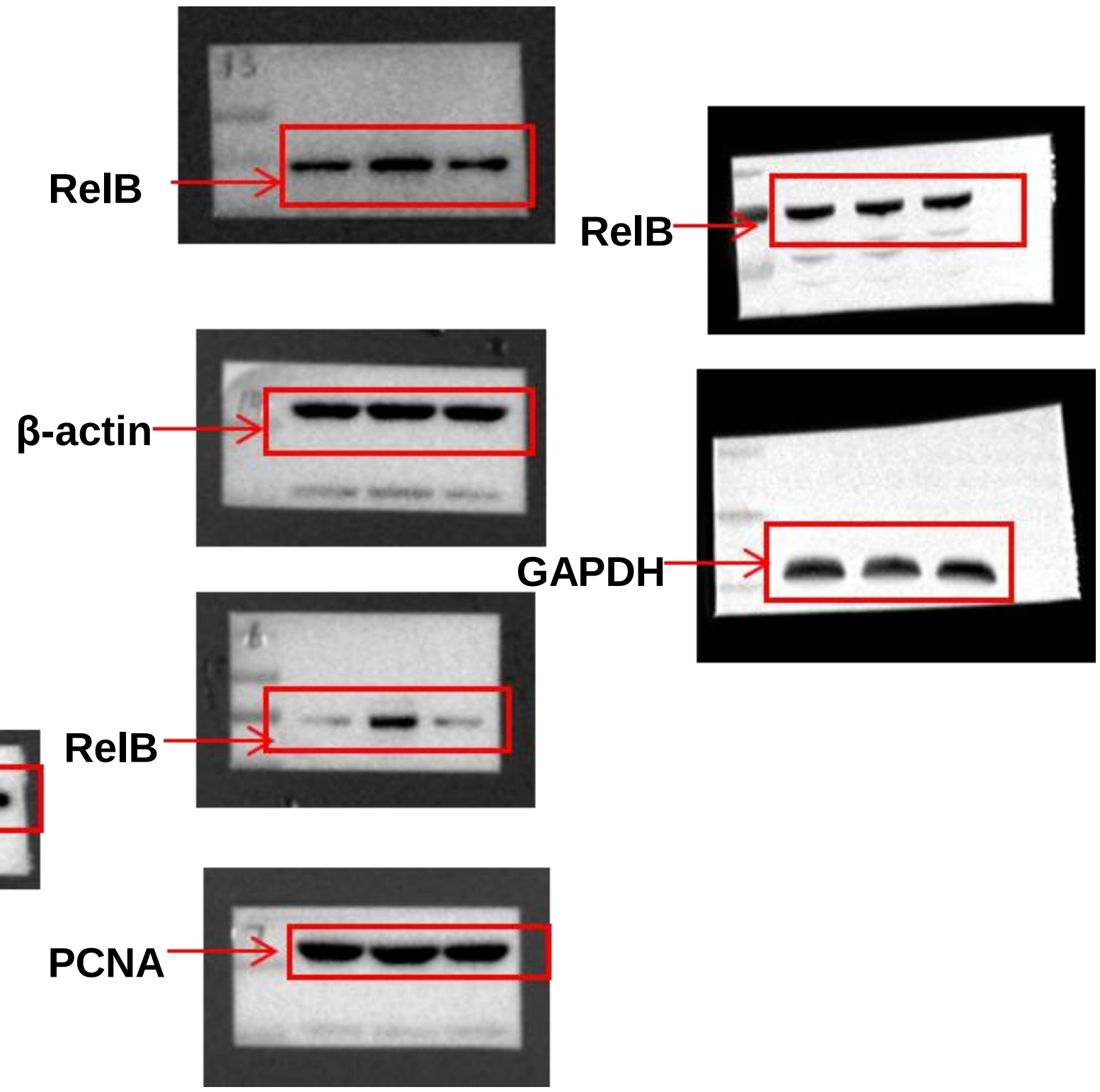

Figure 6A

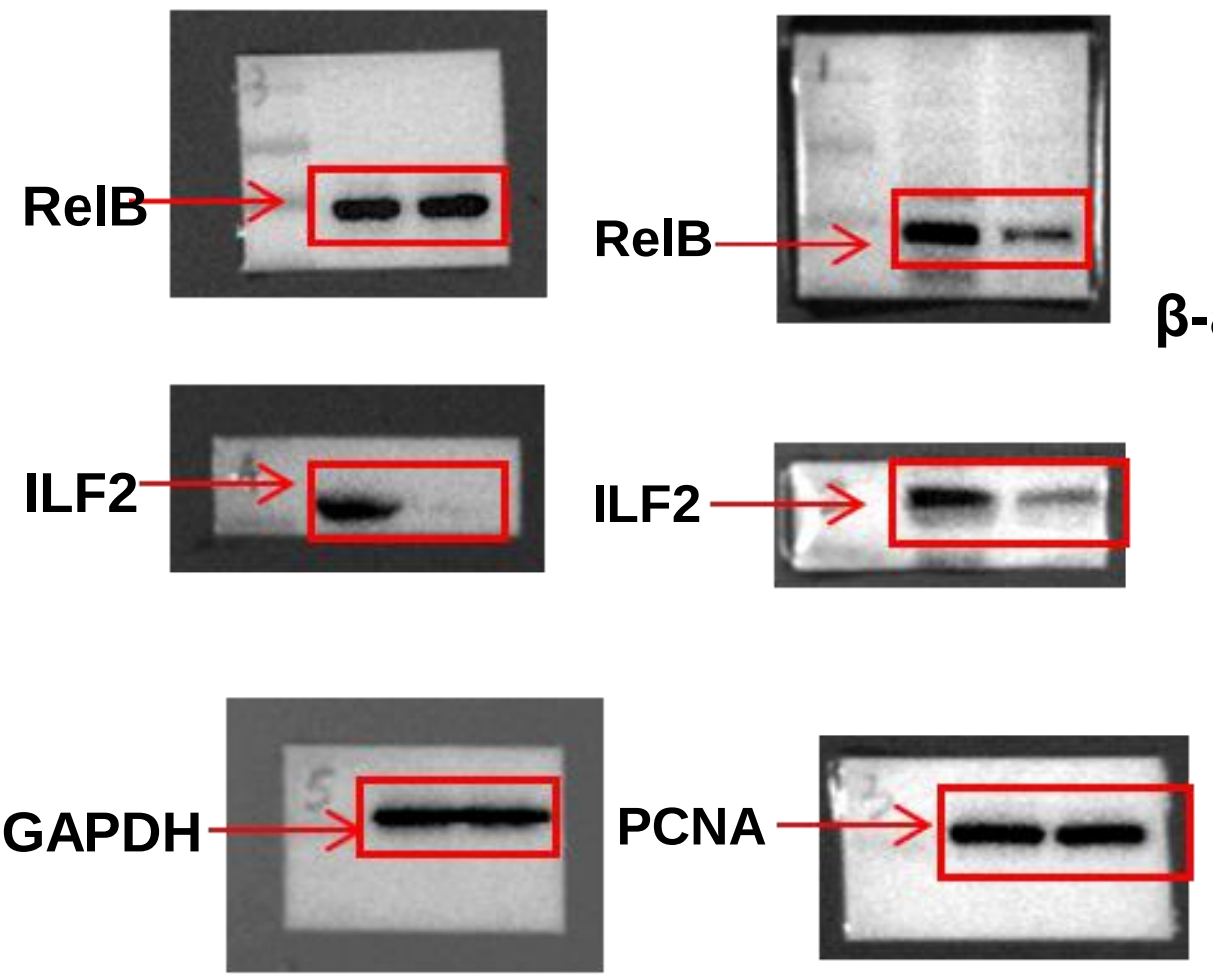

Figure 6C

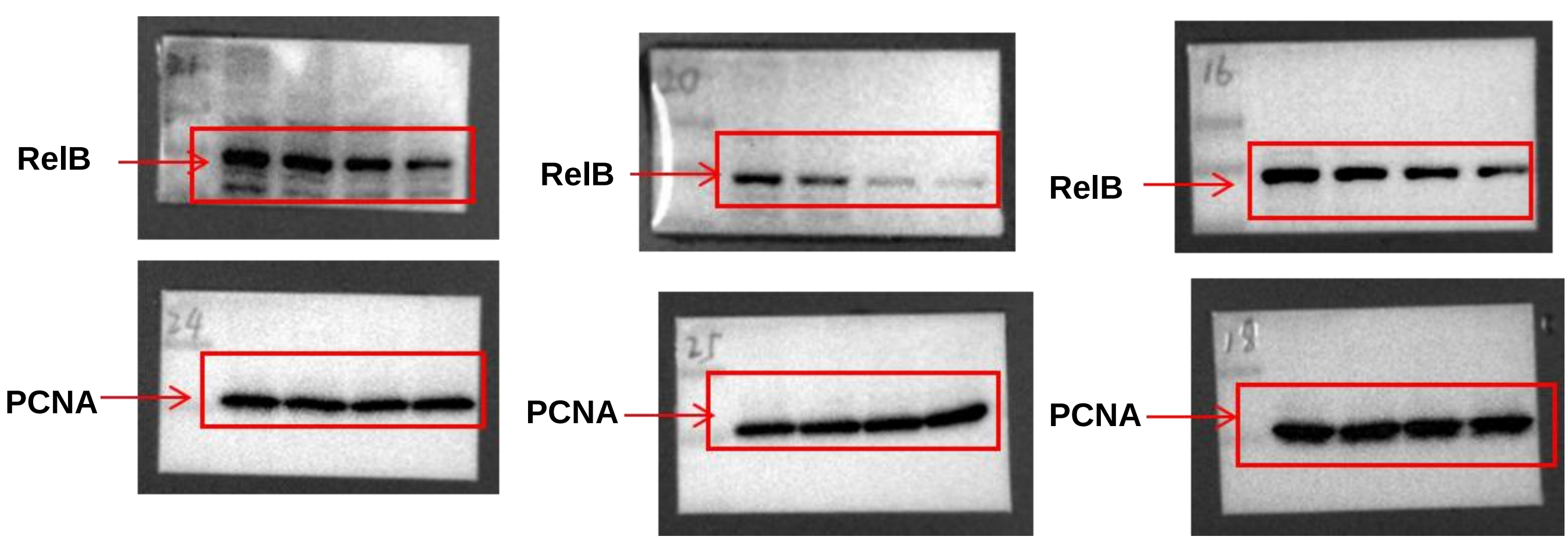

Figure 6D

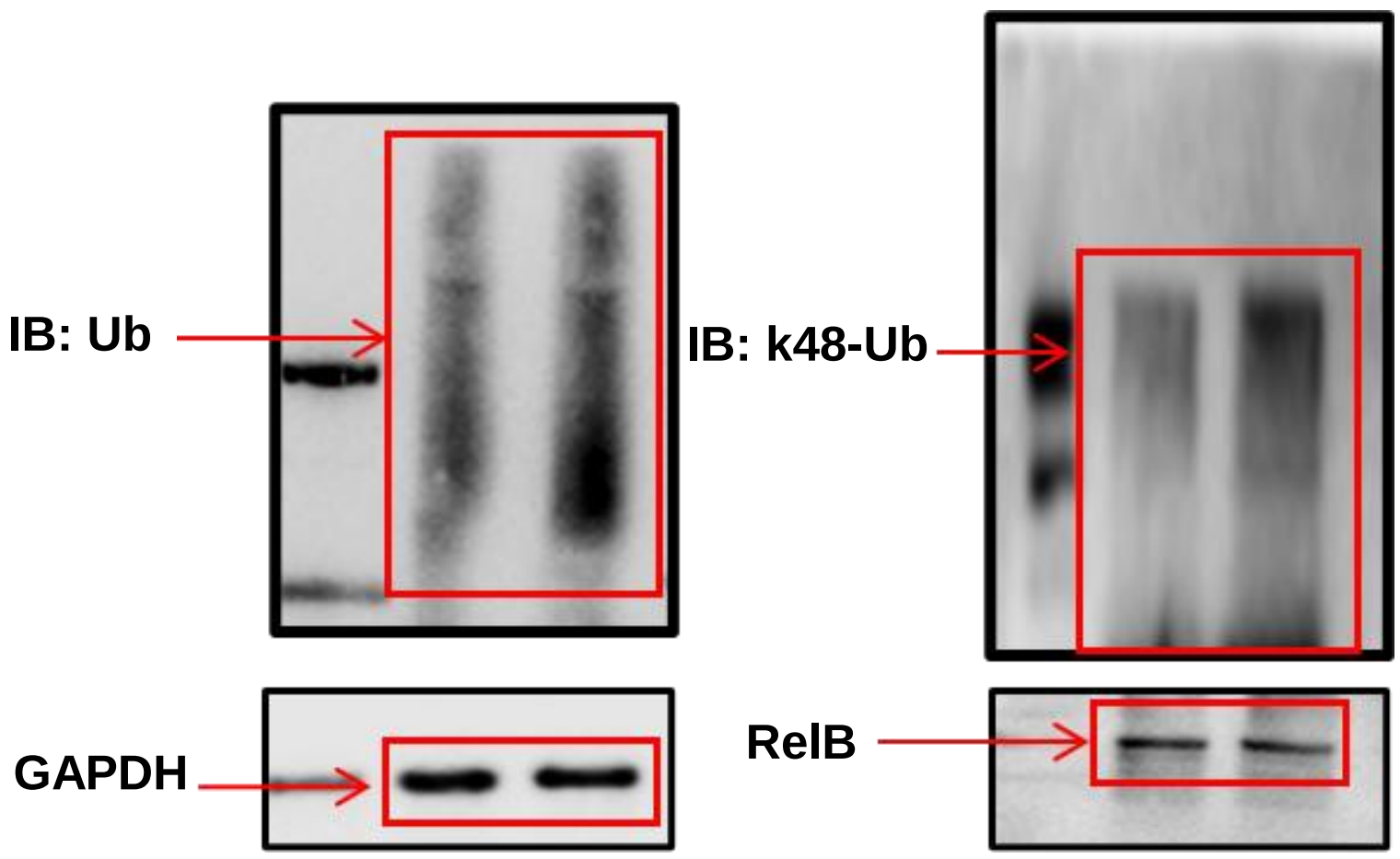

Figure 6E

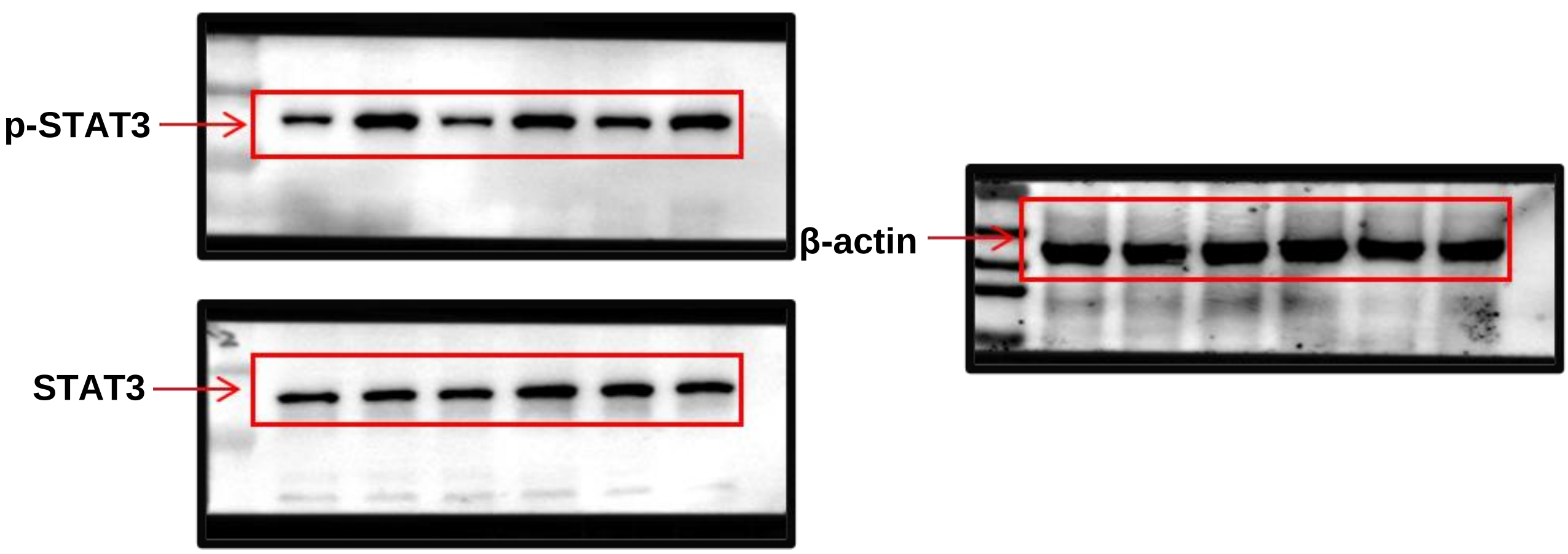

Figure 6G

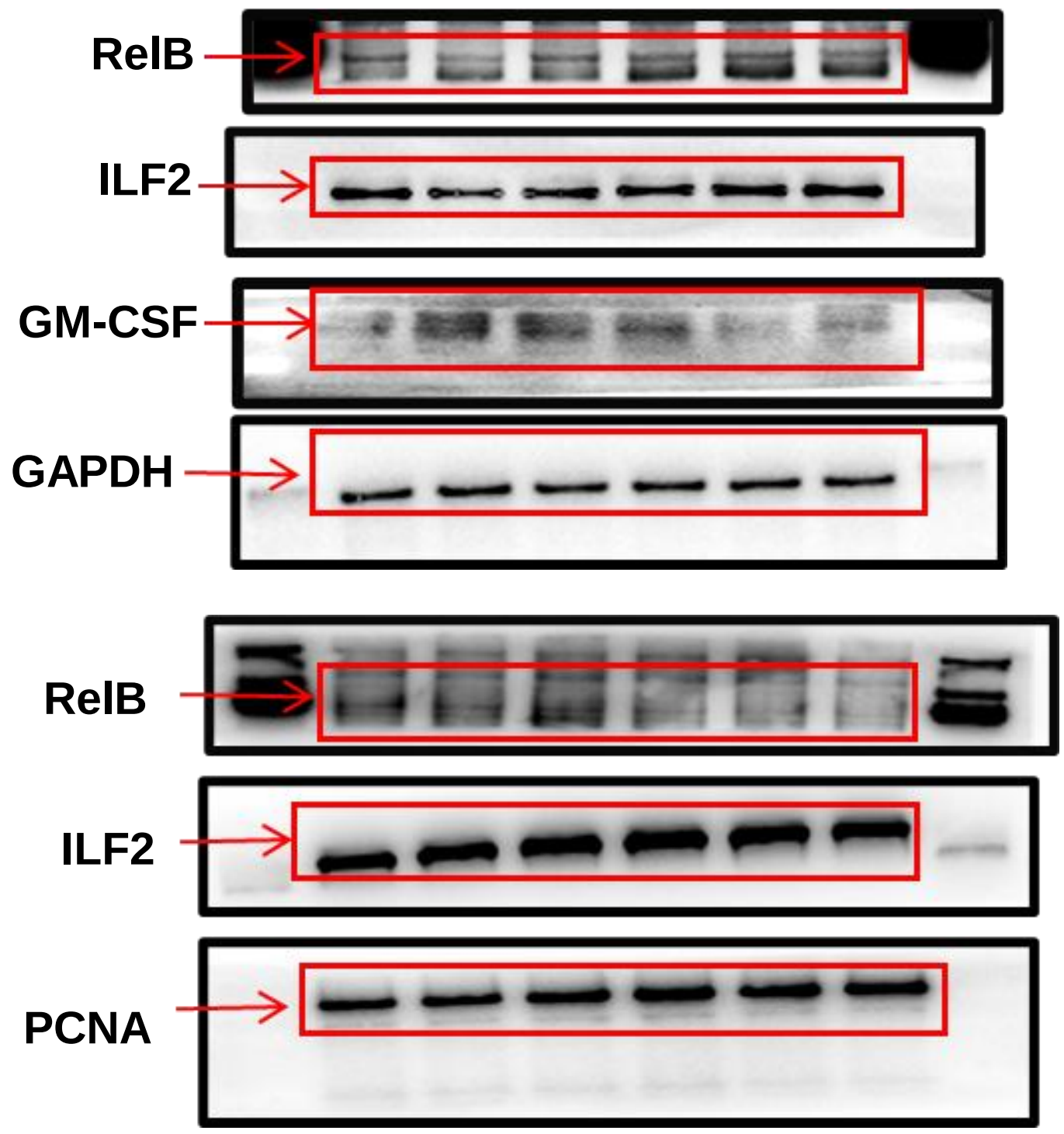

Figure 6H

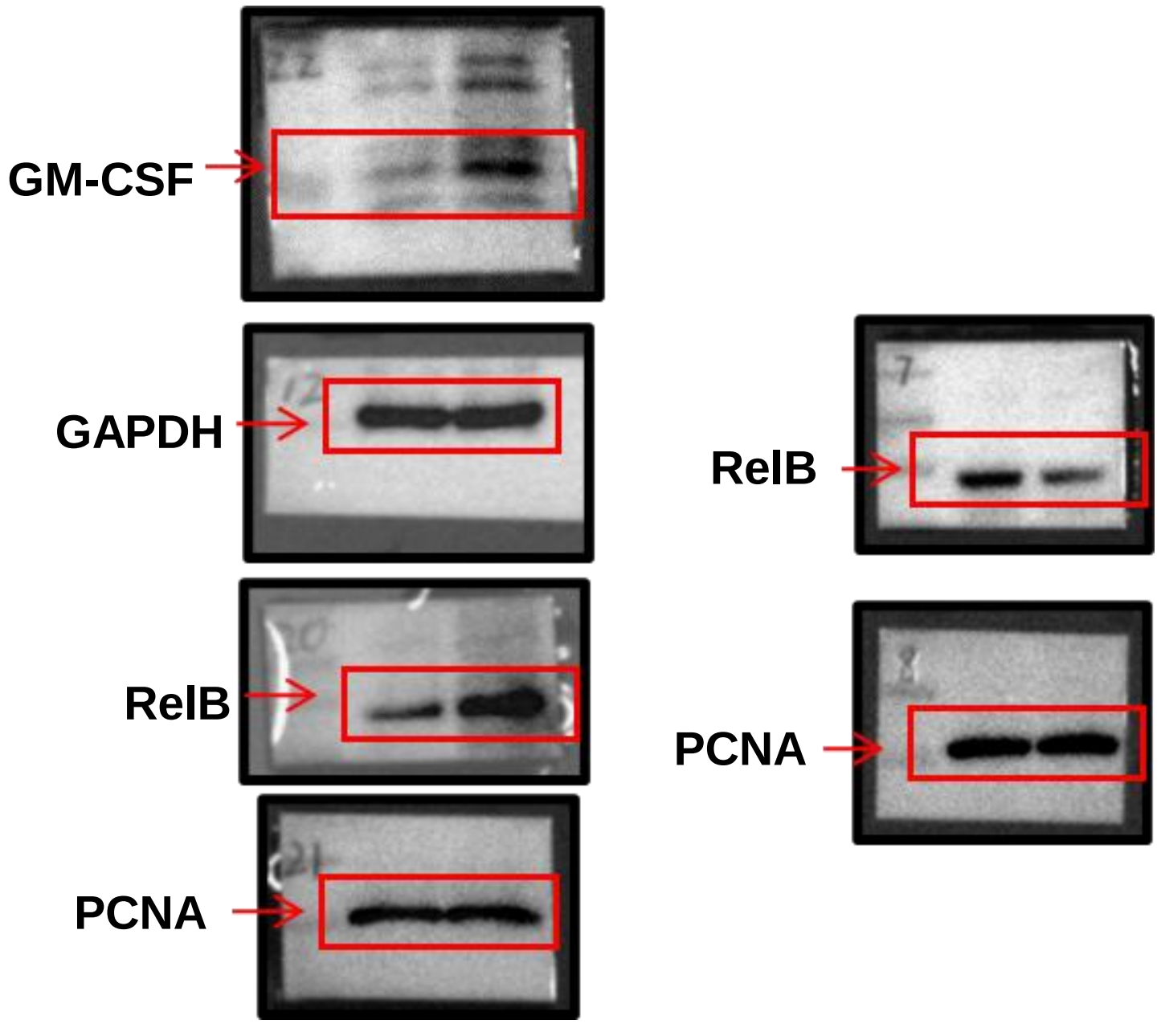

Figure S8B

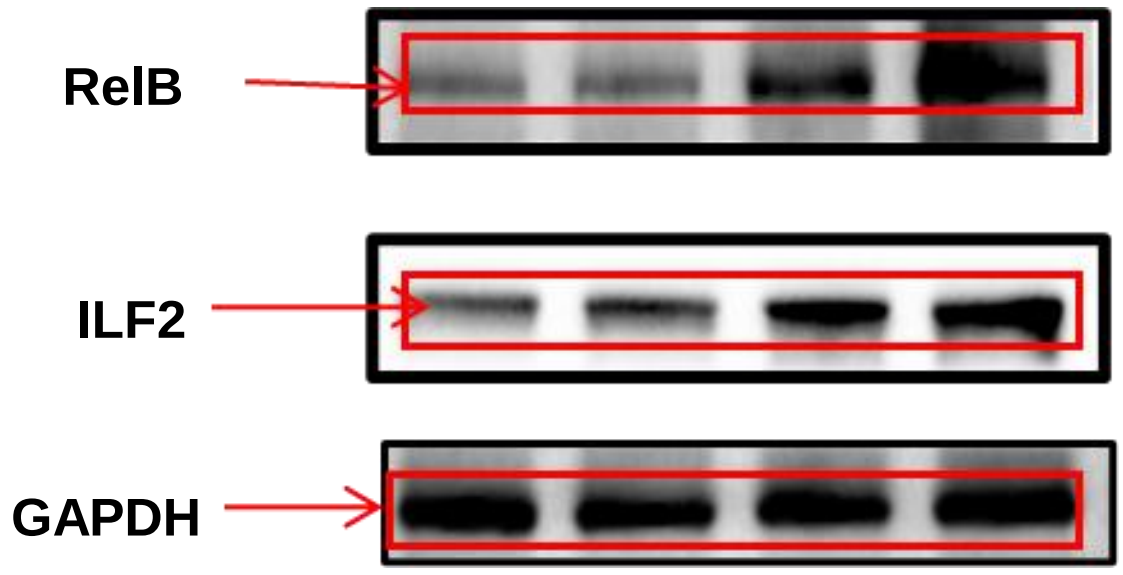

Figure S8C

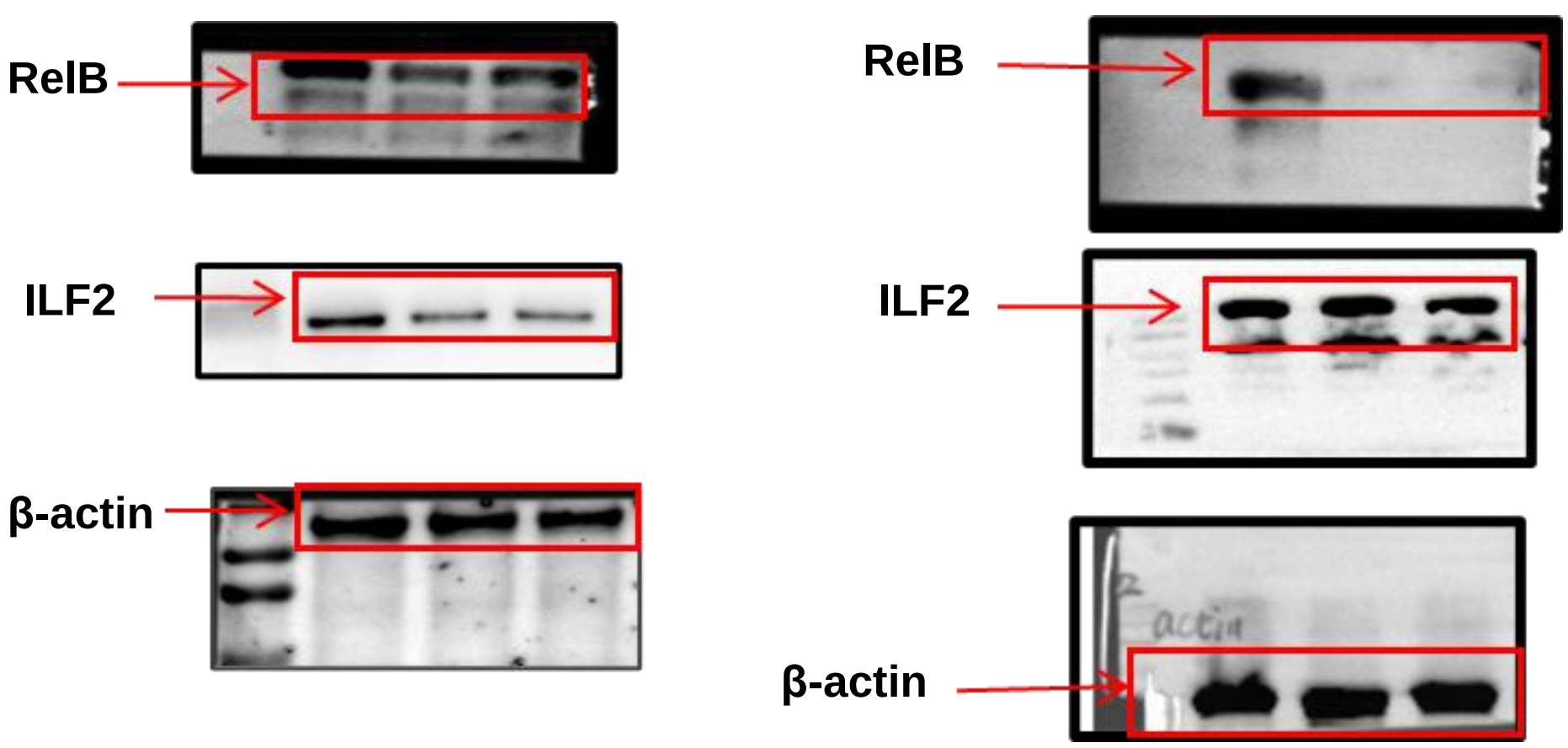

Figure S8D

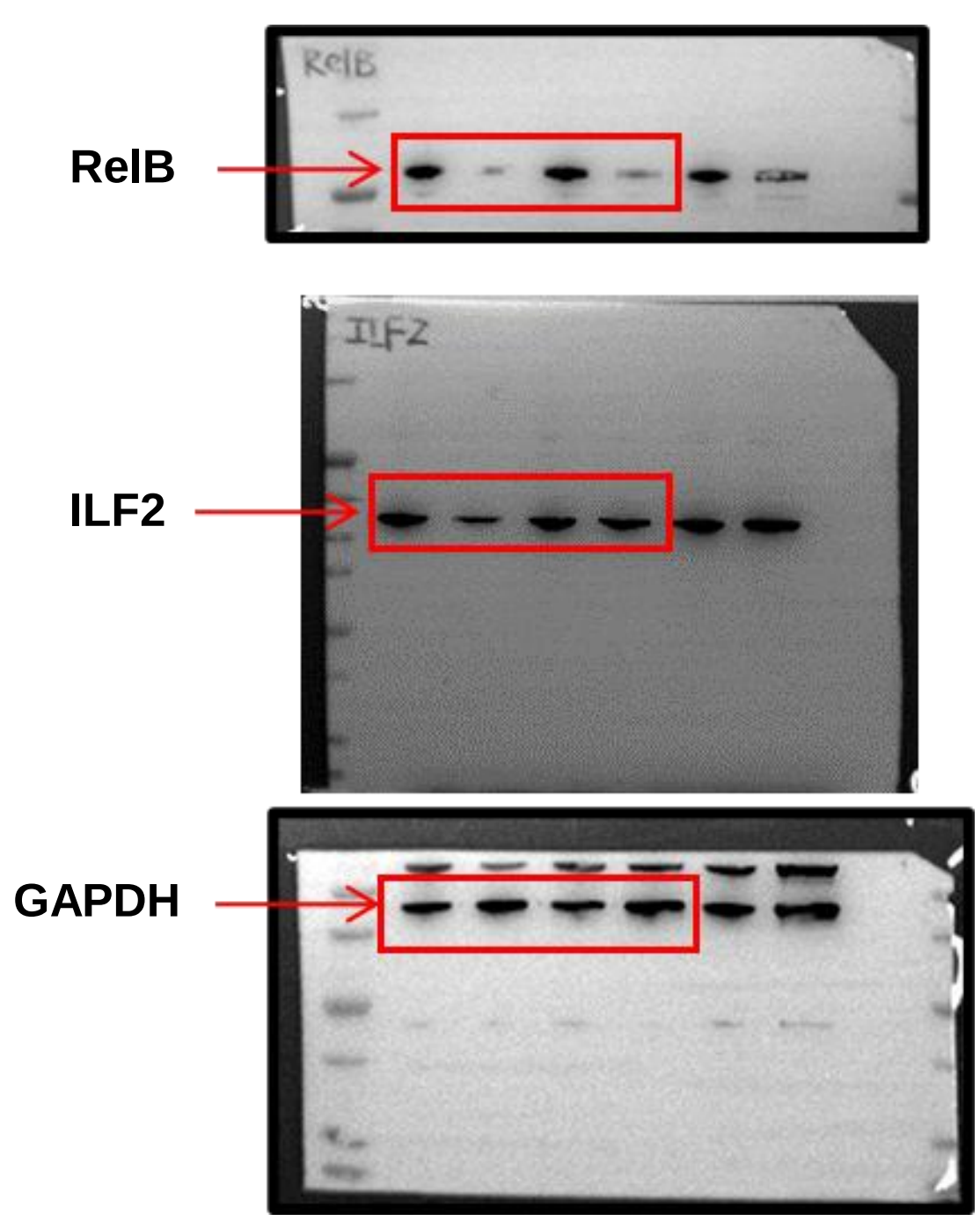

Figure S9A

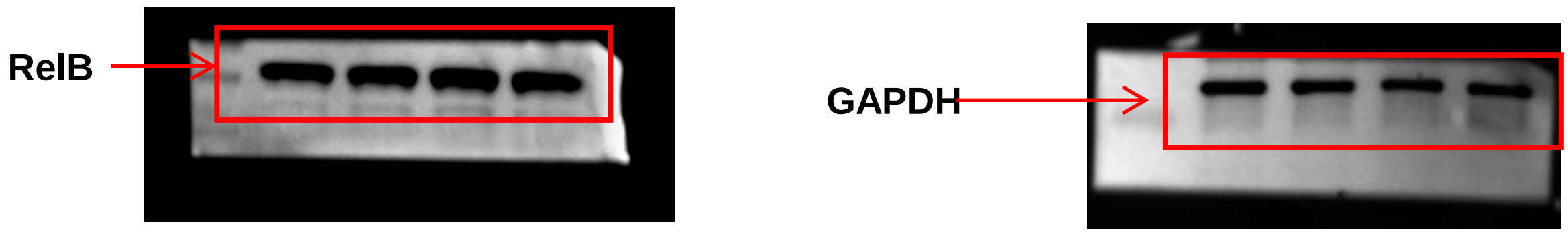

Figure S9B

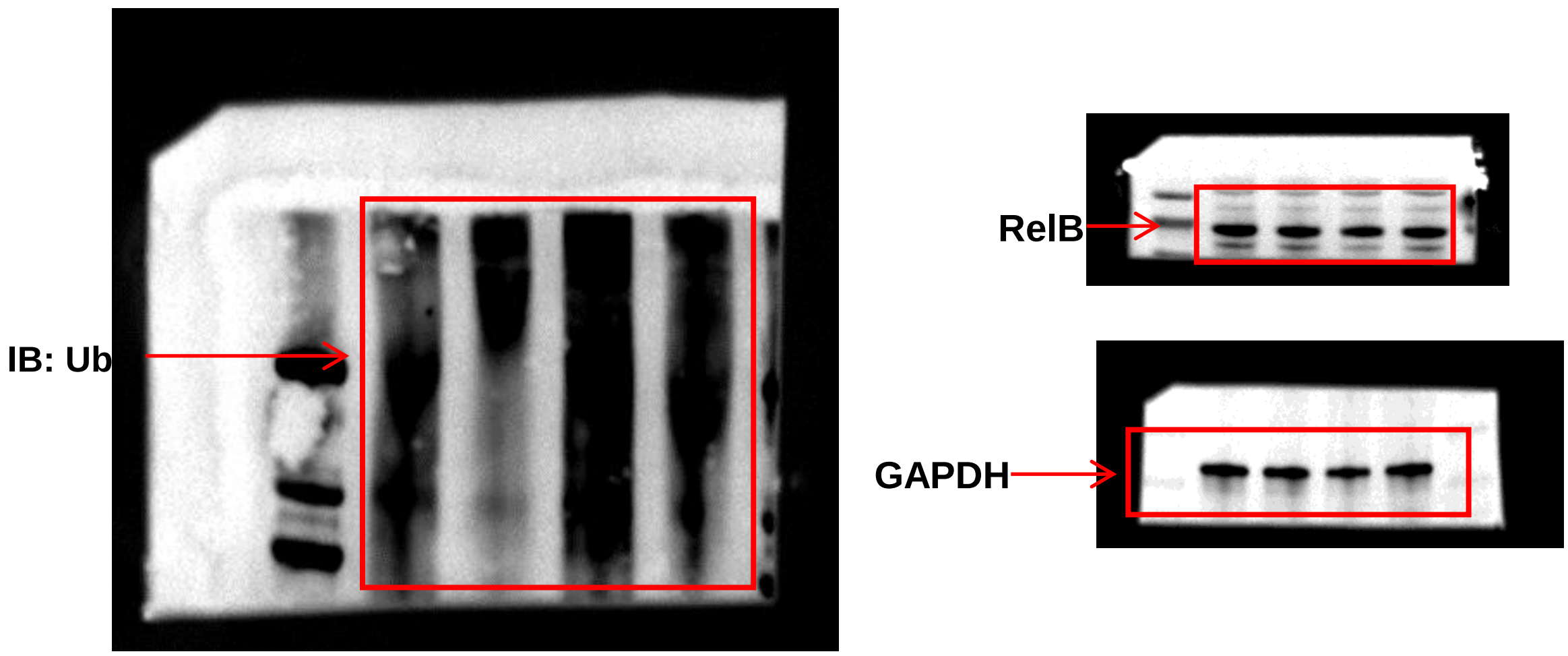

Figure S9C

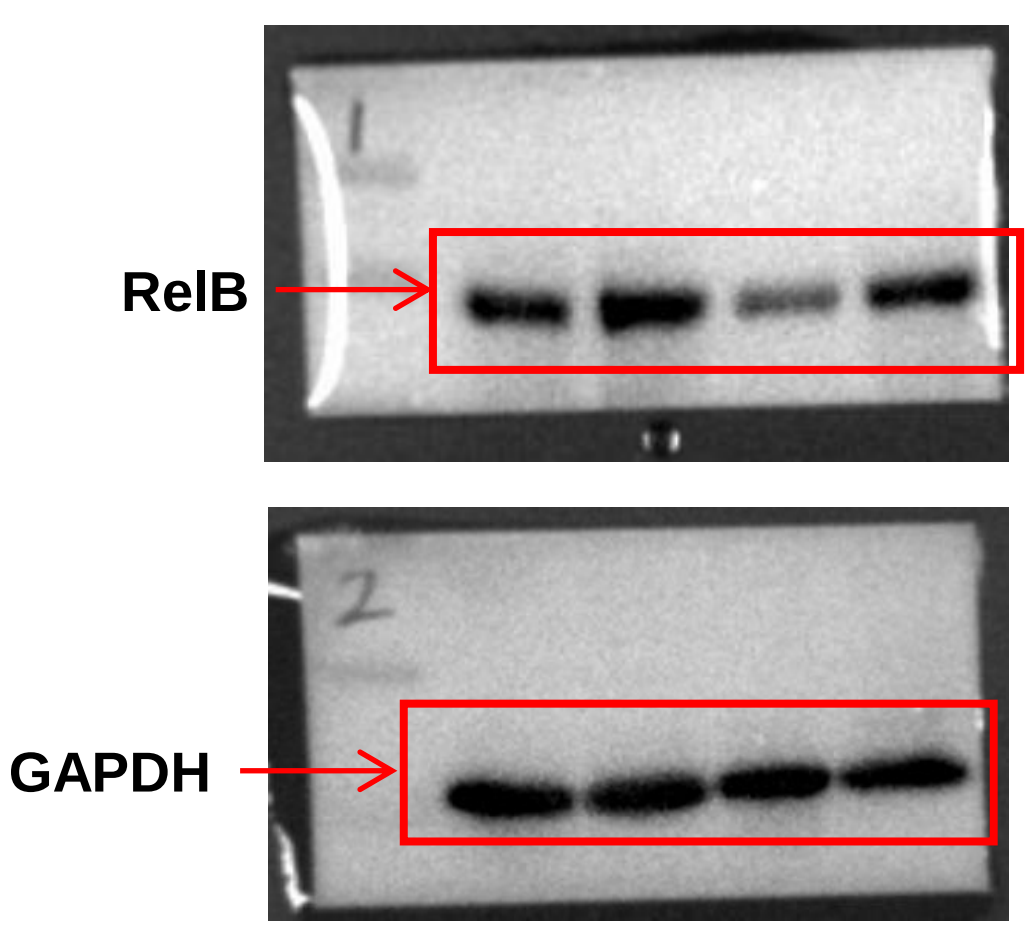

Figure S9D

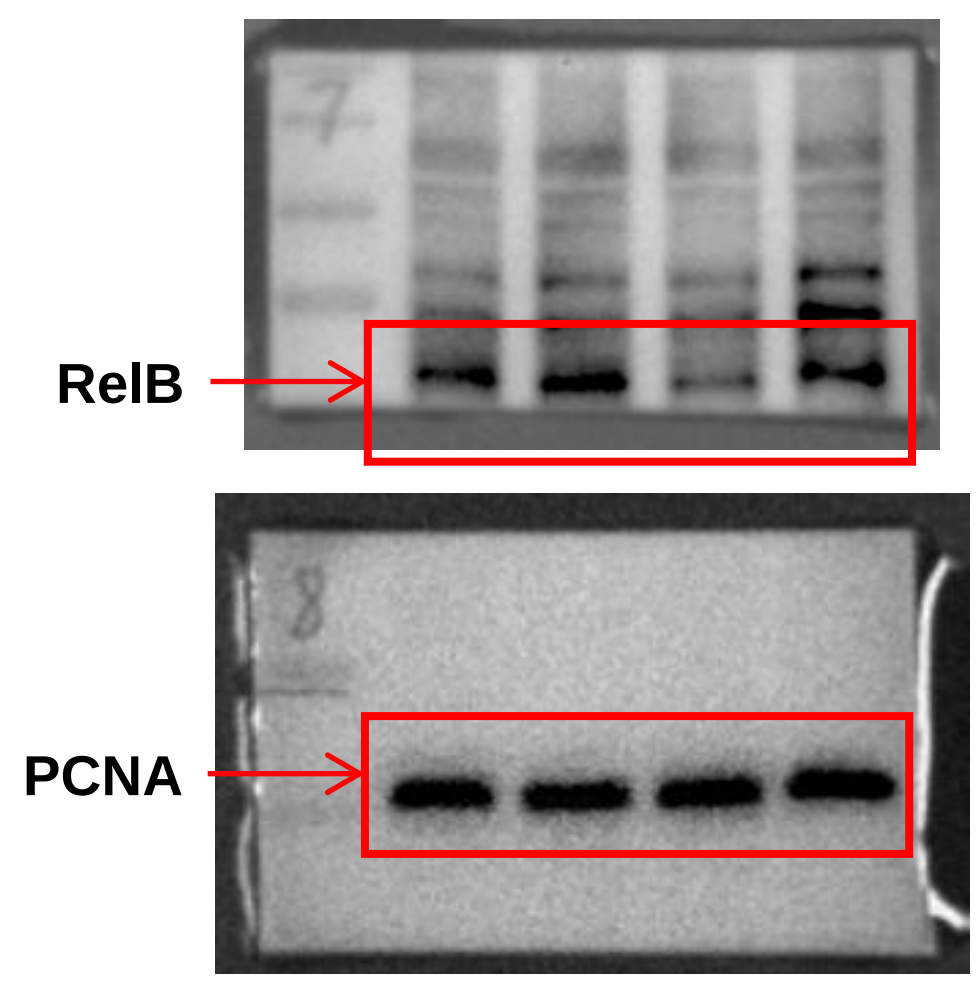

Figure S9E

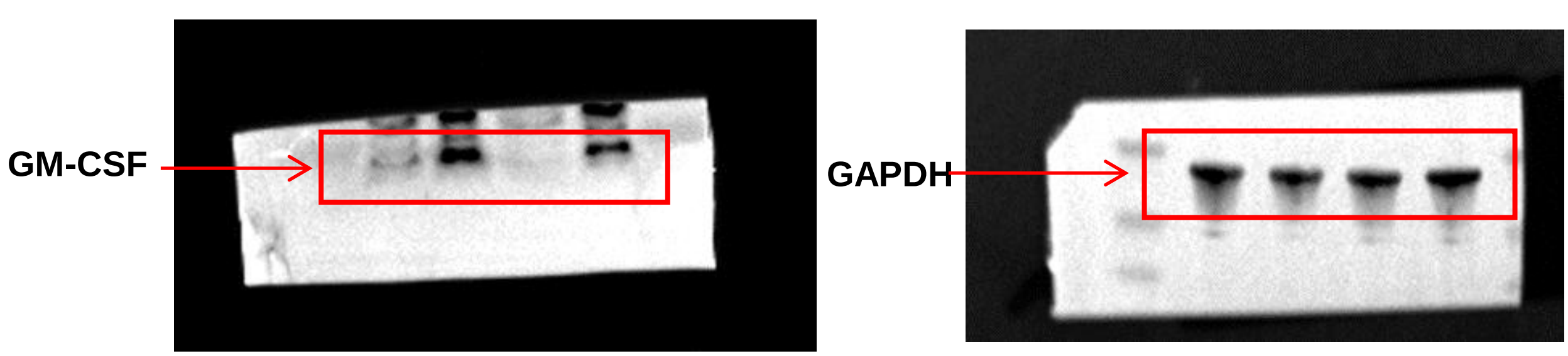

Figure S11

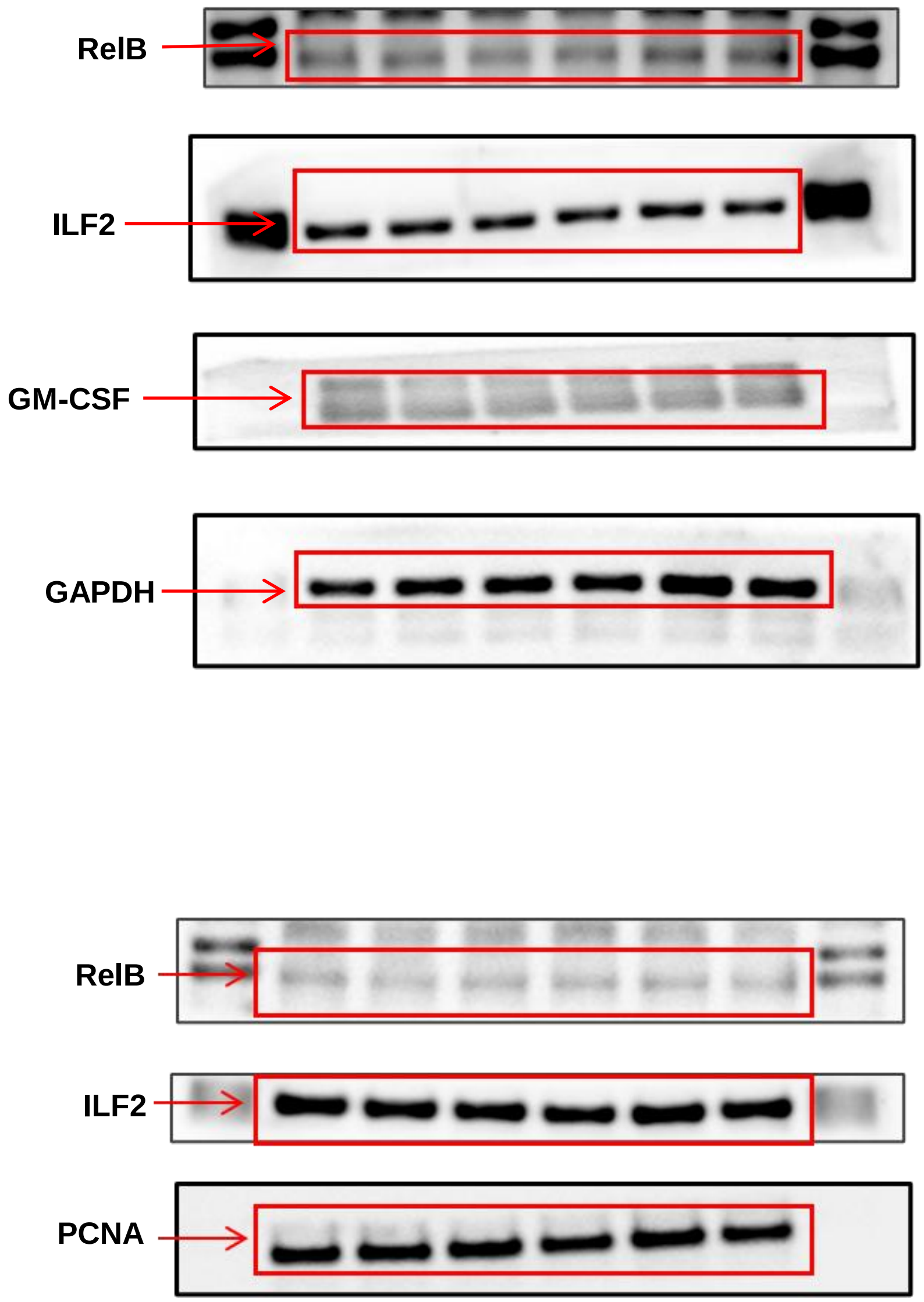

Supplement: Supplementary file 2 [file 41419_2026_9098_MOESM2_ESM.pdf]
